# Investigating the Connections Between Delivery of Care, Reablement, Workload, and Organizational Factors in Home Care Services: Mixed Methods Study

**DOI:** 10.2196/42283

**Published:** 2023-06-30

**Authors:** Adam S Darwich, Anne-Marie Boström, Susanne Guidetti, Jayanth Raghothama, Sebastiaan Meijer

**Affiliations:** 1 Division of Health Informatics and Logistics Department of Biomedical Engineering and Health Systems KTH Royal Institute of Technology Huddinge, Stockholm Sweden; 2 Division of Nursing Department of Neurobiology, Care Science and Society Karolinska Institutet Huddinge Sweden; 3 Theme Inflammation and Aging Karolinska University Hospital Huddinge Sweden; 4 Research and Development Unit Stockholms Sjukhem Stockholm Sweden; 5 Division of Occupational Health Department of Neurobiology, Care Sciences and Society Karolinska Institutet Stockholm Sweden; 6 Theme Women's Health and Allied Professionals Medical Unit Occupational Therapy and Physiotherapy Karolinska University Hospital Stockholm Sweden

**Keywords:** aging, intervention, health policy, health services administration and management, health care intervention, home care, home support, in-home assistance, personal care, policy, reablement, rehabilitation, rehabilitation medicine, social support, stress, support, systems thinking, user

## Abstract

**Background:**

Home care is facing increasing demand due to an aging population. Several challenges have been identified in the provision of home care, such as the need for support and tailoring support to individual needs. Goal-oriented interventions, such as reablement, may provide a solution to some of these challenges. The reablement approach targets adaptation to disease and relearning of everyday life skills and has been found to improve health-related quality of life while reducing service use.

**Objective:**

The objective of this study is to characterize home care system variables (elements) and their relationships (connections) relevant to home care staff workload, home care user needs and satisfaction, and the reablement approach. This is to examine the effects of improvement and interventions, such as the person-centered reablement approach, on the delivery of home care services, workload, work-related stress, home care user experience, and other organizational factors. The main focus was on Swedish home care and tax-funded universal welfare systems.

**Methods:**

The study used a mixed methods approach where a causal loop diagram was developed grounded in participatory methods with academic health care science research experts in nursing, occupational therapy, aging, and the reablement approach. The approach was supplemented with theoretical models and the scientific literature. The developed model was verified by the same group of experts and empirical evidence. Finally, the model was analyzed qualitatively and through simulation methods.

**Results:**

The final causal loop diagram included elements and connections across the categories: stress, home care staff, home care user, organization, social support network of the home care user, and societal level. The model was able to qualitatively describe observed intervention outcomes from the literature. The analysis suggested elements to target for improvement and the potential impact of relevant studied interventions. For example, the elements “workload” and “distress” were important determinants of home care staff health, provision, and quality of care.

**Conclusions:**

The developed model may be of value for informing hypothesis formulation, study design, and discourse within the context of improvement in home care. Further work will include a broader group of stakeholders to reduce the risk of bias. Translation into a quantitative model will be explored.

## Introduction

The home environment is the preferred care setting for many older adults [1]. Home care services can be offered in several instances without compromising health outcomes at a significantly lower cost as compared to institutional care [2]. Health care systems and associated payer-models differ between countries [3]. In Sweden, home care for older adults forms a part of the universal welfare system regulated on a national level by the Swedish Social Services Act (SFS 2001:453) [4,5]. Services are provided on an individual need basis and should offer high-quality care to all citizens aged 65 years and older. In practice, regulations are enacted on a local level in the regions and municipalities responsible for financing and governing home care services provided by public, nonprofit organizations, and private companies. The quality and extent of offered home care services may therefore vary across the country based on regional finances and policy [6,7].

As the overall life expectancy increases and the population distribution shifts toward older age, more pressure is being put on already resource-constrained health care systems and welfare services. Treatment outcomes are improving, meaning that the type of care needed is shifting toward management of chronic disease. Further, health care systems are moving toward distributed care models and treatment at home, enabled by technological innovations [5,8]. In Sweden, home care services are subject to increased demand and growing complexity of responsibilities and tasks. Home care staff are faced with caring for an increasing number of home care users, leading to higher workload, stress, and burnout [9].

Home care services are adapting in part by adopting goal-oriented interventions [10]. Reablement is a person-centered approach to enhance an individual’s physical and other functioning, to increase or maintain their independence in meaningful activities of daily living at their place of residence and to reduce their need for long-term services [11]. The approach has been shown to improve health-related quality of life while reducing service use [12]. Reablement is an inclusive approach irrespective of age, capacity, diagnosis, or setting, and has been used for different population groups, including a growing field of reablement for people with dementia [11,13,14]. However, there is a need to evaluate the outcomes and effects of reablement to determine its benefit in specific population groups.

Predicting the impact of change in health services is challenging. Health care can be viewed as a complex adaptive system, with intricate relationships between individual variables of the system, feedback loops, and emergent behavior. Therefore, the design of new interventions, policies, and improvement benefit from a systems perspective. Systems thinking techniques, such as causal loop diagrams (CLDs), provide a framework for studying these systems, including context and how they respond to change. The approach usually involves mapping of constituent parts (referred to as “elements”) and their causal relationships (or “connections”), analyzing feedback loops, and using simulation techniques to investigate system behavior. Reinforcing feedback loops are potential targets for policy change due to their properties as leverage points. Model development can be carried out through detailing the current body of evidence, using documentation and other knowledge bases, participatory methods with domain experts, or a combination of these. As such, the approach is useful for integrating “hard” and “soft” knowledge into the decision-making process [15-22].

This work is part of the Future Care research program, aiming to contribute to the development of knowledge-based care, participation, and social inclusion for older adults. This includes studies on the working environment in home care, reablement with the support of information and communication technology (ICT; the ASSIST project), social participation, the design of physical spaces, and more [23,24].

The aim of this study is to characterize the home care systems elements and their connections relevant to home care staff workload, work-related stress, home care user needs and satisfaction, and the use of enabling technologies. This is to provide a systems model for examining the effects of interventions in home care, including the reablement approach and accompanying ICT (ASSIST, Future Care) [24], disabled home care users, home care staff, and organizational factors. As such, the project intended to take a broad, holistic perspective to improvement in the home care setting. A CLD was developed grounded in expert knowledge and validated instruments. The developed model was then verified and analyzed.

## Methods

### Overview

**Figure 1:**
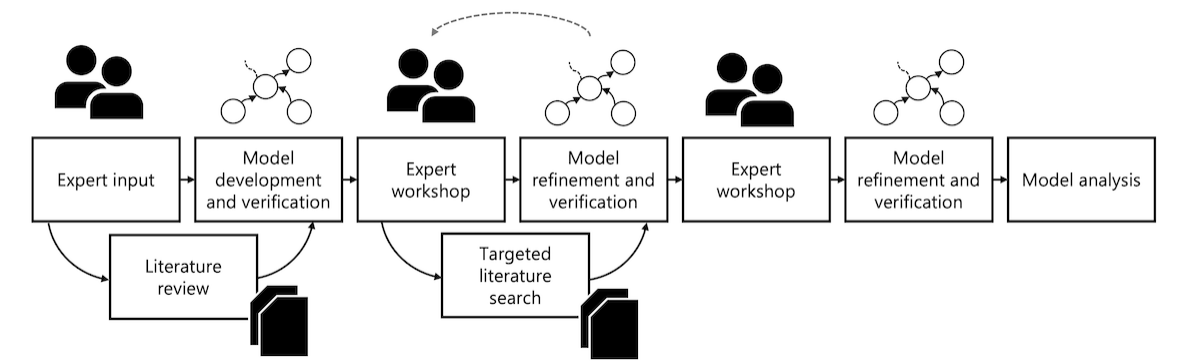
Workflow of the iterative model development process.

Various approaches have been used for participatory model development [21,25]. This study used an iterative approach, combining group model building and targeted data collection (for additional details, see section S1 in Multimedia Appendix 1 [12,26-49]). In total, 11 experts participated across the activities. The experts were all active academic health care science researchers in nursing and occupational therapy, focusing on aging, home care, nursing homes, health services for older adults, social participation, public health, evidence-based care and the reablement approach. The participants did not include home care staff, older people in home care or their relatives. No personal data were collected, accessed, or analyzed for this study. Experts were engaged in their work setting and professional role to collect their views. Participation was voluntary and could be terminated at any time. No track records between individuals and their statements have been kept beyond the initial data collection. Model development was preceded by input from the experts to define the requirements, scope and boundaries, key scientific literature, and documentation to support model development and verification. This was followed by collection of literature data, model development, and verification against intervention data sets. Expert review was carried out at 2 separate occasions, this to assess the model structure, literature sources, and verification results. Following each expert review phase, additional targeted literature searches were carried out along with model refinement. Finally, model analysis was carried out by analyzing feedback loops, the model behavior using social network analysis and simulations (Figure 1).

### Ethical Considerations

The research only involved the opinions of professionals in a professional setting, so no ethical approval was necessary according to the Swedish Ethical Review Act through the Swedish Ethical Review Authority [50].

### Requirements, Scope, and Boundaries

The model requirements and scope were defined together with the domain experts. It was decided that the model should be able to describe home care staff workload, work-related stress, provision of care and services, home care user needs, and satisfaction. Relevant elements included those linked to the home care organization, home care staff, and users. The purposes of the model were to enable further analysis of the data being generated within the research program and to allow the study of interventions related to the reablement approach and associated technologies.

Key literature on the reablement approach and workload or job strain, including the questionnaires and theoretical models, QPSNordic (the General Nordic Questionnaire for Psychological and Social Factors at Work), and SDCS (Strain in Dementia Care Scale) [51,52] were identified a priori by the experts. QPSNordic describes items related to social and psychological factors in the workplace, including leadership, organization, control and demand, social climate, role conflict, and more. The survey is used to investigate work conditions and health and support organizational change. The SDCS describes staff strain in residential dementia care; this includes variables and their impact on job strain, including balancing and competing needs, frustrated empathy, understanding and interpreting, emotional involvement, and recognition. SDCS was designed to aid the identification and study of interventions to improve staff well-being in residential care, among others. The survey tool has also been applied in the home care setting [53]. Preliminary literature searches were carried out to identify suitable models of work-related stress for the home care setting, including the stress of conscience and quality of care.

### Model Development

Data collection was carried out using MEDLINE and PubMed. This is to identify quantitative and qualitative predictors of stress in home care, residential care, care for older people, dementia care, nursing homes, and related settings (for more information, see Multimedia Appendix 1). A CLD was developed in Kumu (Kumu Inc). The final model can be viewed as an interactive map [54]. The data set with the full reference list (Table S4 in Multimedia Appendix 2 [20,52,54-110]), the model export file and analysis (Tables S5 and S6 in Multimedia Appendix 3), and the simulation script (Matlab script file in Multimedia Appendix 4) are also available as supplementary material. The stress model was developed based on theoretical models of work-related stress derived from the preliminary literature search and discussions with experts [51,111]. Additional literature data were incorporated into the model by reviewing the structure of the model and adding new elements and relationships one at a time. This was carried out in several iterations to ensure consistency between data and CLD. Here, the inclusion of studies from nursing homes and residential care was justified as important supplemental data in the absence of evidence from the home care setting. An expert review was carried out to ensure relevance to home care (section S4 and Figure S5 in Multimedia Appendix 1). Model elements were grouped into categories. The final categories included organization, home care staff, home care users, stress, social support network (of the home care user), and societal level (Table 1). The basis of this categorization was the conceptual level of the individual elements, as per Dallner and colleagues [111].

**Table 1:** Categories of the causal loop model along with their description.

Categories	Description
Stress	The core stress model, describing stress response as a combined effect of job demand and control.
Home care staff	Describes the delivery of care and services, professional competence and experience, and direct interactions with the home care organization. In addition, this includes elements of the home care staff’s private life and the impact of stress on mental and physical health.
Home care user	Describes home care user needs, experience and expectations, physiological and mental health, and direct interactions with home care staff and social support network.
Organization	Includes leadership and organizational elements, home care strategies for delivering care and services, work planning and scheduling, and direct interactions with home care staff.
Social support network (of the home care user)	Describes the home care user’s social network, their experience, and interactions with the home care user, and how this influences informal care.
Societal level	This includes higher-level elements that are extrinsic to the home care organization, home care staff, and user. This category includes the impact of regional unemployment, income, and care capacity on home care, stigmatization toward the profession, and the effect of home care on health care spending.

### Model Evaluation and Refinement

Model evaluation and refinement were carried out on 2 occasions. This consisted of verification of the model structure based on the identified studies of interventions and outcomes, expert input through participatory workshops, and model refinement based on feedback.

Verification was carried out based on identified literature on relevant clinical intervention outcomes in nursing homes and home care (including the reablement approach). This was carried out by identifying and qualitatively comparing scenarios and outcomes with model elements representative of the intervention along with the cascade reaction produced in the model and its ability to recover the observed outcomes.

### Model Simulation and Analysis

Social network analysis was carried out [55]. Methods and results of the social network analysis are detailed in Multimedia Appendix 1. A simplified simulation algorithm based on Boodagian and colleagues [112], was implemented in Matlab (release 2022a; Mathworks). Key elements of interest (relevant to the study aims) were investigated for their ability to influence the model as a whole; these included “needs met,” “provision of care and services,” “workload,” “distress,” “person-centered care” (ie, the reablement approach) and “home-care-staff user adoption of home care technology” (ie, ICT to support the reablement approach). The full set of simulation results is detailed in section S6 in Multimedia Appendix 1.

## Results

### Overview

**Figure 2:**
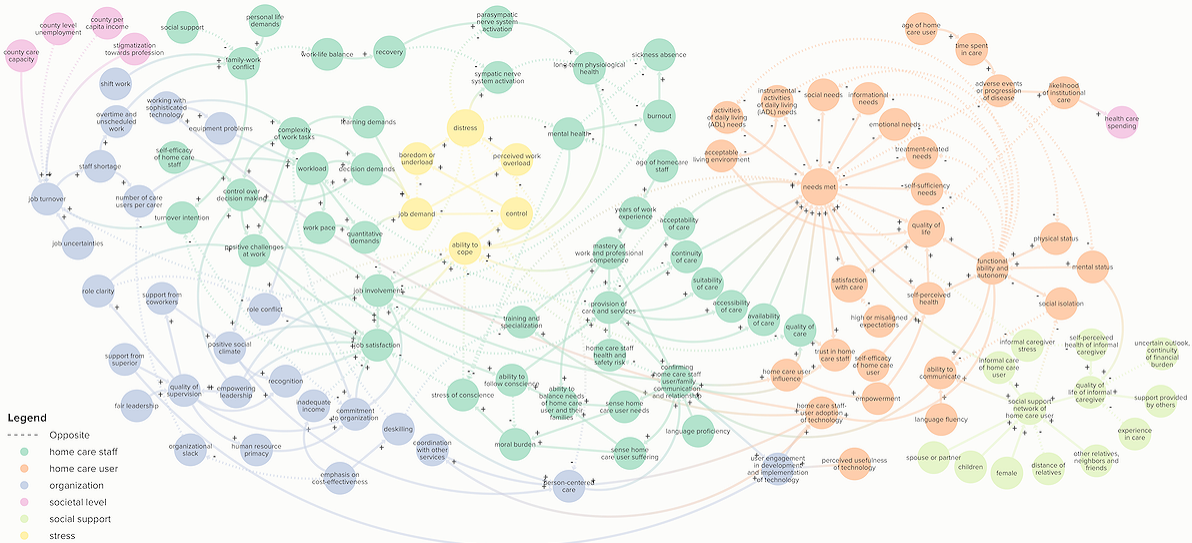
The final full causal loop diagram of home care elements and their connections. Arrows indicate the directional connections between elements. Positive and negative relationships are displayed in solid arrows (+) and dashed lines (–), respectively.

The review of the literature identified 914 nonunique relationships between relevant variables in home care for older people, based on 59 publications (Table S4 in Multimedia Appendix 2). Additional knowledge was considered through participatory model development with academic experts. The final model included 122 elements and 223 connections divided across six categories (defined in Table 1): (1) stress (elements: n=6), (2) home care staff (n=44), (3) home care user (n=28), (4) organization (n=26), (5) social support of the home care user (n=13), and (6) societal level (n=5; Tables S5 and S6 in Multimedia Appendix 3). Figure 2 shows the full CLD.

### Stress

Several theoretical models of work-related stress were identified in the literature [113-116]. One of the most well-established theoretical models chosen to describe work-related stress here is the demand-control model developed by Karasek [117].

According to demand-control theory, job strain and negative stress (distress) will increase when the impact of demand outweighs control. Further, an interaction effect has been observed between control and demand on stress, where an increase in control outweighs the effect of demand [118]. This was implemented by allowing control and demand to affect both elements’ underload and overload.

**Figure 3:**
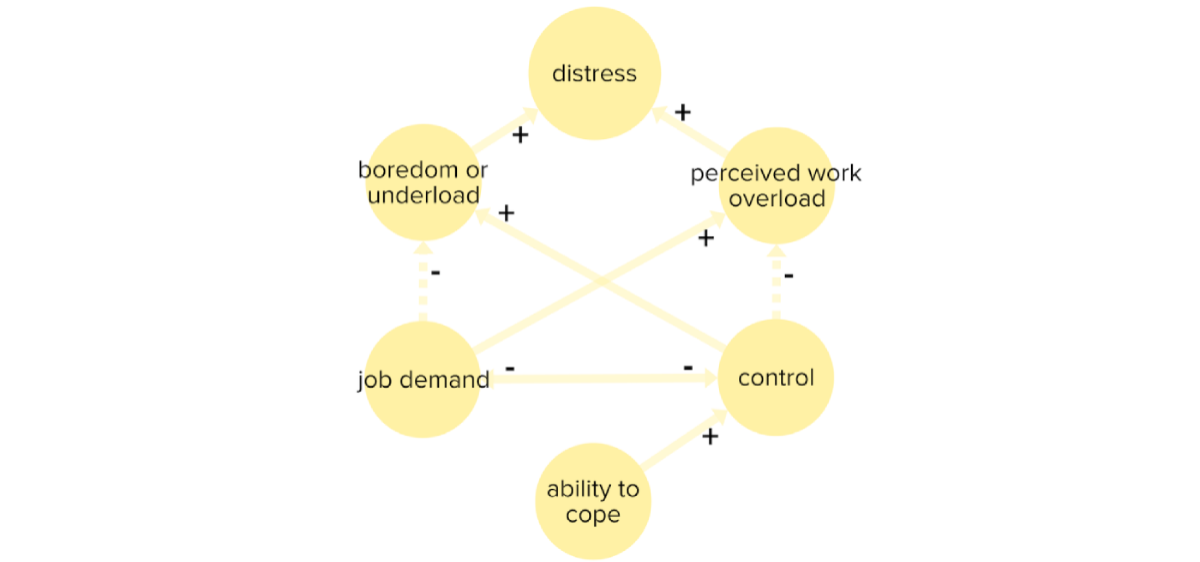
The simplified stress model describing the impact and relationship between job demand, control, and distress. Circles indicate elements and arrows are connections between elements. Positive and negative relationships are displayed in solid arrows (+) and dashed lines (–), respectively.

It was agreed in the expert group that a model of job strain and stress should account for a nonmonotonous (U-shaped relationship) between stress and demand and control. This means that when demand is higher than control, distress occurs through work overload. When control outweighs demand boredom, induced by work underload, becomes a potential source of distress (underload; Figure 3). Multiple home care staff elements linked to the stress model. For example, several types of demand affected “job demand.” While the “ability to cope” was affected by “mastery of work and professional competence,” “job involvement,” “job satisfaction,” and through feedback, by the stress response. “Recovery” indirectly affected the “ability to cope” positively. The element was reliant on home care staff, “social support,” “personal life demands,” “shift work,” and “overtime and unscheduled work.” Additional information is given in section S2 in Multimedia Appendix 1.

### Home Care Staff

**Figure 4:**
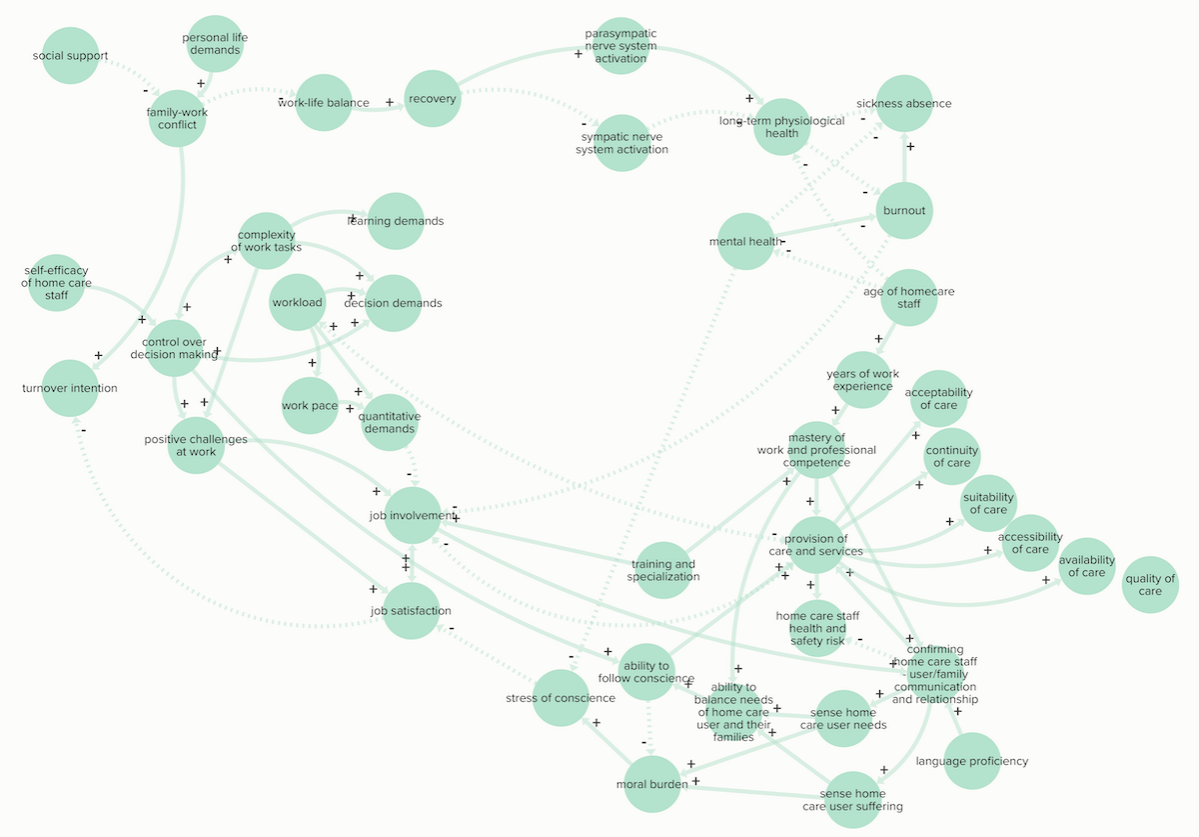
Elements and connections of the home care staff category of the final causal loop diagram. Arrows indicate the directional connections between elements. Positive and negative relationships are displayed in solid arrows (+) and dashed lines (–), respectively.

The home care staff category (Figure 4) included elements of the provision of care and services, primarily based on the quality satisfaction model developed by Samuelsson and Wister [56]. “Job demand” was dependent on 3 elements: learning, decision, and quantitative demands according to QPSNordic [111]. These depended on several elements, including “complexity of work tasks,” “workload,” and “work pace.”

Several elements of the home care staff category were connected to the “ability to cope” and subsequently “control” in the stress category. This included elements such as “job satisfaction and involvement,” “mastery of work,” “home care staff health and safety risk,” and “stress of conscience.”

Physiological and mental health responses to “distress” and their impact on home care staff burnout were described [58,59]. In this feedback loop “work-life balance” and “recovery” played an important role in balancing “distress” [58].

Multiple organizational elements are linked to the home care staff category. For instance, “role conflict” and “recognition” both affected “job satisfaction” [111]; “deskilling” affected “complexity of work tasks,” “training and specialization,” “ability to cope,” and “distress,” as well as “home care user trust in home care staff” [58,60-62]. Organizational elements related to working with technology (“working with sophisticated technology” and “equipment problems”) were linked to the home care staff category and were associated with an increase in “complexity of work tasks” and “workload” [60].

“Provision of care and services” was split into continuity, suitability, availability, influence, and personal relation as determining elements of the delivery of care and services in home care, quality, and home care user satisfaction with care. An increase in the home care user element “needs met” led to a reduction in “delivery of care and services,” and home care staff “stress of conscience” in 2 balancing feedback loops. In addition, the home care user’s ability to communicate, the staff “mastery of work and professional competence,” and “language proficiency” had a positive influence on “confirming home care staff-user relationship and family communication.” This element was an important determinant of “home care user influence” and “trust in home care staff” [119].

### Home Care User

**Figure 5:**
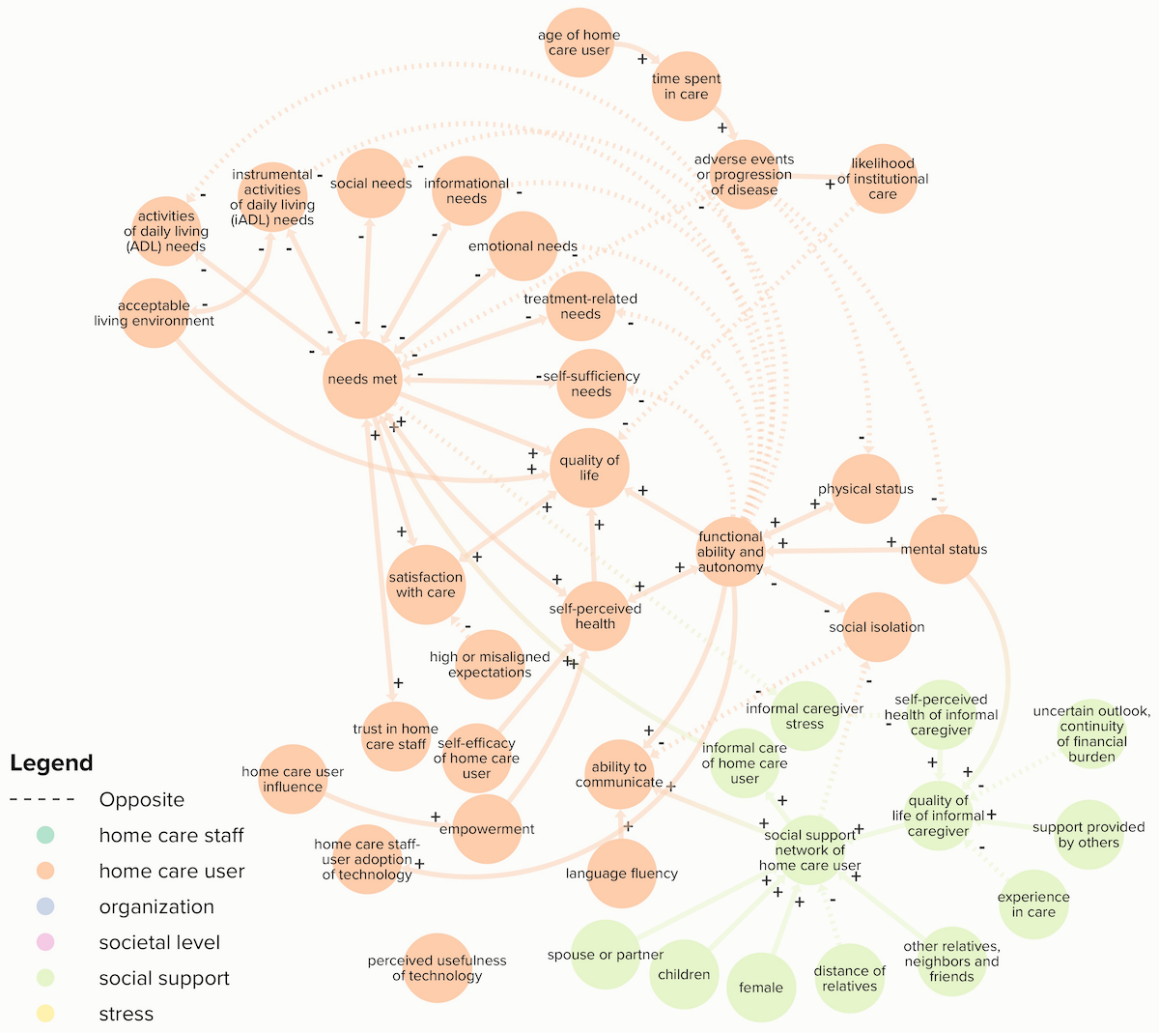
Elements and connections of the categories home care user and social support network (color-coded orange and green, respectively) of the final causal loop diagram. Arrows indicate the directional connections between elements. Positive and negative connections are displayed in solid arrows (+) and dashed lines (–), respectively.

The home care user category included individual needs, satisfaction with care, functional ability, and autonomy (Figure 5). Home care user “needs met” was determined by several elements related to instrumental and noninstrumental activities of daily living, social, emotional, informational, treatment-related, and self-sufficiency needs. This was mainly based on an analysis of home care user needs by Keeling [63]. Needs met influenced home care user satisfaction with care, self-perceived health, and quality of life [64].

A notable behavior of the home care user category related to the influence of “needs met” on adverse events or progression of the disease, where inadequate care led to an increase in the need for care in a reinforcing feedback loop. The emergence of “adverse events or progression of disease” due to unmet needs and “time spent in care” would then lead to a higher “likelihood of institutional care.” This would in turn affect “health care spending” in the societal level category.

### Organization

Many of the elements and connections of the category organization were based on the QPSNordic model [111]. This category (Figure 2) covered elements, such as the impact of leadership, social climate, and role clarity on workload, job involvement, and satisfaction. For instance, “quality of supervision” had a positive effect on “empowering leadership,” “human resource primacy,” and “commitment to the organization.” This in turn leads to greater “recognition,” home care staff “job satisfaction,” and “involvement.”

This category also described the impact of “organizational slack” and organizational “emphasis on cost-effectiveness” on the overall organization and working conditions [60,65-70,119]. For example, “emphasis on cost-effectiveness” led to “inadequate income” and “deskilling” of home care staff, which in turn reduced “job satisfaction,” “training and specialization,” as well as “mastery of work and professional competence,” hence having a negative impact on the “provision of care and services.”

### Social Support

The social support network of the home care user (Figure 5) influenced the “delivery of informal care,” therefore increasing home care user’s “needs met.” This was dependent on the “social support network of the home care user” and the “quality of life of the informal caregiver” [63,71]. Further, “needs met” reduced the “informal caregiver stress” and home care staff’s “stress of conscience” in addition to lowering the demand for the “provision of care and services” [63].

Therefore, if home care user “needs met” were fulfilled, this led to improved “quality of life of an informal caregiver,” enabling “informal care of home care user” in a reinforcing feedback loop.

### Societal Level

Figure 2 includes elements of the category societal level. The home care user element “likelihood of institutional care” affected the societal level element “health care spending” positively. Other elements in this category are related to the labor market and turnover intention. This included county-level unemployment, care capacity, capital income, and “stigmatization toward profession.” These elements all defined “job turnover” in the home care staff category [60,72].

### Qualitative Verification

Here is presented the verification against observed data from intervention studies related to organizational change in the nursing home setting (section S4 and Table S2 in Multimedia Appendix 1) and reablement in home care (section S4 and Table S3 in Multimedia Appendix 1). Scenarios from the peer-reviewed literature were compared with causal pathways of the CLD to assess agreement.

The final CLD largely supported the intervention-outcome combinations of the observed studies on a qualitative level. The final model showed improvement regarding consistency with the observed studies as compared to earlier iterations. No changes were seen at the final iterative stage. For example, Burgio and coworkers [26] studied the impact of nursing staff training for general communication skills, a motivational system, recognition of staff, and feedback on communication skills. This led to improved staff communication skills, positive staff statements, and a higher degree of independent self-care among residents. In the initial CLD, “training and specialization” led to improved “home care user and family communication,” and reduced “social needs” of the home care user. Also, an increase in “support from superior” led to staff “job satisfaction.” In the final CLD, “training and specialization” increased “confirming home care staff-user and family communication and relationship,” increasing the “functional ability and autonomy” of the home care user. Then, “support from superior” improved staff “job satisfaction.”

Most notable were the improvements in the model’s ability to capture intervention outcomes for the reablement approach (model element, “person-centered approach”). For example, Burton and colleagues [30] observed an improvement in home care user health-related quality of life and a reduction in home care needs following the introduction of a reablement approach [30]. The first version of the CLD was able to recover the impact of “functional ability” on “delivery of care.” The final model could describe the impact of the “person-centered approach” on the “provision of care and services” and home care users’ “quality of life.” The full list of comparator studies and related model pathways are given in Tables S2 and S3 in Multimedia Appendix 1.

### Simulations

A simulation analysis was performed to examine the theoretical impact of key elements of interest on the model (section S6 in Multimedia Appendix 1). The activation of “person-centered care” (Figure S7 in Multimedia Appendix 5) led to the activation of several elements related to delivery and quality of care and services, including “trust in home care staff,” “provision of care and services,” home care user “needs met,” and more. In addition, this led to a reduction in “home care staff health and safety risks.” On the other hand, the activation of the element “workload” led to a reduction in “provision of care and services,” “needs met,” and “job satisfaction.” Elements related to work demand, such as “quantitative demands,” “decision demands,” “work pace,” and “role conflict” increased. “Distress” remained unmodulated (see Discussion section). Simulating an activation of “home care staff-user adoption of technology” positively impacted factors related to “complexity of work tasks” along with “person-centered care.” Activation of “distress” led to a drop in elements related to home care staff health-related elements, as well as “job involvement” and “quality of care” (section S6 in Multimedia Appendix 1).

## Discussion

### Overview

This study presented the development, verification, and analysis of a CLD aiming to describe home care, including the impact of organizational change and reablement. The development process used participatory methods and qualitative verification to ensure fit-for-purpose.

### Model Analysis and Potential Impact

The model showed great potential for facilitating discussions of knowledge in the home care domain. The activities supported its use for informing improvement, the study design of intervention studies, and future quantitative modeling. For example, by using a systems approach, key elements, their connections, and accompanying indicators and instruments, can be mapped before the design of a study. The model could also serve as a basis for discussing organizational improvement and how to best plan care and services to meet demand while minimizing staff workload, considering the full complexity of the system. This is similarly to how CLDs have been used in health research to inform improvement and policymaking for health promotion, mental health, health systems, and combating antimicrobial resistance [18,22,25,120].

Important leverage points of the model were identified. These are potential targets for the design of interventions. For example, the model highlighted the importance of home care user “needs met” on both home care user, “adverse events or progression of disease,” and home care staff “provision of care and services” and “workload.” Meaning that this element is important for determining both home care staff’s “distress” and home care user “satisfaction with care,” “quality of life,” and “likelihood of institutional care.” Similarly to the job demands-resources model [116], home care staff “social support” indirectly had a positive effect on the “ability to cope” through a positive effect on “recovery,” therefore counteracting “distress.” This highlights the importance of taking a holistic systems perspective on interactions between elements when studying health care–related systems.

Focusing on the key elements of interest in this study, the behavior following activation of “person-centered care” (ie, the reablement approach) supported current evidence on the reablement approach and its influence on home care user needs met, functional ability and autonomy, and staff workload. The adoption of technology resulted in improved communication and coordination between services, but also an increased “complexity of work tasks” and “equipment problems.” Technology in the context of the reablement approach can lead to several benefits. However, care should be taken to ensure that this does not increase the workload of home care staff. Activation of “workload” and “distress” were important in determining the health of home care staff and the provision and quality of care.

### Limitations

The final model is subject to several limitations and assumptions and should not be taken as ground truth. Here, the participating expert group consisted of academic experts in health care sciences with a focus on home care and the reablement approach. To ensure the relevance of the model, it is of value to engage with stakeholders more broadly, including home care users, their relatives, home care staff, management, and policymakers. The model was also shaped by the user requirements and context under which it was developed. Here the main emphasis was on home care staff workload, stress, the impact of the reablement approach on the delivery of care and services, and other organizational factors. As multiple perspectives were explored during model development, the literature searches were not systematic reviews and should not be considered exhaustive, hence this introduces a potential source of bias. Due to the large size of the collected data set statistically significant and only quantitative relationships were considered for the model. Therefore, excluding potentially relevant effects. The model is grounded in the universal care system as relevant to Sweden. We can expect a higher relevance of the financial burden of provision of care and services on home care users and caring relatives in systems where home care is paid for by the users [121,122]. Hence, this work should not be viewed as a generic model of home care, although we believe it to be valuable for informing modeling work in other contexts.

Data on home care were supplemented with additional evidence from nursing home and residential care settings. To ensure model validity, the CLD was reviewed by the experts from the perspective of which setting evidence originated. The number of elements and relationships supported by evidence outside of the home care setting alone were minimal and still viewed to be of relevance to home care. Similarly, model verification was supplemented with intervention studies in the nursing home setting. Studies of interventions on staff communication skills, emotion-oriented care, staff de-escalation skills for aggressive behavior, and training on behavioral psychological symptoms for dementia may still be viewed as relevant for the home care setting [27]. While staff team building and supervisor training may be of less relevance due to the operational and organizational differences between home care and nursing homes [28,29]. Hence, this is an important consideration when interpreting the results. Nevertheless, as discussed above the difference between settings may vary between health systems and the overall results from the verification exercise (reablement studies in home care, intervention studies in nursing homes, and expert-based review) suggest the model be representative with regard to its aims. Future verification and validation will consider data originating from the home care setting in Sweden, based on the studies being carried out in the Future Care research program.

The qualitative simulations provided insight into the potential effect of modulating elements in the CLD. However, it should be noted that with equal weighting of all connections, this did not account for nonlinear effects such as the impact of demand and control on distress. This work can be further extended with more quantitative analysis methods. Using methods such as Boolean modeling or page rank, which were used in our previous work on a qualitative systems model of mental health [112], may be of value in further exploring the system behavior.

### Future Work

During development, reviewing the model during participatory activities served as an important medium for reflection and discussion on improvement and research in the domain of home care, work-related stress, and reablement. The model captured several aspects relating to the broader Future Care program. Going forward, the model may find use for study design as it encourages systems thinking when designing indicator sets for study protocols. This is further aided by the collated data where researchers can look up relevant instruments for measuring outcome variables across the diagram. The model may also serve as a basis for quantitative analysis of study data using structural equation modeling and perhaps even ordinary differential equation modeling in case of longitudinal data. Indeed, further work will focus on combining the qualitative system dynamics model with observed data on workload in home care to develop a quantitative predictive model.

### Conclusions

In this work we developed, verified, and analyzed a causal loop model of workload, work-related stress, delivery of care and services, and reablement in the home care setting. The model showed consistency across the comparator data set and may therefore be of value for informing improvement and intervention studies within the context of home care. Further work will focus on the wider inclusion of stakeholders in participatory activities to reduce the risk of bias. Translation into a quantitative model will also be explored using observed data.

## Appendix

Multimedia Appendix 1 Supplementary Material - Methods & Results.

Multimedia Appendix 2 Supplementary Table S4.

Multimedia Appendix 3 Causal loop diagram: connections between elements and their type (Table S5) and elements, category, and social network analysis metrics (Table S6).

Multimedia Appendix 4 Matlab simulation script.

Multimedia Appendix 5 Causal loop diagram figure.

## Abbreviations

CLD: causal loop diagram
ICT: information and communication technology
QPSnordic: the General Nordic Questionnaire for Psychological and Social Factors at Work
SDCS: Strain in Dementia Care Scale
