# Supplementary material for: Investigating the Connections Between Delivery of Care, Reablement, Workload, and Organizational Factors in Home Care Services: Mixed Methods Study
Source: JMIR Hum Factors. 2023 Jun 30;10:e42283. doi: 10.2196/42283 (PMC10365606; doi:10.2196/42283)
Supplement: Multimedia Appendix 1 [file humanfactors_v10i1e42283_app1.pdf]

Investigating the Connections between Delivery of Care, Reablement, Workload and Organisational Factors on Homecare Services: Mixed Methods Study - Supplementary Material – Methods & Results

Adam S. Darwich[1,*], Anne-Marie Boström[2,3,4], Susanne Guidetti[3,5,6], Jayanth Raghothama[1], Sebastiaan Meijer[1]

[1]Division of Health Informatics and Logistics, Department of Biomedical Engineering and Health Systems, KTH Royal Institute of Technology, Stockholm, Sweden.

[2]Department of Neurobiology, Care Science and Society, Division of Nursing, Karolinska Institutet, Huddinge, Sweden.

[3]Theme Inflammation and Aging, Karolinska University Hospital, Huddinge, Sweden.

[4]Research and Development Unit, Stockholms Sjukhem, Stockholm, Sweden.

[5]Department of Neurobiology, Care Sciences and Society, Division of Occupational Therapy, Karolinska Institutet, Stockholm, Sweden.

[6]Theme Women's Health and Allied Professionals, Medical Unit Occupational Therapy and Physiotherapy, Karolinska University Hospital.

**Communicating author:*

Adam S. Darwich,

KTH Royal Institute of Technology,

Division of Health Informatics and Logistics,

School of Engineering Sciences in Chemistry, Biotechnology and Health (CBH),

Hälsovägen 11C, 141 57 Huddinge, Sweden.

Tel: +46(0)8-790 48 05

Email: darwich@kth.se

Table of contents

S1. Model development supplementary 2
S2. Stress model 3
S3. Supplementary casual loop diagram 6
S4. Qualitative model verification 6
S5. Quantitative network analysis 12
S6. Model simulations 15

S1. Model development supplementary

Data collection was carried out using MEDLINE/PubMed (See Table S1 for full search term). This to identify quantitative and qualitative predictors of stress in homecare, residential care, care for older people, dementia care, nursing homes and related settings. Inclusion criteria were: qualitative and quantitative studies, literature reviews, studies including homecare users and/or staff, articles in English, published during 1990-2021. Case studies and studies of administrative staff in homecare were excluded.

A preliminary screening of titles and abstracts was carried out, followed by a review of the identified publications. Data was extracted relating to relationships between variables, study design, number and type of study participants, profession and setting, instrument or protocol used, country, statistically significant quantitative relationships between variables and statistical method. The data was compiled in MS Excel (Microsoft, Redwood, WA). The dataset is available in Table S4 of the Supplementary Material.

**Table S1.** The full search term of the first literature search.
(“home care”[Title/Abstract] OR “elderly care”[Title/Abstract] OR “dementia care”[Title/Abstract] OR “nursing home”[Title/Abstract] OR “residential care”[Title/Abstract]) AND (“job strain”[Title/Abstract] OR “work-related stress”[Title/Abstract] OR “workload”[Title/Abstract]) AND (“factors”[Title/Abstract] OR “variable”[Title/Abstract] OR “predictors”[Title/Abstract] OR “regressor”[Title/Abstract] OR “contributor”[Title/Abstract] OR “causal”[Title/Abstract] OR “systems dynamic”[Title/Abstract] OR “LISREL”[Title/Abstract] OR “SEM”[Title/Abstract] OR “structural equation model”[Title/Abstract] OR “relationship”[Title/Abstract] OR “relation”[Title/Abstract])

S2. Stress model

The implemented model of distress is detailed in Figure S1. In Figure S1A boredom/underload (B) and perceived workload (W) are both presented as time-dynamic states, where control will act to alleviate W and increase B. Demand will have the opposite effect, increasing W and reducing B. B and W both have a positive effect on, increasing, distress. Figure S1B depicts the same relationship using a simplified scheme of elements and positive/negative connections.

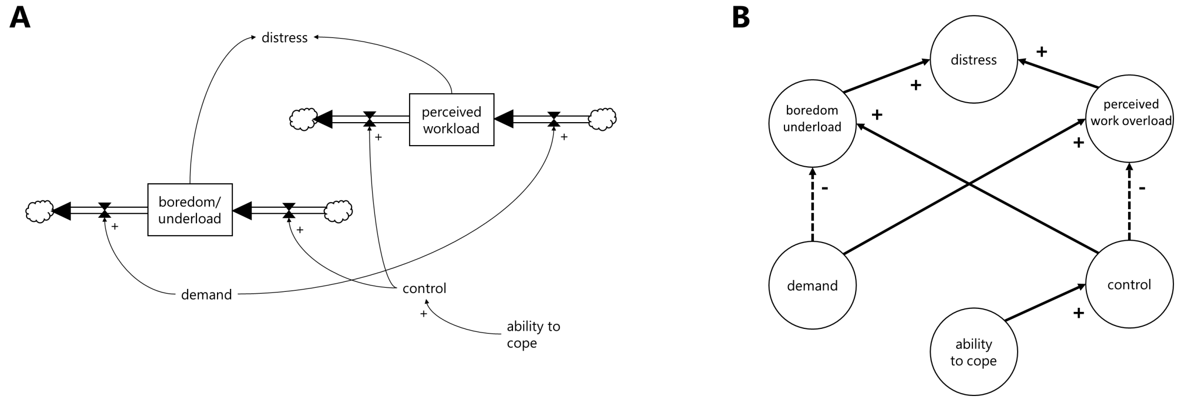

Figure S2. *Final stress model. A: system dynamics equation model. B: systems map.*

The behaviour of the simplified stress model was verified through theoretical simulations with arbitrarily set input parameters. Equation system 1 (Eq. 1) describes the ordinary differential equations of B and W along with their respective steady state solutions. As shown for B, the state is produced through an input of control (C) at a rate equal to C multiplied by a scalar (β_{BC}) and the rate, k_B. B is eliminated over time as a function of demand (D), the scalar β_{BD} and rate $k_{B,0}$. Similarly, W over time will depend on an input derived from D and output of C. B_{SS} and W_{SS} are the steady state solutions of the two states.

$$\frac{dB}{dt} = \beta_{BC} \cdot C \cdot k_B - B(t) \cdot \beta_{BD} \cdot D \cdot k_{B,0}, \quad B_{SS} = \frac{\beta_{BC} \cdot C \cdot k_B}{\beta_{BD} \cdot D \cdot k_{B,0}}$$

$$\frac{dW}{dt} = \beta_{WD} \cdot D \cdot k_W - W(t) \cdot \beta_{WC} \cdot C \cdot k_{W,0}, \quad W_{ss} = \frac{\beta_{WD} \cdot D \cdot k_W}{\beta_{WC} \cdot C \cdot k_{W,0}}$$

$$distress = B_{SS} + O_{ss}$$

Equation system (1)

Figure S2 shows the reference behaviour of the model when setting input parameters to arbitrarily defined values of 1. The U-shaped relationship is recovered between distress and demand/control. The stress model was further linked to physiological/mental health variables in order to relate distress to burnout [31].

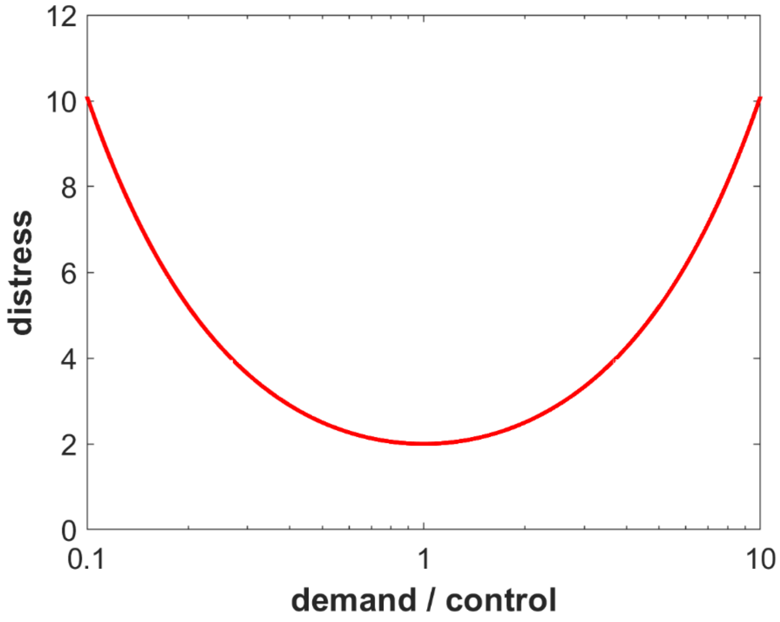

Figure S3. *Theoretical simulation of model behaviour derived from the final stress model. Note that values of distress and demand/control were set arbitrarily to examine the structural model behaviour.*

S3. Supplementary casual loop diagram

Figure S4 details the elements and connections of the causal loop diagram category organisation.

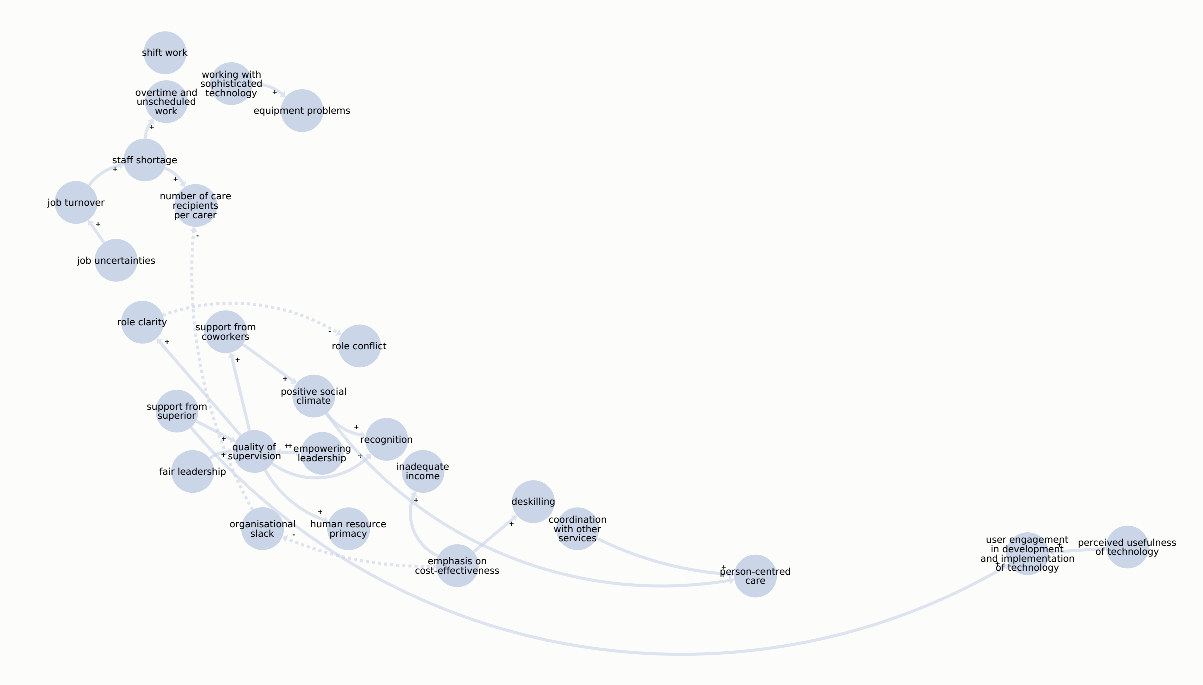

Figure S4. *Elements and connections of the category, organisation, of the final causal loop diagram. Arrows indicate the directional connections between elements. Positive and negative relationships are displayed in solid arrows (+) and dashed lines (-), respectively.*

S4. Qualitative model verification

An analysis was carried out of the study settings of the literature sources from which nodes and elements were derived, this was then reviewed by the experts. This as the literature review included studies from homecare, nursing homes and residential care. A vast majority of elements were supported by homecare-based research, with some elements originating from the nursing home/residential care literature. Aspects pertaining to the core stress model originated from the more general nursing enivornment, whereas pathophysiological apsects

of stress were informed by the medicinal literature. Information on self-efficacy of staff, number of care recipients per carer, labour market, sickness absence and organisational slack originated from the nursing home and residential care literature (Figure S5).

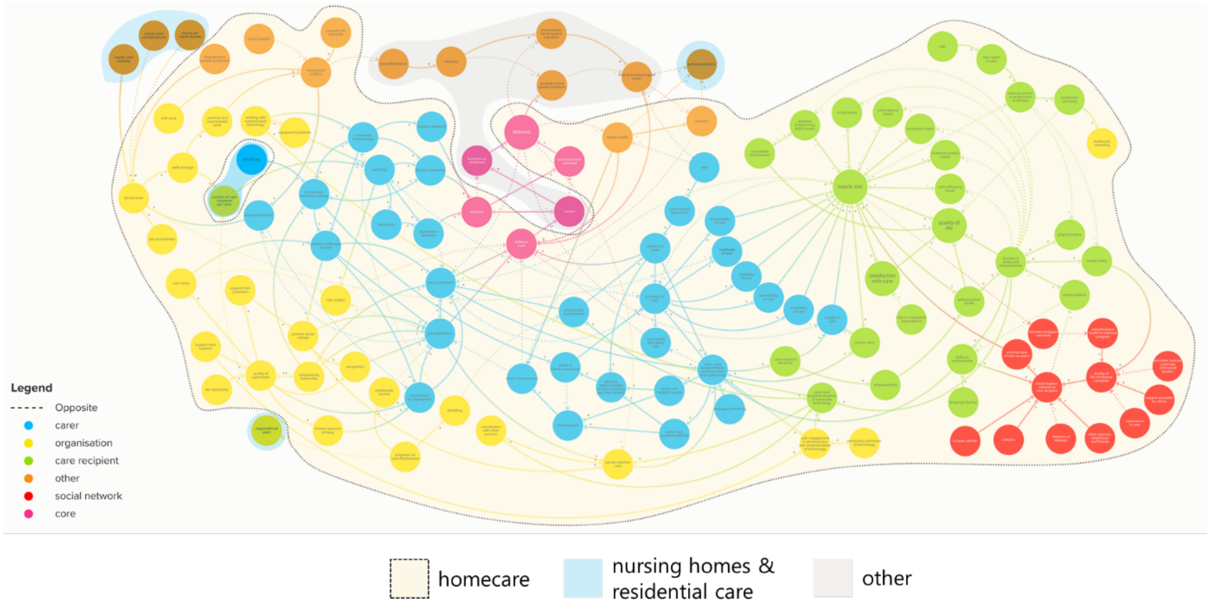

Figure S5. Iterative version of the causal loop model, detailing elements from literature specific to the homecare setting, nursing homes and residential care, and other.

Tables S2 and S3 details the qualitative verification through pathway analysis against observed intervention-outcomes in nursing homes and implementation of the reablement approach in homecare, respectively.

**Table S2.** Intervention-outcomes in nursing homes compared to shortest paths in the full causal loop model. Modified from Low *et al*. 2015 [27].					
**Intervention**	**Observed outcome(s)**	**CLD, final version: case pathways**	**CLD v. 2: case pathways**	**CLD v. 1: case pathways**	**Study reference**
• Nursing staff training in use of memory books and general communication skills.<br>• Motivational system, recognition of staff performance.<br>• Observation and feedback of staff communication skills.	• Improved staff communication skills.<br>• Positive staff statements.<br>• Residents more independent in self-care.	+ training and specialisation → + mastery of work → **+ confirming homecare staff-user/family communication and relationship** → + homecare user influence → + empowerment → self-perceived health **→ functional ability and autonomy**<br><br>+ support from superior → + quality supervision → + support from colleagues → + positive social climate → **+ job satisfaction**	+ training and specialisation → + mastery of work → **+ carer-care recipient/family communication and relationship** → + care recipient influence → + empowerment → self-perceived health → **functional ability and independence**<br><br>+ support from superior → + quality supervision → + support from colleagues → + positive social climate → **+ job satisfaction**	+ training and specialisation → + carer – care recipient/family communication → + delivery of care → + care recipient's needs met → - quantitative needs, - social needs.<br><br>+ support from superior → + quality supervision → + support from colleagues → + positive social climate → + job satisfaction.	[26]
• Staff training on emotion-oriented care.<br>• Supervision meetings.	• Some improvement in staff job satisfaction and burnout.	+ training and specialisation → + job involvement → + ability to cope → + control / demand → - distress → + mental health **→ - burnout**<br><br>+ support from superior → + quality supervision → + support from colleagues → + positive social climate → **+ job satisfaction**	+ training and specialisation → + job involvement → + ability to cope → + control / demand → - distress → + mental health → **- burnout**<br><br>+ support from superior → + quality supervision → + support from colleagues → + positive social climate → **+ job satisfaction**	+ training and specialisation → + job involvement → + ability to cope → + control / demand → - distress → + mental health → - burnout.<br><br>+ support from superior → + quality supervision → + support from colleagues → + positive social climate → + job satisfaction.	[32, 33]
• Staff training in restorative care, physical activity, positioning, mobility and transfers, communication,	• Improved staff goal attainment (evaluating complex needs of geriatric clients).	+training and specialisation → + mastery of work → + confirming homecare staff -	+training and specialisation → + mastery of work → + carer -care recipient/family communication	+ training and specialisation → + mastery of work → + carer – care recipient/family communication → +	[28]

eating, assessment and evaluation.<br>• Team building to motivate residents to reduce learned helplessness.	• Resident improved functional independence, self-care, progression and recovery in balance and mobility.	user/family communication and relationship → **+ sense homecare user needs**<br><br>+ empowerment → + self-perceived health → **+ functional ability and autonomy** → **+ physical status**	and relationship → **+ sense care recipient needs**<br><br>+ empowerment → + self-perceived health → **+ functional ability and independence** → **+ physical status**	sense consumer needs, + sense consumer suffering.<br><br>+ self-perceived health → + care recipient's needs met → - quantitative needs.	
• Staff training on fundamental de-escalation skills with residents exhibiting aggressive behaviour.	• Reduction in reported number of assaults.	+ training and specialisation → + mastery of work → + carer – confirming homecare staff-user /family communication and relationship → **- homecare staff health and safety risk**	+ training and specialisation → + mastery of work → + carer – care recipient/family communication and relationship → **- carer health and safety risk**	+ training and specialisation → + mastery of work → + carer – care recipient/family communication → - carer health and safety risk.	[34]
• Staff training for supervisors and care staff on dementia care and pain reduction.<br>• Supervisor training in leadership skills.	• Improvement in homecare staff-user communication and pain awareness.<br>• Work stress increased.<br>• Supervisory support received decreased for care staff and supervisors.	+training and specialisation → + mastery of work → + confirming homecare staff-user/family communication and relationship → **+ sense homecare staff needs** → + moral burden → + stress of conscience → - ability to cope → … → **+ distress**	+training and specialisation → + mastery of work → + carer -care recipient/family communication and relationship → **+ sense care recipient needs** → + moral burden → + stress of conscience → - ability to cope → … → **+ distress**	+ training and specialisation → + mastery of work → + carer – care recipient/family communication.<br><br>+ support from superior → + quality supervision → + support from colleagues → + positive social climate → + job satisfaction → + ability to cope.	[29]
• Training on behavioural psychological symptoms of dementia.<br>• Personalised staff consultation.	• Homecare users had lower agitation, aggression and behavioural disturbances.	+ training and specialisation → + mastery of work → + confirming homecare staff–user/family communication → **- homecare staff health and safety risk**	+ training and specialisation → + mastery of work → + carer – care recipient/family communication → **- carer health and safety risk**	+ training and specialisation → + mastery of work → + carer – care recipient/family communication → - carer health and safety risk.	[35]
CLD: causal loop diagram; v: version.					

**Table S3.** Reablement intervention studies and associated observed outcomes. Compared to shortest paths in the full model. Modified from Tessier *et al*. 2016 [12].				
**Observed outcome**	**CLD, final version: case pathways**	**CLD v. 2: case pathways**	**CLD v. 1: case pathways**	**Study reference**
• Improvement of health-related quality of life.<br>• Reduction in homecare needs	+ person-centred approach → + confirming homecare staff-user/family communication and relationship → + homecare user influence → + empowerment → + self-perceived health → + functional ability and autonomy → + needs met → **- provision of care and services & + quality of life**	+ person-centred approach → + carer-care recipient/family communication and relationship → + care recipient influence → + empowerment → + self-perceived health → + functional ability and independence → + needs met → **- provision of homecare & + quality of life**	+ functional ability → - quantitative needs, + self-perceived health → + care recipient's needs met → - delivery of care	[30]
• Reduction in need for services.	+ person-centred approach → + confirming homecare staff-user/family communication and relationship → + homecare user influence → + empowerment → + self-perceived health → + functional ability and autonomy → + needs met → **- provision of care and services**	+ person-centred approach → + carer-care recipient/family communication and relationship → + care recipient influence → + empowerment → + self-perceived health → + functional ability and independence → + needs met → **- provision of homecare**	+ functional ability → - quantitative needs, + self-perceived health → + care recipient's needs met → - delivery of care	[36]
• Increased job satisfaction.<br>• Reduced staff turnover.	+ person-centred approach → + confirming homecare staff-user/family communication and relationship → + homecare user influence → + empowerment → + self-perceived health → + functional ability and autonomy → + needs met → - provision of care and services → - workload → - quantitative demands → + job involvement → **+ job satisfaction** → - turnover intention → **- job turnover**	+ person-centred approach → + carer-care recipient/family communication and relationship → + care recipient influence → + empowerment → + self-perceived health → + functional ability and independence → + needs met → - provision of care → - workload → - quantitative demands → + job involvement → **+ job satisfaction** → - turnover intention → **- job turnover**	+ functional ability → - quantitative needs, + self-perceived health → + care recipient's needs met → - delivery of care → - workload → - role conflict → - job satisfaction → - turnover intention → job turnover	[37]
• Greater proportion of users needing fewer services	+ person-centred approach → + confirming homecare staff-user/family communication and relationship → +	+ person-centred approach → + carer-care recipient/family communication and relationship → + care recipient influence → + empowerment → + self-perceived	+ functional ability → - quantitative needs, + self-perceived health → + care recipient's needs met → - delivery of care	[38]

	homecare user influence → + empowerment → + self-perceived health → + functional ability and autonomy → **+ needs met**	health → + functional ability and independence → **+ needs met**		
• Lower probability of requiring further services.	+ person-centred approach → + confirming homecare staff-user/family communication and relationship → + homecare user influence → + empowerment → + self-perceived health → + functional ability and autonomy → + needs met → **- provision of care and services**	+ person-centred approach → + carer-care recipient/family communication and relationship → + care recipient influence → + empowerment → + self-perceived health → + functional ability and independence → + needs met → **- provision of homecare**	+ functional ability → - quantitative needs, + self-perceived health → + care recipient's needs met	[39]
• Lower probability of requiring further services.<br>• Lower cumulative costs.	+ person-centred approach → + confirming homecare staff-user/family communication and relationship → + homecare user influence → + empowerment → + self-perceived health → + functional ability and autonomy → + needs met → **- provision of care and services**	+ person-centred approach → + carer-care recipient/family communication and relationship → + care recipient influence → + empowerment → + self-perceived health → + functional ability and independence → + needs met → **- provision of homecare**	+ functional ability → - quantitative needs, + self-perceived health → + care recipient's needs met → - delivery of care	[40]
• Lower home service costs.	+ person-centred approach → + confirming homecare staff-user/family communication and relationship → + homecare user influence → + empowerment → + self-perceived health → + functional ability and autonomy → + needs met → **- provision of care and services**	+ person-centred approach → + carer-care recipient/family communication and relationship → + care recipient influence → + empowerment → + self-perceived health → + functional ability and independence → + needs met → **- provision of homecare**	+ functional ability → - quantitative needs, + self-perceived health → + care recipient's needs met → - delivery of care	[41, 42]
• Greater improvement in functional capacity compared to controls.<br>• Improvement in health-related quality of life.	+ person-centred approach → + confirming homecare staff-user/family communication and relationship → + homecare user influence → + empowerment → + self-perceived health → **+ functional ability and autonomy** → + needs met → **+ quality of life**	+ person-centred approach → + carer-care recipient/family communication and relationship → + care recipient influence → + empowerment → + self-perceived health → **+ functional ability and independence** → + needs met → **+ quality of life**	+ functional ability → - quantitative needs, + self-perceived health → + care recipient's needs met	[43, 44]

• Slower rate of decline in physical health of caregivers.	+ person-centred approach →+ confirming homecare staff-user/family communication and relationship → + homecare user influence → + empowerment → + self-perceived health → + functional ability and autonomy → + needs met → - provision of care and services → - workload → - job demand → … → + **long-term physiological health**	+ person-centred approach → + carer-care recipient/family communication and relationship → + care recipient influence → + empowerment → + self-perceived health → + functional ability and independence → + needs met → - provision of homecare → - workload → - demand → … → + **long-term physiological health**	+ functional ability → - quantitative needs, + self-perceived health → + care recipient's needs met → - delivery of care → - workload → - quantitative demands → - demand → + boredom, underload, - perceived work overload → - distress → + parasympatic nerve system activation, - sympatic nerve system activation → + long-term physiological health	[45]
• Greater improvement in homecare user functional capacity.<br>• Reduction in emergency department visits and length of homecare episode.	+ person-centred approach → + confirming homecare staff-user/family communication and relationship → + homecare user influence → + empowerment → + self-perceived health → **+ functional ability and autonomy** → + needs met → - adverse events or progression of disease → **- institutional care**	+ person-centred approach → + carer-care recipient/family communication and relationship → + care recipient influence → + empowerment → + self-perceived health → **+ functional ability and independence** → + needs met → - adverse events or progression of disease → **- institutional care**	+ functional ability	[46]
CLD: causal loop diagram; v: version.				

S5. Quantitative network analysis

Graph theory and social network analysis (SNA) have been applied to study structural relationships of causal loop models [55]. In this study, SNA metrics were calculated using the Kumu web platform to characterise the behaviour of the developed model by establishing what elements were the most central in the model and therefore important for regulation, or mediation of change. The analysis was carried out assuming equal weighting for all elements (referred to as nodes in SNA) and connections. Relevant centrality metrics included: betweenness – how frequently a node lies in the shortest path between other node pairs, closeness – shortest distance to all other nodes, degree – number of connections of a given node, eigenvector – considering the degree and importance of connected nodes, reach – the

proportion of nodes separated by two or less connections, and reach-efficiency – reach divided by the number of connected nodes [47-49].

Here follows the results of the SNA of the structure of the final causal loop model (Figure S6). The full results are available in Table S5 and S6. The elements, '*needs met*', '*provision of care and services*', '*job involvement*', 'c*onfirming homecare staff-user/family communication and relationship*' and '*job satisfaction*' showed the highest betweenness, meaning these elements are important bridges, alternatively bottlenecks, between other elements. '*Needs met*', '*functional ability and independence*', '*provision of care and services*', '*confirming..relationship*' and '*job involvement*' displayed the highest closeness and may therefore be considered important modulators for quickly affecting other elements of the network. For degree, '*needs met*', '*functional ability…*', '*provision of care and services*', '*confirming ... relationship*', and '*job satisfaction*' were the most prominent elements. These were the elements with the most connections to other elements in the network. '*Needs met*', '*quality of care*', '*satisfaction of care*' and '*provision of care and services*' displayed the highest eigenvector value, considering the degree and importance of connected elements. '*Needs met*', '*provision of care and services*' and '*functional ability and independence*' displayed the highest proportion of connected elements separated by two or less connections (reach). Although less prominent, '*accessibility of care*', and other related factors, displayed the highest reach-efficiency, or reach divided by the number of connections.

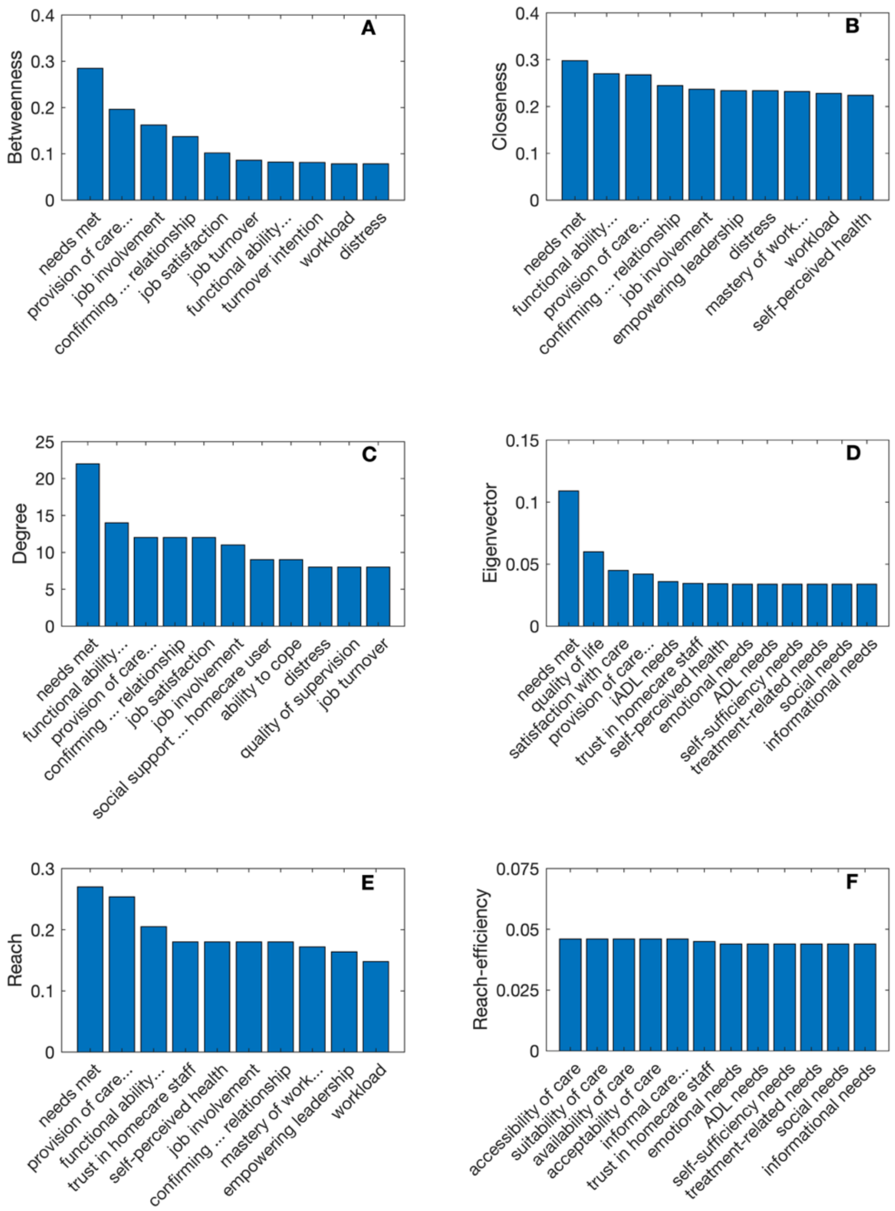

Figure S6. *Results from the social network analysis of the final causal loop model, showing metrics in relation to the top ten elements identified for respective metric - A: Betweenness; B: Closeness; C: Degree; D: Eigenvector-values; E: Reach; F: Reach-efficiency.*

S6. Model simulations

Here follows the full results from the simulation exercise. The implemented Matlab-script imported the Kumu model export file (in xlsx-format), extracted elements and connections, and allowed iterative activation of elements from a specified element of origin in the model. Connections were scaled at a prespecified coefficient. For the purpose of this analysis all elements were set to a baseline value of zero, the coefficients of the connections were set to an absolute coefficient of 0.7, +0.7 for all positive connections and -0.7 for all negative connections. Equal weighting was assumed for all connections. The simulations were carried out over ten iterations. The implemented simulation algorithm is available as a pdf-file in the Supplementary Material.

The impact of activating '*person-centred care*' (the reablement approach) is shown in the heatmap, Figure S7, and across iterations, Figure S8. Figure S9 and Figure S10 details the impact of activating the element '*workload*' on remaining elements of the causal loop diagram. Figure S11 and Figure S12 show the impact of activating '*homecare staff-user adoption of technology*' on remaining elements of the model. Finally, Figure S13 and Figure S14 provide the same information for the activation of the element '*distress*'.

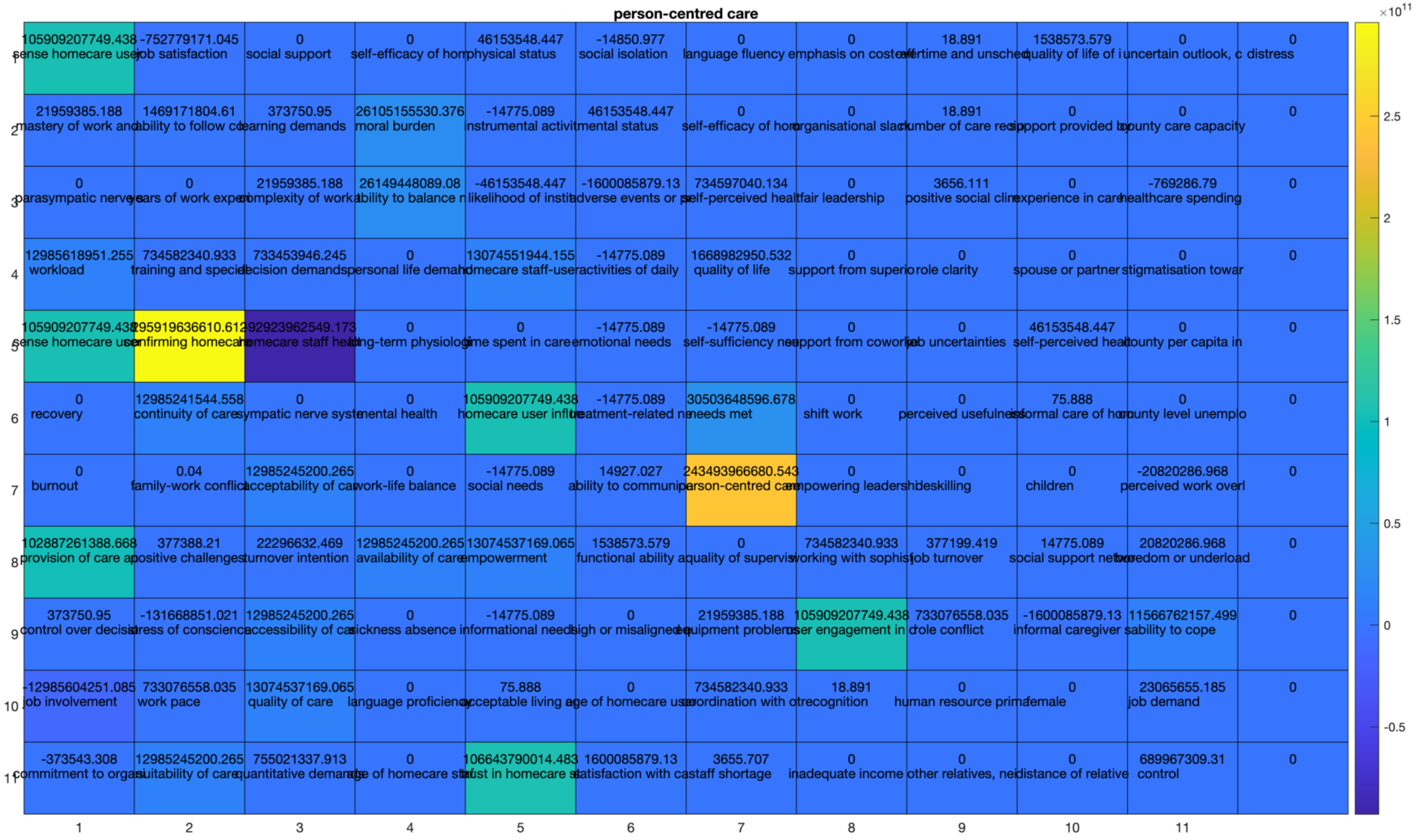

Figure S7. *Heatmap detailing element activation in the causal loop model when activating ‘ person-centred-care’ (i.e., the reablement approach).*

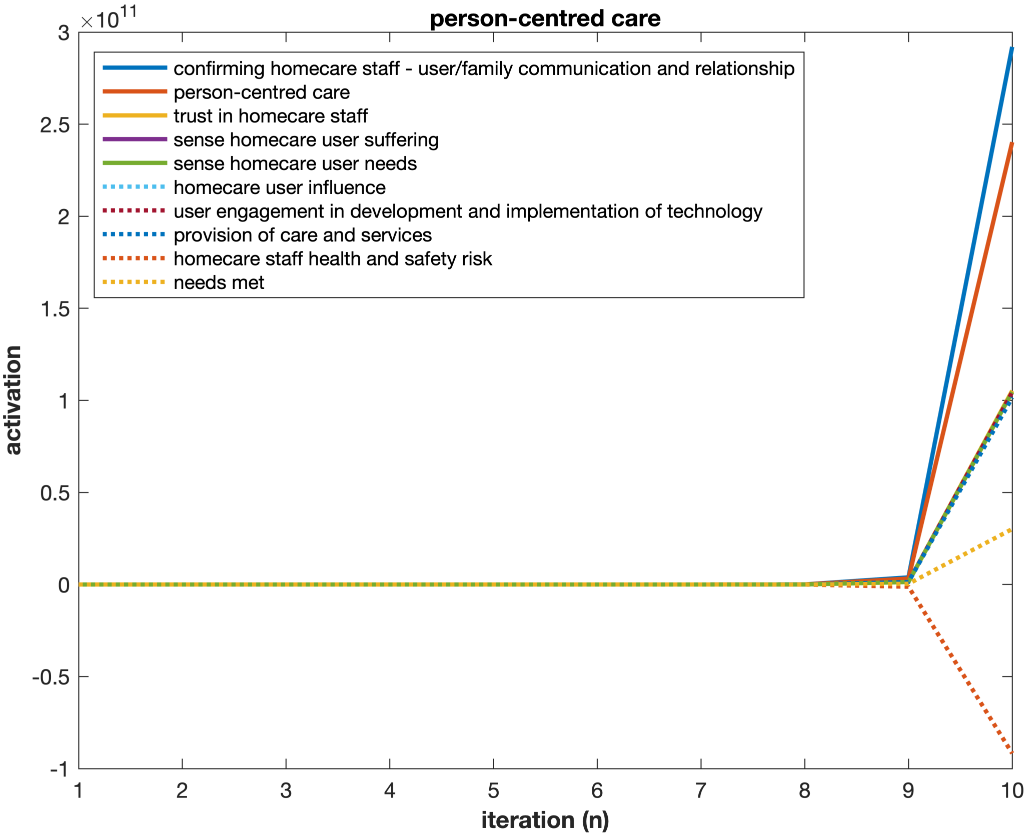

Figure S8. *Simulation results detailing the top ten activated elements when activating the element 'person-centred care' (the reablement approach) and simulating activation of connections across ten iterations.*

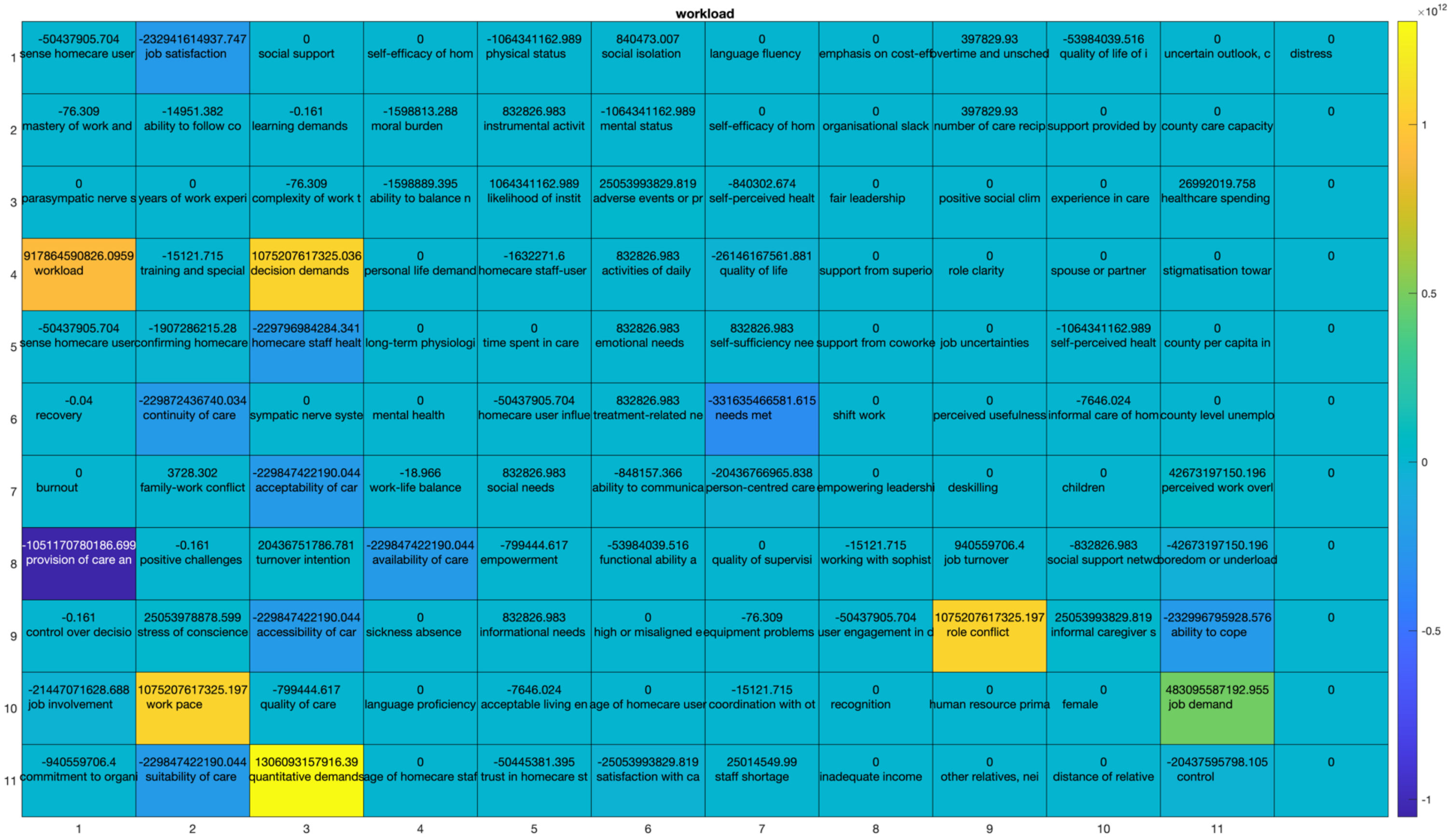

Figure S9. *Heatmap detailing element activation in the causal loop model when activating ‘ workload’ .*

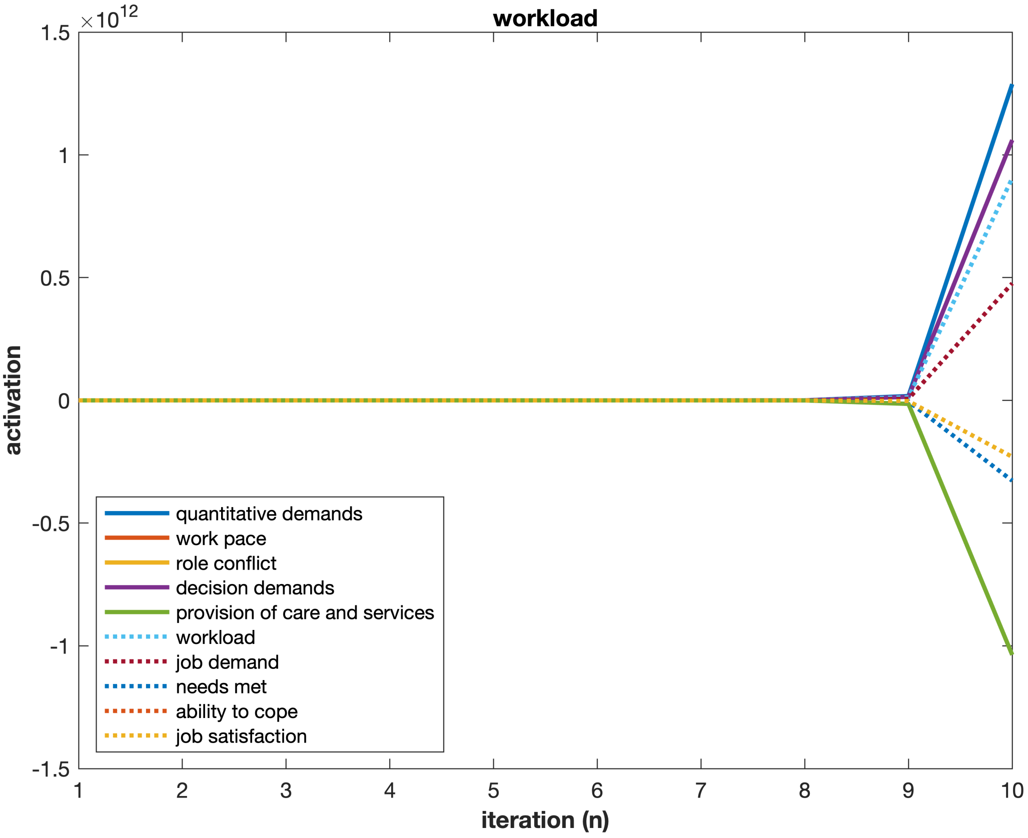

Figure S10. *Simulation results detailing the top ten activated elements when activating the element 'workload' and simulating activation of connections across ten iterations.*

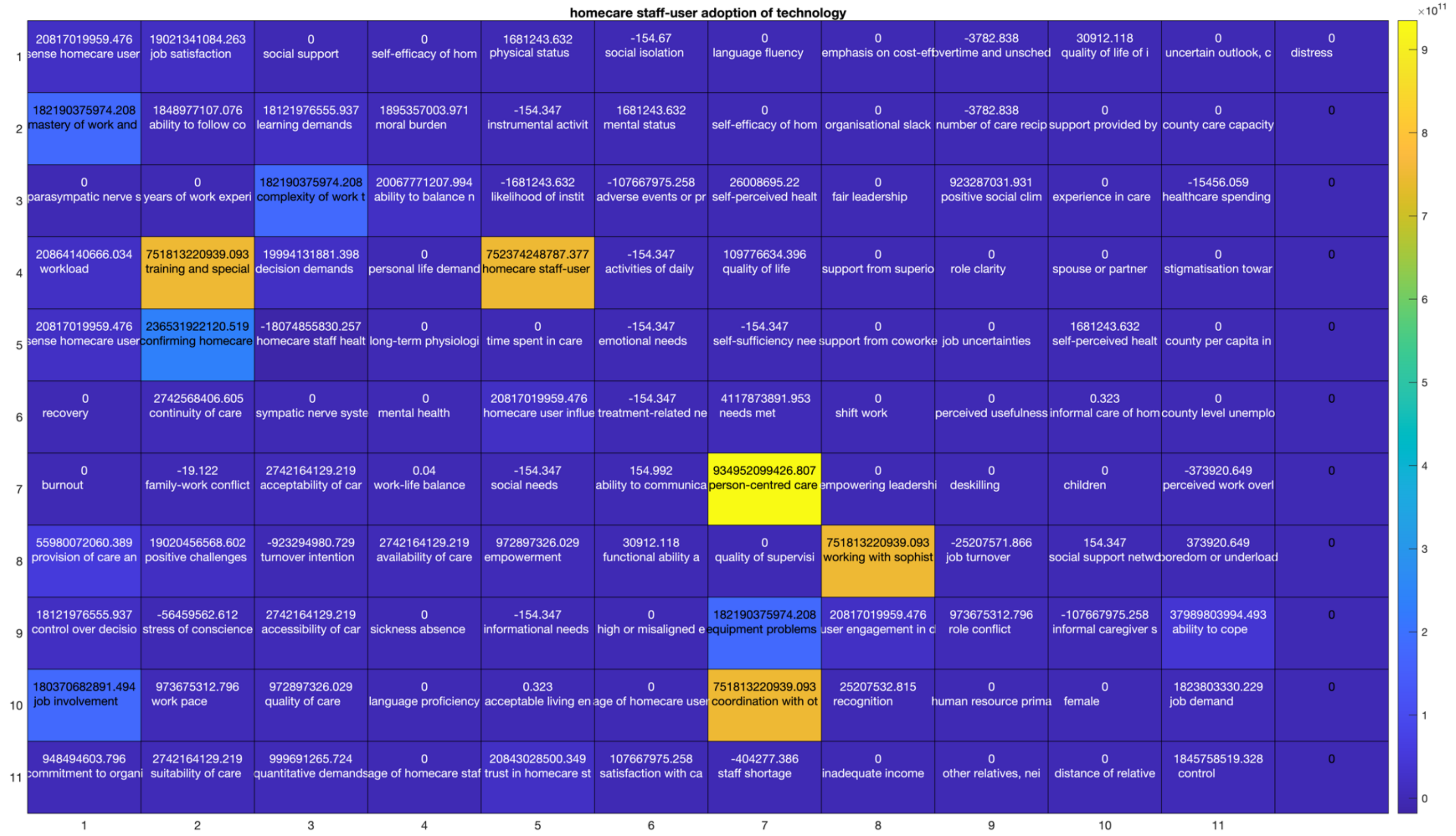

Figure S11. *Heatmap detailing element activation in the causal loop model when activating ‘ homecare staff-user adoption of technology’ .*

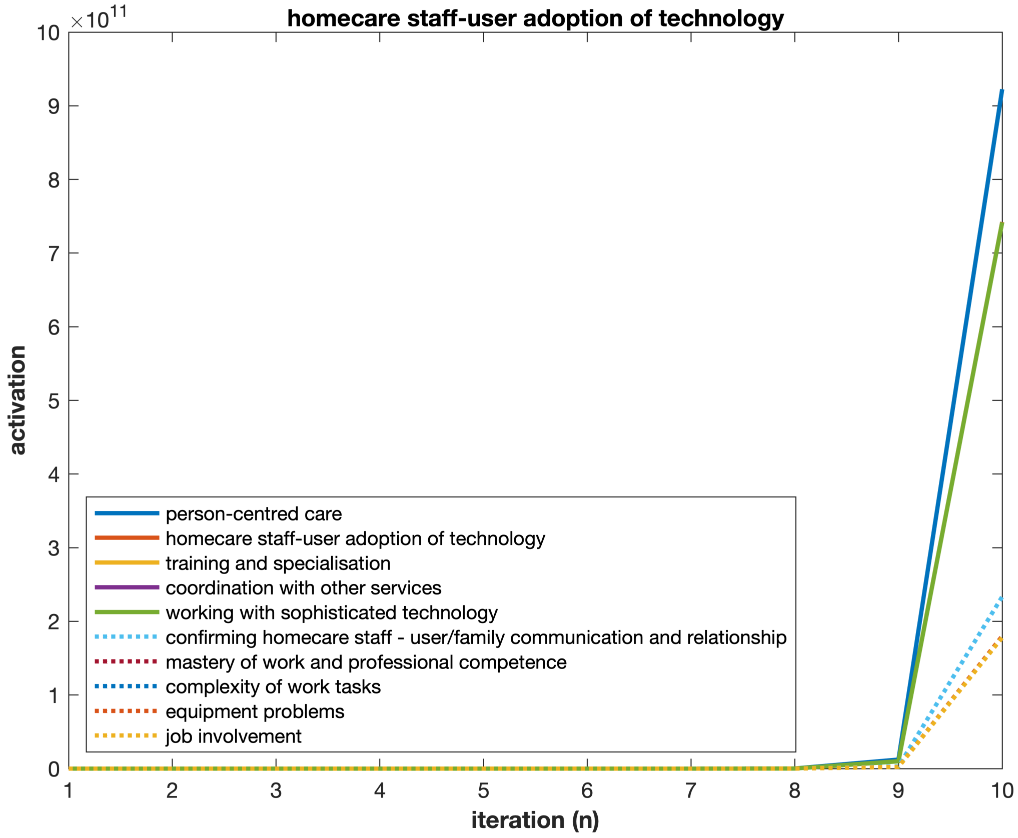

Figure S12. *Simulation results detailing the top ten activated elements when activating the element 'homecare staff-user adoption of technology' and simulating activation of connections across ten iterations.*

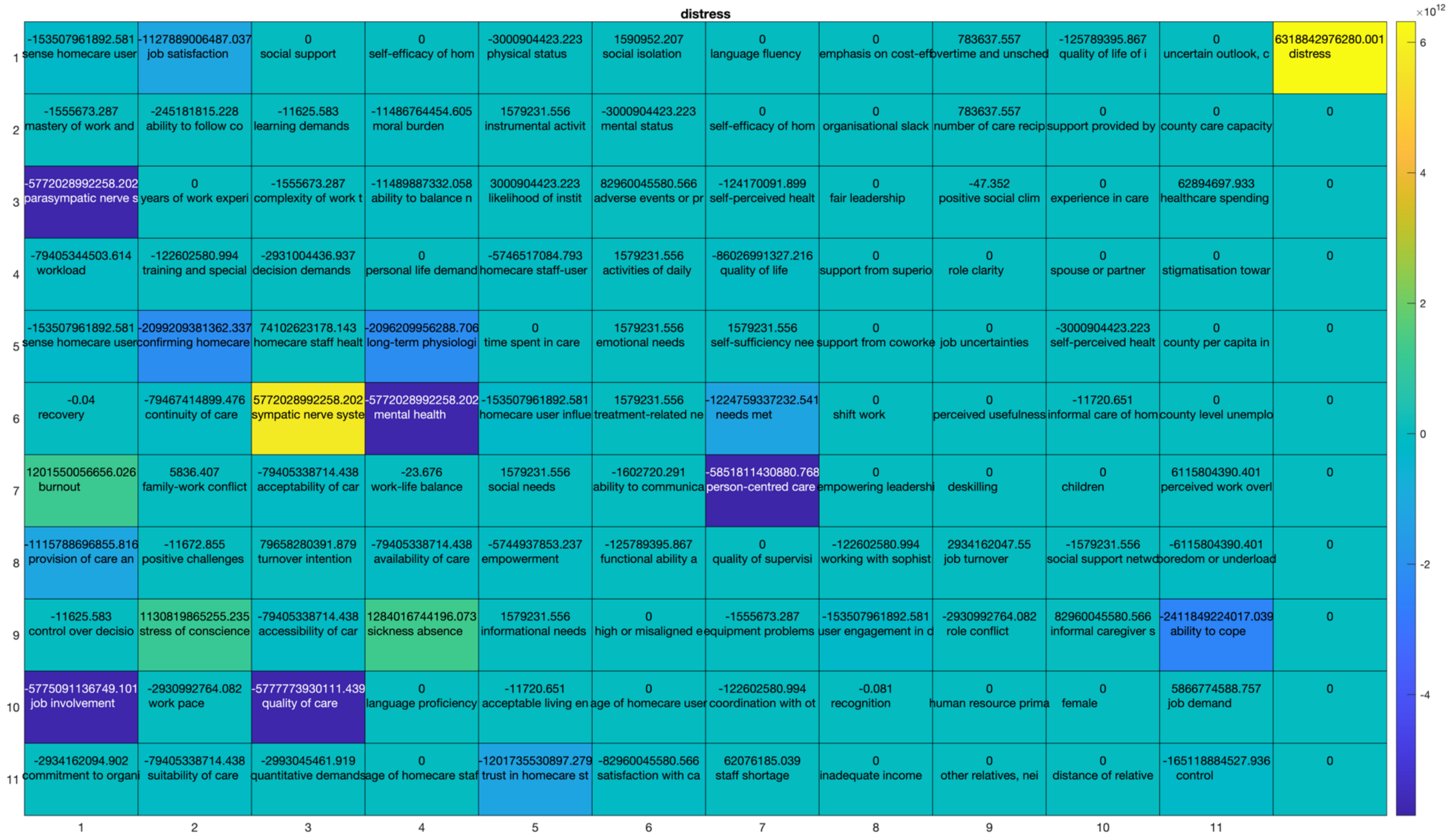

Figure S13. *Heatmap detailing element activation in the causal loop model when activating ‘ distress’ .*

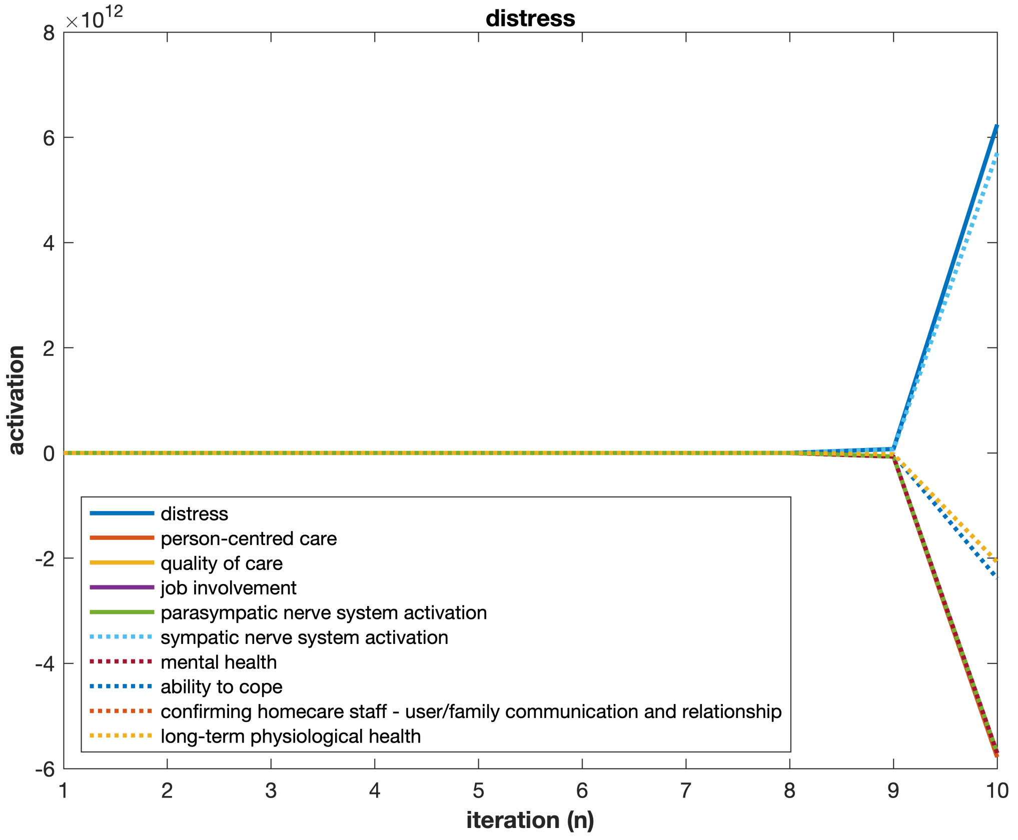

Figure S14. *Simulation results detailing the top ten activated elements when activating the element 'distress' and simulating activation of connections across ten iterations.*
