# Supplementary material for: Investigating the Connections Between Delivery of Care, Reablement, Workload, and Organizational Factors in Home Care Services: Mixed Methods Study
Source: JMIR Hum Factors. 2023 Jun 30;10:e42283. doi: 10.2196/42283 (PMC10365606; doi:10.2196/42283)
Supplement: Multimedia Appendix 2 [file humanfactors_v10i1e42283_app2.pdf]

**Table S4.** Supplementary Data.									
**Variable**	**Dependent variable**	**Strength of relation**	**Analysis method**	**Protocol or instrument**	**Participants**	**Country**	**Study method**	**Comment**	**Ref**
ward level: quality of leadership	perceived physical exertion	Adjusted beta: 0.016 (p=0.02)	multi-level linear mixed effects modelling	Psychosocial working conditions were measured by the Copenhagen Psychosocial Questionnaire (COPSOQ)	N=536 eldercare workers, nested in 126 wards and 20 nursing homes.	Denmark	Multi-level observational study, self-administered questionnaire	Data from the 'Danish Observational Study of Eldercare work and musculoskeletal disorderS' (DOSES).	[73]
ward level: quantitative demands	perceived physical exertion	Adjusted beta: 0.025 (p=0.03)	multi-level linear mixed effects modelling	Psychosocial working conditions were measured by the Copenhagen Psychosocial Questionnaire (COPSOQ)	N=536 eldercare workers, nested in 126 wards and 20 nursing homes.	Denmark	Multi-level observational study, self-administered questionnaire	Data from the 'Danish Observational Study of Eldercare work and musculoskeletal disorderS' (DOSES).	[73]
ward level: work pace	perceived physical exertion	Adjusted beta: 0.045 (p<0.001)	multi-level linear mixed effects modelling	Psychosocial working conditions were measured by the Copenhagen Psychosocial Questionnaire (COPSOQ)	N=536 eldercare workers, nested in 126 wards and 20 nursing homes.	Denmark	Multi-level observational study, self-administered questionnaire	Data from the 'Danish Observational Study of Eldercare work and musculoskeletal disorderS' (DOSES).	[73]
ward level: justice	perceived physical exertion	Adjusted beta: -0.002 (p=0.01)	multi-level linear mixed effects modelling	Psychosocial working conditions were measured by the Copenhagen Psychosocial Questionnaire (COPSOQ)	N=536 eldercare workers, nested in 126 wards and 20 nursing homes.	Denmark	Multi-level observational study, self-administered questionnaire	Data from the 'Danish Observational Study of Eldercare work and musculoskeletal disorderS' (DOSES).	[73]
nursing home level: influence	perceived physical exertion	Adjusted beta: -0.066 (p=0.01)	multi-level linear mixed effects modelling	Psychosocial working conditions were measured by the Copenhagen Psychosocial	N=536 eldercare workers, nested in 126 wards and 20 nursing homes.	Denmark	Multi-level observational study, self-administered questionnaire	Data from the 'Danish Observational Study of Eldercare work and musculoskeletal disorderS' (DOSES).	[73]

				Questionnaire (COPSOQ)					
nursing home level: quality of leadership	perceived physical exertion	Adjusted beta: -0.047 (p=0.02)	multi-level linear mixed effects modelling	Psychosocial working conditions were measured by the Copenhagen Psychosocial Questionnaire (COPSOQ)	N=536 eldercare workers, nested in 126 wards and 20 nursing homes.	Denmark	Multi-level observational study, self-administered questionnaire	Data from the 'Danish Observational Study of Eldercare work and musculoskeletal disorderS' (DOSES).	[73]
worker level: quantitative demands	perceived physical exertion	Adjusted beta: 0.025 (p<0.001)	multi-level linear mixed effects modelling	Psychosocial working conditions were measured by the Copenhagen Psychosocial Questionnaire (COPSOQ)	N=536 eldercare workers, nested in 126 wards and 20 nursing homes.	Denmark	Multi-level observational study, self-administered questionnaire	Data from the 'Danish Observational Study of Eldercare work and musculoskeletal disorderS' (DOSES).	[73]
worker level: work pace	perceived physical exertion	Adjusted beta: 0.026 (p<0.001)	multi-level linear mixed effects modelling	Psychosocial working conditions were measured by the Copenhagen Psychosocial Questionnaire (COPSOQ)	N=536 eldercare workers, nested in 126 wards and 20 nursing homes.	Denmark	Multi-level observational study, self-administered questionnaire	Data from the 'Danish Observational Study of Eldercare work and musculoskeletal disorderS' (DOSES).	[73]
worker level: emotional demands	perceived physical exertion	Adjusted beta: 0.017 (p=0.01)	multi-level linear mixed effects modelling	Psychosocial working conditions were measured by the Copenhagen Psychosocial Questionnaire (COPSOQ)	N=536 eldercare workers, nested in 126 wards and 20 nursing homes.	Denmark	Multi-level observational study, self-administered questionnaire	Data from the 'Danish Observational Study of Eldercare work and musculoskeletal disorderS' (DOSES).	[73]
worker level: influence	perceived physical exertion	Adjusted beta: -0.019 (p<0.001)	multi-level linear mixed effects modelling	Psychosocial working conditions were measured by the Copenhagen Psychosocial Questionnaire (COPSOQ)	N=536 eldercare workers, nested in 126 wards and 20 nursing homes.	Denmark	Multi-level observational study, self-administered questionnaire	Data from the 'Danish Observational Study of Eldercare work and musculoskeletal disorderS' (DOSES).	[73]

worker level: social support	perceived physical exertion	Adjusted beta: -0.013 (p=0.04)	multi-level linear mixed effects modelling	Psychosocial working conditions were measured by the Copenhagen Psychosocial Questionnaire (COPSOQ)	N=536 eldercare workers, nested in 126 wards and 20 nursing homes.	Denmark	Multi-level observational study, self-administered questionnaire	Data from the 'Danish Observational Study of Eldercare work and musculoskeletal disorderS' (DOSES).	[73]
worker level: quality of leadership	perceived physical exertion	Adjusted beta: -0.013 (p=0.05)	multi-level linear mixed effects modelling	Psychosocial working conditions were measured by the Copenhagen Psychosocial Questionnaire (COPSOQ)	N=536 eldercare workers, nested in 126 wards and 20 nursing homes.	Denmark	Multi-level observational study, self-administered questionnaire	Data from the 'Danish Observational Study of Eldercare work and musculoskeletal disorderS' (DOSES).	[73]
flat wages and lack of benefits	work-related stress	n/a	focused coding techniques	interview instruments informed by leading stress models.	agency executives' (n=20), home healthcare workers (n=25), home healthcare	USA	Qualitative inquiry		[60]
inadequate training	work-related stress	n/a	focused coding techniques	interview instruments informed by leading stress models.	agency executives' (n=20), home healthcare workers (n=25), home healthcare	USA	Qualitative inquiry		[60]
social isolation	work-related stress	n/a	focused coding techniques	interview instruments informed by leading stress models.	agency executives' (n=20), home healthcare workers (n=25), home healthcare	USA	Qualitative inquiry		[60]
discriminati on	work-related stress	n/a	focused coding techniques	interview instruments informed by leading stress models.	agency executives' (n=20), home healthcare workers (n=25), home healthcare	USA	Qualitative inquiry		[60]

psychosocial strains (patient/client family/death)	work-related stress	n/a	focused coding techniques	interview instruments informed by leading stress models.	agency executives' (n=20), home healthcare workers (n=25), home healthcare	USA	Qualitative inquiry		[60]
erractic schedules	financial strain/traffic/transportation/childcare	n/a	focused coding techniques	interview instruments informed by leading stress models.	agency executives' (n=20), home healthcare workers (n=25), home healthcare	USA	Qualitative inquiry		[60]
financial strain/traffic/transportation/childcare	work-related stress	n/a	focused coding techniques	interview instruments informed by leading stress models.	agency executives' (n=20), home healthcare workers (n=25), home healthcare	USA	Qualitative inquiry		[60]
dealing with difficult personalities and challenging conditions	work-related stress	n/a	focused coding techniques	interview instruments informed by leading stress models.	agency executives' (n=20), home healthcare workers (n=25), home healthcare	USA	Qualitative inquiry		[60]
building trust and create bonds	work-related stress	n/a	focused coding techniques	interview instruments informed by leading stress models.	agency executives' (n=20), home healthcare workers (n=25), home healthcare	USA	Qualitative inquiry		[60]
cope with their own grief over client loss and support client family	emotional stress	n/a	focused coding techniques	interview instruments informed by leading stress models.	agency executives' (n=20), home healthcare workers (n=25), home healthcare	USA	Qualitative inquiry		[60]

emotional stress	work-related stress	n/a	focused coding techniques	interview instruments informed by leading stress models.	agency executives' (n=20), home healthcare workers (n=25), home healthcare	USA	Qualitative inquiry		[60]
deskilling	work-related stress	n/a	focused coding techniques	interview instruments informed by leading stress models.	agency executives' (n=20), home healthcare workers (n=25), home healthcare	USA	Qualitative inquiry		[60]
poor interpersonal treatment of minorities	work-related stress	n/a	focused coding techniques	interview instruments informed by leading stress models.	agency executives' (n=20), home healthcare workers (n=25), home healthcare	USA	Qualitative inquiry		[60]
initial and on-going in-service training	work-related stress	n/a	focused coding techniques	interview instruments informed by leading stress models.	agency executives' (n=20), home healthcare workers (n=25), home healthcare	USA	Qualitative inquiry		[60]
lack of, or poor, communication	work-related stress	n/a	focused coding techniques	interview instruments informed by leading stress models.	agency executives' (n=20), home healthcare workers (n=25), home healthcare	USA	Qualitative inquiry		[60]
poor recruitment practices/unqualified staff	work-related stress	n/a	focused coding techniques	interview instruments informed by leading stress models.	agency executives' (n=20), home healthcare workers (n=25), home healthcare	USA	Qualitative inquiry		[60]

poor communication about changes	work-related stress	n/a	focused coding techniques	interview instruments informed by leading stress models.	agency executives' (n=20), home healthcare workers (n=25), home healthcare	USA	Qualitative inquiry		[60]
erratic schedules	work-related stress	n/a	focused coding techniques	interview instruments informed by leading stress models.	agency executives' (n=20), home healthcare workers (n=25), home healthcare	USA	Qualitative inquiry		[60]
social injustices in access to long-term care	work-related stress	n/a	focused coding techniques	interview instruments informed by leading stress models.	agency executives' (n=20), home healthcare workers (n=25), home healthcare	USA	Qualitative inquiry		[60]
cope with difficult patients	work-related stress	n/a	focused coding techniques	interview instruments informed by leading stress models.	agency executives' (n=20), home healthcare workers (n=25), home healthcare	USA	Qualitative inquiry		[60]
harsh workplace hierarchies	work-related stress	n/a	focused coding techniques	interview instruments informed by leading stress models.	agency executives' (n=20), home healthcare workers (n=25), home healthcare	USA	Qualitative inquiry		[60]
work ethics issues	work-related stress	n/a	focused coding techniques	interview instruments informed by leading stress models.	agency executives' (n=20), home healthcare workers (n=25), home healthcare	USA	Qualitative inquiry		[60]

difficulty to adopt new technologies e	work-related stress	n/a	focused coding techniques	interview instruments informed by leading stress models.	agency executives' (n=20), home healthcare workers (n=25), home healthcare	USA	Qualitative inquiry		[60]
lack of support at home	work-related stress	n/a	focused coding techniques	interview instruments informed by leading stress models.	agency executives' (n=20), home healthcare workers (n=25), home healthcare	USA	Qualitative inquiry		[60]
organisation al mergers	work-related stress	n/a	focused coding techniques	interview instruments informed by leading stress models.	agency executives' (n=20), home healthcare workers (n=25), home healthcare	USA	Qualitative inquiry		[60]
quality of leadership	work-related stress	n/a	focused coding techniques	interview instruments informed by leading stress models.	agency executives' (n=20), home healthcare workers (n=25), home healthcare	USA	Qualitative inquiry		[60]
support for workers	work-related stress	n/a	focused coding techniques	interview instruments informed by leading stress models.	agency executives' (n=20), home healthcare workers (n=25), home healthcare	USA	Qualitative inquiry		[60]
stigmatisatio n by internal staff, client/patient , general public	work-related stress	n/a	focused coding techniques	interview instruments informed by leading stress models.	agency executives' (n=20), home healthcare workers (n=25), home healthcare	USA	Qualitative inquiry		[60]

control costs/renumeration in organisation	low wages, deskilling	n/a	focused coding techniques	interview instruments informed by leading stress models.	agency executives' (n=20), home healthcare workers (n=25), home healthcare	USA	Qualitative inquiry		[60]
Limited language proficiency of patient	increased workload	n/a	qualitative secondary analysis using open coding techniques	semi-structured interview	25 home healthcare providers (registered nurses, physical therapists, occupational therapist), home healthcare	USA	Qualitative data analysis of semi-structured interview data		[74]
Limited language proficiency of patient	concern about patient safety	n/a	qualitative secondary analysis using open coding techniques	semi-structured interview	25 home healthcare providers (registered nurses, physical therapists, occupational therapist), home healthcare	USA	Qualitative data analysis of semi-structured interview data		[74]
Psychosocial climate	person-centred care	Beta: 0.349 ($p<0.001$)	multiple linear regression	Person-centred Climate Questionnaire-Staff version (PCQ-S): psychosocial climate; Satisfaction with Nursing Care and Work questionnaire (SNCW): satisfaction with nursing care and work; Swedish Demand-Control-Support Questionnaire (DCSQ): job strain;	N=1169 participants. Registered nurses, enrolled nurses, nurse's assistants, no formal/other education. Residential aged dementia care.	Sweden	Cross-sectional exploratory survey-based study		[75]

				Stress of Conscienc Questionnaire (SCQ): stress of conscience.					
Satisfaction with care and work	person-centred care	Beta: 0.135 (p<0.001)	multiple linear regression	Person-centred Climate Questionnaire-Staff version (PCQ-S): psychosocial climate; Satisfaction with Nursing Care and Work questionnaire (SNCW): satisfaction with nursing care and work; Swedish Demand-Control-Support Questionnaire (DCSQ): job strain; Stress of Conscienc Questionnaire (SCQ): stress of conscience.	N=1169 participants. Registered nurses, enrolled nurses, nurse's assistants, no formal/other education. Residential aged dementia care.	Sweden	Cross-sectional exploratory survey-based study		[75]
job strain	person-centred care	Beta: -3.641 (p=0.002)	multiple linear regression	Person-centred Climate Questionnaire-Staff version (PCQ-S): psychosocial climate; Satisfaction with Nursing Care and Work questionnaire (SNCW): satisfaction with nursing care and work; Swedish Demand-Control-Support Questionnaire	N=1169 participants. Registered nurses, enrolled nurses, nurse's assistants, no formal/other education. Residential aged dementia care.	Sweden	Cross-sectional exploratory survey-based study		[75]

				(DCSQ): job strain; Stress of Conscienc Questionnaire (SCQ): stress of conscience.					
Unit staffing level	perceived stress	Adjusted beta: -0.64 (p=0.015)	regression analysis	Psychosocial work factors: Job Content Questionnaire. Psychological distress: General Health Questionnaire (GHQ). Case-mix information: case-mix index (CMI) based on RUG-III-classification system.	N=1531 elderly care staff, elderly care setting	Finland	Cross-sectional survey study. Instrumental variable regression.		[76]
Job demand score (1-5)	perceived stress	Adjusted beta: 0.26 (p<0.001)	regression analysis	Psychosocial work factors: Job Content Questionnaire. Psychological distress: General Health Questionnaire (GHQ). Case-mix information: case-mix index (CMI) based on RUG-III-classification system.	N=1531 elderly care staff, elderly care setting	Finland	Cross-sectional survey study. Instrumental variable regression.		[76]
Job strain score (0.2-5.0)	perceived stress	Adjusted beta: 0.52 (p<0.001)	regression analysis	Psychosocial work factors: Job Content Questionnaire. Psychological distress: General Health Questionnaire (GHQ). Case-mix information: case-mix index (CMI) based on	N=1531 elderly care staff, elderly care setting	Finland	Cross-sectional survey study. Instrumental variable regression.		[76]

				RUG-III-classification system.					
Unit staffing level	Psychological distress	Adjusted beta: -0.29 (p=0.016)	regression analysis	Psychosocial work factors: Job Content Questionnaire. Psychological distress: General Health Questionnaire (GHQ). Case-mix information: case-mix index (CMI) based on RUG-III-classification system.	N=1531 elderly care staff, elderly care setting	Finland	Cross-sectional survey study. Instrumental variable regression.		[76]
Job demand score (1-5)	Psychological distress	Adjusted beta: 0.13 (p<0.001)	regression analysis	Psychosocial work factors: Job Content Questionnaire. Psychological distress: General Health Questionnaire (GHQ). Case-mix information: case-mix index (CMI) based on RUG-III-classification system.	N=1531 elderly care staff, elderly care setting	Finland	Cross-sectional survey study. Instrumental variable regression.		[76]
Job strain score (0.2-5.0)	Psychological distress	Adjusted beta: 0.29 (p<0.001)	regression analysis	Psychosocial work factors: Job Content Questionnaire. Psychological distress: General Health Questionnaire (GHQ). Case-mix information: case-mix index (CMI) based on RUG-III-classification system.	N=1531 elderly care staff, elderly care setting	Finland	Cross-sectional survey study. Instrumental variable regression.		[76]

Job demand score (1-5)	Sleeping problems	Adjusted beta: 0.18 (p<0.001)	regression analysis	Psychosocial work factors: Job Content Questionnaire. Psychological distress: General Health Questionnaire (GHQ). Case-mix information: case-mix index (CMI) based on RUG-III-classification system.	N=1531 elderly care staff, elderly care setting	Finland	Cross-sectional survey study. Instrumental variable regression.		[76]
Job strain score (0.2-5.0)	Sleeping problems	Adjusted beta: 0.49 (p<0.001)	regression analysis	Psychosocial work factors: Job Content Questionnaire. Psychological distress: General Health Questionnaire (GHQ). Case-mix information: case-mix index (CMI) based on RUG-III-classification system.	N=1531 elderly care staff, elderly care setting	Finland	Cross-sectional survey study. Instrumental variable regression.		[76]
Higher teamwork and safety climate	quality of care	OR: 6.19 (95% CI: 4.36-8.79)	multilevel logistic regression	Practice Environment Scale–Nurse Working Index	N=4311 care workers (registered nurses, licensed nurses, nurse aides), from 402 units in 155 nursing homes.	Switzerland	Cross-sectional study. Care worker reported quality of care.		[77]
Better staffing and resources adequacy	quality of care	OR: 2.94 (95% CI: 2.08-4.15)	multilevel logistic regression	Practice Environment Scale–Nurse Working Index	N=4311 care workers (registered nurses, licensed nurses, nurse aides), from 402 units in 155 nursing homes.	Switzerland	Cross-sectional study. Care worker reported quality of care.		[77]

Less stress due to workload	quality of care	OR: 0.71 (95% CI: 0.55-0.93)	multilevel logistic regression	Practice Environment Scale–Nurse Working Index	N=4311 care workers (registered nurses, licensed nurses, nurse aides), from 402 units in 155 nursing homes.	Switzerland	Cross-sectional study. Care worker reported quality of care.		[77]
Less implicit rationing of caring, rehabilitation, and monitoring	quality of care	OR: 0.34 (95% CI: 0.24-0.49)	multilevel logistic regression	Practice Environment Scale–Nurse Working Index	N=4311 care workers (registered nurses, licensed nurses, nurse aides), from 402 units in 155 nursing homes.	Switzerland	Cross-sectional study. Care worker reported quality of care.		[77]
less rationing of social care	quality of care	OR: 0.80 (95% CI: 0.69-0.92)	multilevel logistic regression	Practice Environment Scale–Nurse Working Index	N=4311 care workers (registered nurses, licensed nurses, nurse aides), from 402 units in 155 nursing homes.	Switzerland	Cross-sectional study. Care worker reported quality of care.		[77]
Physical workload	intention to leave	Beta: 0.068 (p<0.001)	Anova	Musculoskeletal Intervention Center (MUSIC), Generalized Self-Efficacy Assessment.	N=6929 employees in elderly care (Nurses, other health-related education, no health-related education), elderly care.	Denmark	Cross-sectional questionnaire based study		[78]
Collective efficacy*physical workload	intention to leave	Beta: -0.009 (p<0.001)	Anova	Musculoskeletal Intervention Center (MUSIC), Generalized Self-Efficacy Assessment.	N=6929 employees in elderly care (Nurses, other health-related education, no health-related	Denmark	Cross-sectional questionnaire based study		[78]

					education), elderly care.				
Physical workload	sickness absence	OR: 1.16 (95% CI: 1.11-1.22; p<0.001)	Multinomial logistic regression	Musculoskeletal Intervention Center (MUSIC), Generalized Self-Efficacy Assessment.	N=6929 employees in elderly care (Nurses, other health-related education, no health-related education), elderly care.	Denmark	Cross-sectional questionnaire based study		[78]
Collective efficacy*physical workload	sickness absence	OR: 1.00, 0.99-1.01 (NS, p=0.99)	Multinomial logistic regression	Musculoskeletal Intervention Center (MUSIC), Generalized Self-Efficacy Assessment.	N=6929 employees in elderly care (Nurses, other health-related education, no health-related education), elderly care.	Denmark	Cross-sectional questionnaire based study		[78]
Location of facility - urban vs. rural	times felt rushed	Effect size: 0.190 (p<0.01)	Spearman correlation	SF-8 Health Survey, Alberta Context Tool (ACT).	N=583 health care aides, nursing home setting	Canada	Longitudinal cross-sectional survey study, TREC survey		[66]
Location of facility - urban vs. rural	number of activities missed due to lack of time	Effect size: 0.145 (p<0.01)	Spearman correlation	SF-8 Health Survey, Alberta Context Tool (ACT).	N=583 health care aides, nursing home setting	Canada	Longitudinal cross-sectional survey study, TREC survey		[66]
Size of facility - small (35-79 beds), medium (80-120 beds),	times felt rushed	Effect size: -0.269 (p<0.01)	Spearman correlation	SF-8 Health Survey, Alberta Context Tool (ACT).	N=583 health care aides, nursing home setting	Canada	Longitudinal cross-sectional survey study, TREC survey		[66]

large (>120 beds)									
Size of facility - small (35-79 beds), medium (80-120 beds), large (>120 beds)	number of activities missed due to lack of time	Effect size: -0.300 (p<0.01)	Spearman correlation	SF-8 Health Survey, Alberta Context Tool (ACT).	N=583 health care aides, nursing home setting	Canada	Longitudinal cross-sectional survey study, TREC survey		[66]
organisational context - lower vs. higher context	times felt rushed	Effect size: 0.291 (p<0.01)	Spearman correlation	SF-8 Health Survey, Alberta Context Tool (ACT).	N=583 health care aides, nursing home setting	Canada	Longitudinal cross-sectional survey study, TREC survey		[66]
organisational context - lower vs. higher context	number of activities missed due to lack of time	Effect size: 0.341 (p<0.01)	Spearman correlation	SF-8 Health Survey, Alberta Context Tool (ACT).	N=583 health care aides, nursing home setting	Canada	Longitudinal cross-sectional survey study, TREC survey		[66]
Work demand - my job requires working very fast	Job strain	p<0.001	Students t-test	Self-report demand and control questionnaire.	N=344 nursing staff, residential dementia care	Sweden	Intervention study combined with survey method		[79]
Work demand - my job requires working very hard	Job strain	p<0.001	Students t-test	Self-report demand and control questionnaire.	N=344 nursing staff, residential dementia care	Sweden	Intervention study combined with survey method		[79]
Work demand - my job requires a too high work effort	Job strain	p<0.001	Students t-test	Self-report demand and control questionnaire.	N=344 nursing staff, residential dementia care	Sweden	Intervention study combined with survey method		[79]

Work demand - I have enough time to accomplish the work	Job strain	p<0.001	Students t-test	Self-report demand and control questionnaire.	N=344 nursing staff, residential dementia care	Sweden	Intervention study combined with survey method		[79]
Work demand - There are often conflicting demands in my work	Job strain	p<0.001	Students t-test	Self-report demand and control questionnaire.	N=344 nursing staff, residential dementia care	Sweden	Intervention study combined with survey method		[79]
Work control - My job requires that I learn new things	Job strain	p<0.001	Students t-test	Self-report demand and control questionnaire.	N=344 nursing staff, residential dementia care	Sweden	Intervention study combined with survey method		[79]
Work control - I have the freedom to decide how to accomplish my work	Job strain	p<0.001	Students t-test	Self-report demand and control questionnaire.	N=344 nursing staff, residential dementia care	Sweden	Intervention study combined with survey method		[79]
Work control - I have the freedom to decide what to accomplish my work	Job strain	p<0.001	Students t-test	Self-report demand and control questionnaire.	N=344 nursing staff, residential dementia care	Sweden	Intervention study combined with survey method		[79]
Climate	Job strain	Beta: -0.003, p<0.001	Standard multiple regression	Self-report demand and control questionnaire.	N=344 nursing staff, residential dementia care	Sweden	Intervention study combined with survey method		[79]

Education	Job strain	Beta: -0.088, p=0.009	Standard multiple regression	Self-report demand and control questionnaire.	N=344 nursing staff, residential dementia care	Sweden	Intervention study combined with survey method		[79]
Ethics	Job strain	Beta: -0.163, p=0.03	Standard multiple regression	Self-report demand and control questionnaire.	N=344 nursing staff, residential dementia care	Sweden	Intervention study combined with survey method		[79]
Age: 31-40 yrs	back pain (physical health outcomes)	p<0.05	multiple regression models	Care worker Personnel Questionnaire of the SHURP study.	N=3471 care workers, in 155 nursing homes.	Switzerla nd	Secondary data analysis of cross-sectional survey study.		[80]
Age: 41-50 yrs	back pain (physical health outcomes)	p<0.05	multiple regression models	Care worker Personnel Questionnaire of the SHURP study.	N=3471 care workers, in 155 nursing homes.	Switzerla nd	Secondary data analysis of cross-sectional survey study.		[80]
Age: >50 yrs	back pain (physical health outcomes)	p<0.05	multiple regression models	Care worker Personnel Questionnaire of the SHURP study.	N=3471 care workers, in 155 nursing homes.	Switzerla nd	Secondary data analysis of cross-sectional survey study.		[80]
Age: >50 yrs	joint pain (physical health outcomes)	p<0.05	multiple regression models	Care worker Personnel Questionnaire of the SHURP study.	N=3471 care workers, in 155 nursing homes.	Switzerla nd	Secondary data analysis of cross-sectional survey study.		[80]
Work stressors: workload	back pain (physical health outcomes)	p<0.05	multiple regression models	Care worker Personnel Questionnaire of the SHURP study.	N=3471 care workers, in 155 nursing homes.	Switzerla nd	Secondary data analysis of cross-sectional survey study.		[80]
Work stressors: lack of job preparation	back pain (physical health outcomes)	p<0.05	multiple regression models	Care worker Personnel Questionnaire of the SHURP study.	N=3471 care workers, in 155 nursing homes.	Switzerla nd	Secondary data analysis of cross-sectional survey study.		[80]

Work stressors: conflict and lack of recognition	back pain (physical health outcomes)	p<0.05	multiple regression models	Care worker Personnel Questionnaire of the SHURP study.	N=3471 care workers, in 155 nursing homes.	Switzerland	Secondary data analysis of cross-sectional survey study.		[80]
Staffing adequacy	back pain (physical health outcomes)	p<0.05	multiple regression models	Care worker Personnel Questionnaire of the SHURP study.	N=3471 care workers, in 155 nursing homes.	Switzerland	Secondary data analysis of cross-sectional survey study.		[80]
Workplace violence toward care worker: verbal aggression	back pain (physical health outcomes)	p<0.05	multiple regression models	Care worker Personnel Questionnaire of the SHURP study.	N=3471 care workers, in 155 nursing homes.	Switzerland	Secondary data analysis of cross-sectional survey study.		[80]
Work stressors: workload	joint pain (physical health outcomes)	p<0.05	multiple regression models	Care worker Personnel Questionnaire of the SHURP study.	N=3471 care workers, in 155 nursing homes.	Switzerland	Secondary data analysis of cross-sectional survey study.		[80]
Work stressors: lack of job preparation	joint pain (physical health outcomes)	p<0.05	multiple regression models	Care worker Personnel Questionnaire of the SHURP study.	N=3471 care workers, in 155 nursing homes.	Switzerland	Secondary data analysis of cross-sectional survey study.		[80]
Work stressors: conflict and lack of recognition	joint pain (physical health outcomes)	p<0.05	multiple regression models	Care worker Personnel Questionnaire of the SHURP study.	N=3471 care workers, in 155 nursing homes.	Switzerland	Secondary data analysis of cross-sectional survey study.		[80]
Staffing adequacy	joint pain (physical health outcomes)	p<0.05	multiple regression models	Care worker Personnel Questionnaire of the SHURP study.	N=3471 care workers, in 155 nursing homes.	Switzerland	Secondary data analysis of cross-sectional survey study.		[80]

Workplace violence toward care worker: verbal aggression	joint pain (physical health outcomes)	p<0.05	multiple regression models	Care worker Personnel Questionnaire of the SHURP study.	N=3471 care workers, in 155 nursing homes.	Switzerland	Secondary data analysis of cross-sectional survey study.		[80]
Age: >50 yrs	sleeplessness (mental health outcomes)	p<0.05	multiple regression models	Care worker Personnel Questionnaire of the SHURP study.	N=3471 care workers, in 155 nursing homes.	Switzerland	Secondary data analysis of cross-sectional survey study.		[80]
Work stressors: workload	sleeplessness (mental health outcomes)	p<0.05	multiple regression models	Care worker Personnel Questionnaire of the SHURP study.	N=3471 care workers, in 155 nursing homes.	Switzerland	Secondary data analysis of cross-sectional survey study.		[80]
Work stressors: conflict and lack of recognition	sleeplessness (mental health outcomes)	p<0.05	multiple regression models	Care worker Personnel Questionnaire of the SHURP study.	N=3471 care workers, in 155 nursing homes.	Switzerland	Secondary data analysis of cross-sectional survey study.		[80]
Age: 41-50 yrs	tiredness (mental health outcomes)	p<0.05	multiple regression models	Care worker Personnel Questionnaire of the SHURP study.	N=3471 care workers, in 155 nursing homes.	Switzerland	Secondary data analysis of cross-sectional survey study.		[80]
Age: >50 yrs	tiredness (mental health outcomes)	p<0.05	multiple regression models	Care worker Personnel Questionnaire of the SHURP study.	N=3471 care workers, in 155 nursing homes.	Switzerland	Secondary data analysis of cross-sectional survey study.		[80]
Work stressors: workload	tiredness (mental health outcomes)	p<0.05	multiple regression models	Care worker Personnel Questionnaire of the SHURP study.	N=3471 care workers, in 155 nursing homes.	Switzerland	Secondary data analysis of cross-sectional survey study.		[80]

Work stressors: conflict and lack of recognition	tiredness (mental health outcomes)	p<0.05	multiple regression models	Care worker Personnel Questionnaire of the SHURP study.	N=3471 care workers, in 155 nursing homes.	Switzerland	Secondary data analysis of cross-sectional survey study.		[80]
Staffing adequacy	tiredness (mental health outcomes)	p<0.05	multiple regression models	Care worker Personnel Questionnaire of the SHURP study.	N=3471 care workers, in 155 nursing homes.	Switzerland	Secondary data analysis of cross-sectional survey study.		[80]
Age: 41-50 yrs	headache (mental health outcomes)	p<0.05	multiple regression models	Care worker Personnel Questionnaire of the SHURP study.	N=3471 care workers, in 155 nursing homes.	Switzerland	Secondary data analysis of cross-sectional survey study.		[80]
Age: >50 yrs	headache (mental health outcomes)	p<0.05	multiple regression models	Care worker Personnel Questionnaire of the SHURP study.	N=3471 care workers, in 155 nursing homes.	Switzerland	Secondary data analysis of cross-sectional survey study.		[80]
Work stressors: workload	headache (mental health outcomes)	p<0.05	multiple regression models	Care worker Personnel Questionnaire of the SHURP study.	N=3471 care workers, in 155 nursing homes.	Switzerland	Secondary data analysis of cross-sectional survey study.		[80]
Work stressors: conflict and lack of recognition	headache (mental health outcomes)	p<0.05	multiple regression models	Care worker Personnel Questionnaire of the SHURP study.	N=3471 care workers, in 155 nursing homes.	Switzerland	Secondary data analysis of cross-sectional survey study.		[80]
Collaboration with: Nursing director	headache (mental health outcomes)	p<0.05	multiple regression models	Care worker Personnel Questionnaire of the SHURP study.	N=3471 care workers, in 155 nursing homes.	Switzerland	Secondary data analysis of cross-sectional survey study.		[80]
Age: 31-40 yrs	Emotional exhaustion (mental	p<0.05	multiple regression models	Care worker Personnel	N=3471 care workers, in 155 nursing homes.	Switzerland	Secondary data analysis of cross-		[80]

	health outcomes)			Questionnaire of the SHURP study.			sectional survey study.		
Age: 41-50 yrs	Emotional exhaustion (mental health outcomes)	p<0.05	multiple regression models	Care worker Personnel Questionnaire of the SHURP study.	N=3471 care workers, in 155 nursing homes.	Switzerla nd	Secondary data analysis of cross-sectional survey study.		[80]
Age: >50 yrs	Emotional exhaustion (mental health outcomes)	p<0.05	multiple regression models	Care worker Personnel Questionnaire of the SHURP study.	N=3471 care workers, in 155 nursing homes.	Switzerla nd	Secondary data analysis of cross-sectional survey study.		[80]
Psychosocial work environment : leadership	Emotional exhaustion (mental health outcomes)	p<0.05	multiple regression models	Care worker Personnel Questionnaire of the SHURP study.	N=3471 care workers, in 155 nursing homes.	Switzerla nd	Secondary data analysis of cross-sectional survey study.		[80]
Work stressors: workload	Emotional exhaustion (mental health outcomes)	p<0.05	multiple regression models	Care worker Personnel Questionnaire of the SHURP study.	N=3471 care workers, in 155 nursing homes.	Switzerla nd	Secondary data analysis of cross-sectional survey study.		[80]
Work stressors: lack of job preparation	Emotional exhaustion (mental health outcomes)	p<0.05	multiple regression models	Care worker Personnel Questionnaire of the SHURP study.	N=3471 care workers, in 155 nursing homes.	Switzerla nd	Secondary data analysis of cross-sectional survey study.		[80]
Work stressors: conflict and lack of recognition	Emotional exhaustion (mental health outcomes)	p<0.05	multiple regression models	Care worker Personnel Questionnaire of the SHURP study.	N=3471 care workers, in 155 nursing homes.	Switzerla nd	Secondary data analysis of cross-sectional survey study.		[80]
Background factors (age, gender, working time status, tenure)	psychosomat ic complaints	dR^2: 0.09, p<0.001	hierarchica l moderated regression analysis	Job characteristics (Wall et al. 1996; Prumper et al. 1995). Job strain using Job Demand-Control model.	N=251 workers involved in daily care, nursing homes.	Germany	Cross-sectional questionnaire based study		[81]

				Psychosomatic complaints (von Zerssen 1976). Burnout, the Maslach Burnout Inventory (MBI).					
Background factors (age, gender, working time status, tenure)	turnover intentions	dR^2: 0.11, p<0.001	hierarchical moderated regression analysis	Job characteristics (Wall et al. 1996; Prumper et al. 1995). Job strain using Job Demand-Control model. Psychosomatic complaints (von Zerssen 1976). Burnout, the Maslach Burnout Inventory (MBI).	N=251 workers involved in daily care, nursing homes.	Germany	Cross-sectional questionnaire based study		[81]
Job demands	Emotional exhaustion	Beta: 0.51, p<0.001	hierarchical moderated regression analysis	Job characteristics (Wall et al. 1996; Prumper et al. 1995). Job strain using Job Demand-Control model. Psychosomatic complaints (von Zerssen 1976). Burnout, the Maslach Burnout Inventory (MBI).	N=251 workers involved in daily care, nursing homes.	Germany	Cross-sectional questionnaire based study		[81]
Job demands	psychosomatic complaints	Beta: 0.21, p<0.001	hierarchical moderated regression analysis	Job characteristics (Wall et al. 1996; Prumper et al. 1995). Job strain using Job Demand-Control model. Psychosomatic complaints (von Zerssen 1976).	N=251 workers involved in daily care, nursing homes.	Germany	Cross-sectional questionnaire based study		[81]

				Burnout, the Maslach Burnout Inventory (MBI).					
Job demands	turnover intentions	Beta: 0.24, p<0.001	hierarchical moderated regression analysis	Job characteristics (Wall et al. 1996; Prumper et al. 1995). Job strain using Job Demand-Control model. Psychosomatic complaints (von Zerssen 1976). Burnout, the Maslach Burnout Inventory (MBI).	N=251 workers involved in daily care, nursing homes.	Germany	Cross-sectional questionnaire based study		[81]
Self-efficacy	Emotional exhaustion	Beta: -0.21, p<0.001	hierarchical moderated regression analysis	Job characteristics (Wall et al. 1996; Prumper et al. 1995). Job strain using Job Demand-Control model. Psychosomatic complaints (von Zerssen 1976). Burnout, the Maslach Burnout Inventory (MBI).	N=251 workers involved in daily care, nursing homes.	Germany	Cross-sectional questionnaire based study		[81]
Self-efficacy	psychosomatic complaints	Beta: -0.10, p<0.001	hierarchical moderated regression analysis	Job characteristics (Wall et al. 1996; Prumper et al. 1995). Job strain using Job Demand-Control model. Psychosomatic complaints (von Zerssen 1976).	N=251 workers involved in daily care, nursing homes.	Germany	Cross-sectional questionnaire based study		[81]

				Burnout, the Maslach Burnout Inventory (MBI).					
Self-efficacy	turnover intentions	Beta: -0.22, p<0.001	hierarchical moderated regression analysis	Job characteristics (Wall et al. 1996; Prumper et al. 1995). Job strain using Job Demand-Control model. Psychosomatic complaints (von Zerssen 1976). Burnout, the Maslach Burnout Inventory (MBI).	N=251 workers involved in daily care, nursing homes.	Germany	Cross-sectional questionnaire based study		[81]
rotation schedule	work stress	p<0.05	linear regression	Work stress scale.	N=110 nursing home care attendants	Taiwan	Cross-sectional questionnaire study		[82]
equipment problems	work stress	p<0.05	linear regression	Work stress scale.	N=110 nursing home care attendants	Taiwan	Cross-sectional questionnaire study		[82]
lack of enjoyment of the job	work stress	p<0.05	linear regression	Work stress scale.	N=110 nursing home care attendants	Taiwan	Cross-sectional questionnaire study		[82]
lack of pre-job training	work stress	p<0.05	linear regression	Work stress scale.	N=110 nursing home care attendants	Taiwan	Cross-sectional questionnaire study		[82]
in-job training	work stress	p<0.05	linear regression	Work stress scale.	N=110 nursing home care attendants	Taiwan	Cross-sectional questionnaire study		[82]

night scheduling	work stress	p<0.05	linear regression	Work stress scale.	N=110 nursing home care attendants	Taiwan	Cross-sectional questionnaire study		[82]
younger age	work stress	p<0.05	linear regression	Work stress scale.	N=110 nursing home care attendants	Taiwan	Cross-sectional questionnaire study		[82]
Patient/client disease severity	workload	IRR: 1.65, 95% CI: 1.31-2.07	multivariate analysis.	Comorbidity, Charlson Index. Functional status, Barthel index. Cognitive status, Pfeiffer scale. Prescence and risk of ulcers, Braden Scale. Social risk, Gijon social scale.	N=1068 home care patients	Spain	Longitudinal study		[83]
Major cognitive impairment	workload	IRR: 0.78, 95% CI: 0.63-0.98	multivariate analysis.	Comorbidity, Charlson Index. Functional status, Barthel index. Cognitive status, Pfeiffer scale. Prescence and risk of ulcers, Braden Scale. Social risk, Gijon social scale.	N=1068 home care patients	Spain	Longitudinal study		[83]
Feeling responsible	workload	n/a	qualitative content analysis	multi-dimensional dementia assessment scale and interviews.	N=11 nurses and 11 carers, nursing homes.	Norway	Desriptive, comparative cross-sectional study		[84]
feeling burdened	workload	n/a	qualitative content analysis	multi-dimensional dementia assessment scale and interviews.	N=11 nurses and 11 carers, nursing homes.	Norway	Desriptive, comparative cross-sectional study		[84]

feeling ambivalent	workload	n/a	qualitative content analysis	multi-dimensional dementia assessment scale and interviews.	N=11 nurses and 11 carers, nursing homes.	Norway	Desriptive, comparative cross-sectional study		[84]
Job satisfaction: overall	intention to leave	p<0.05	t-test	Multi-item questionnaires on work satisfaction, patient care and staff health.	N=299 health and care staff in elderly care	Sweden	Cross-sectional survey based study		[85]
Job satisfaction: personal development	intention to leave	p<0.05	t-test	Multi-item questionnaires on work satisfaction, patient care and staff health.	N=299 health and care staff in elderly care	Sweden	Cross-sectional survey based study		[85]
Job satisfaction: workload	intention to leave	p<0.05	t-test	Multi-item questionnaires on work satisfaction, patient care and staff health.	N=299 health and care staff in elderly care	Sweden	Cross-sectional survey based study		[85]
Job satisfaction: criticism	intention to leave	p<0.05	t-test	Multi-item questionnaires on work satisfaction, patient care and staff health.	N=299 health and care staff in elderly care	Sweden	Cross-sectional survey based study		[85]
Job satisfaction: expectations and demands	intention to leave	p<0.05	t-test	Multi-item questionnaires on work satisfaction, patient care and staff health.	N=299 health and care staff in elderly care	Sweden	Cross-sectional survey based study		[85]
Job satisfaction: cooperation	intention to leave	p<0.05	t-test	Multi-item questionnaires on work satisfaction, patient care and staff health.	N=299 health and care staff in elderly care	Sweden	Cross-sectional survey based study		[85]
Job satisfaction: Internal motivation	intention to leave	p<0.05	t-test	Multi-item questionnaires on work satisfaction,	N=299 health and care staff in elderly care	Sweden	Cross-sectional survey based study		[85]

				patient care and staff health.					
Job satisfaction: external motivation	intention to leave	p<0.05	t-test	Multi-item questionnaires on work satisfaction, patient care and staff health.	N=299 health and care staff in elderly care	Sweden	Cross-sectional survey based study		[85]
Job satisfaction: position in the group	intention to leave	p<0.05	t-test	Multi-item questionnaires on work satisfaction, patient care and staff health.	N=299 health and care staff in elderly care	Sweden	Cross-sectional survey based study		[85]
Workload	Stress symptoms	Beta: 0.415, p<0.001	multiple regression analysis	Multi-item questionnaires on work satisfaction, patient care and staff health.	N=299 health and care staff in elderly care	Sweden	Cross-sectional survey based study		[85]
Cooperation	Stress symptoms	Beta: 0.12, p<0.05	multiple regression analysis	Multi-item questionnaires on work satisfaction, patient care and staff health.	N=299 health and care staff in elderly care	Sweden	Cross-sectional survey based study		[85]
Age	Stress symptoms	Beta: 0.151, p=0.001	multiple regression analysis	Multi-item questionnaires on work satisfaction, patient care and staff health.	N=299 health and care staff in elderly care	Sweden	Cross-sectional survey based study		[85]
Expectations and demands	Stress symptoms	Beta: 0.148, p=0.007	multiple regression analysis	Multi-item questionnaires on work satisfaction, patient care and staff health.	N=299 health and care staff in elderly care	Sweden	Cross-sectional survey based study		[85]
Personal development	Stress symptoms	Beta: 0.189, p=0.006	multiple regression analysis	Multi-item questionnaires on work satisfaction, patient care and staff health.	N=299 health and care staff in elderly care	Sweden	Cross-sectional survey based study		[85]

Internal motivation	Stress symptoms	Beta: -0.133, p=0.025	multiple regression analysis	Multi-item questionnaires on work satisfaction, patient care and staff health.	N=299 health and care staff in elderly care	Sweden	Cross-sectional survey based study		[85]
Organisation al boundaries	Reduce significance of nurse patient relationship	n/a	qualitative analysis	semi-structured interview	N=10 nurses, 8 patients, homecare	Norway	Semi-structured interviews		[61]
time constraint	Reduce significance of nurse patient relationship	n/a	qualitative analysis	semi-structured interview	N=10 nurses, 8 patients, homecare	Norway	Semi-structured interviews		[61]
High workload	Reduce significance of nurse patient relationship	n/a	qualitative analysis	semi-structured interview	N=10 nurses, 8 patients, homecare	Norway	Semi-structured interviews		[61]
disharmony between nurse "competence and patients" complex disease	Reduce significance of nurse patient relationship	n/a	qualitative analysis	semi-structured interview	N=10 nurses, 8 patients, homecare	Norway	Semi-structured interviews		[61]
Usociable hours	Consider leaving	n/a	qualitative analysis	Based on recruitment and retention of healthcare works survey (Sinclair et al. 2000)	N=45 home care workers	United Kingdom	Cross-sectional questionnaire based study		[86]
Lack of management support	Consider leaving	n/a	qualitative analysis	Based on recruitment and retention of healthcare works survey (Sinclair et al. 2000)	N=45 home care workers	United Kingdom	Cross-sectional questionnaire based study		[86]

Workload	Consider leaving	n/a	qualitative analysis	Based on recruitment and retention of healthcare works survey (Sinclair et al. 2000)	N=45 home care workers	United Kingdom	Cross-sectional questionnaire based study		[86]
Lack of client support	Consider leaving	n/a	qualitative analysis	Based on recruitment and retention of healthcare works survey (Sinclair et al. 2000)	N=45 home care workers	United Kingdom	Cross-sectional questionnaire based study		[86]
Pay and remuneration	Consider leaving	n/a	qualitative analysis	Based on recruitment and retention of healthcare works survey (Sinclair et al. 2000)	N=45 home care workers	United Kingdom	Cross-sectional questionnaire based study		[86]
Paperwork	Consider leaving	n/a	qualitative analysis	Based on recruitment and retention of healthcare works survey (Sinclair et al. 2000)	N=45 home care workers	United Kingdom	Cross-sectional questionnaire based study		[86]
Other reasons (e.g., temporary employment)	Consider leaving	n/a	qualitative analysis	Based on recruitment and retention of healthcare works survey (Sinclair et al. 2000)	N=45 home care workers	United Kingdom	Cross-sectional questionnaire based study		[86]
Usociable and unpredictable working pattern	Conflict with family, social life	n/a	qualitative analysis	Based on recruitment and retention of healthcare works survey (Sinclair et al. 2000)	N=45 home care workers	United Kingdom	Cross-sectional questionnaire based study		[86]
Task-oriented leadership style	job satisfaction	n/a; positive relationship	Multilevel analysis	Job satisfaction (Brayfield and Rothe 1951). Leadership style (Yukl 2010; Northouse 2009; Bass and Bass 2008).	N=444 registered nurses, nursing homes	Norway	Cross-sectional survey based study		[87]

Relationship -oriented leadership style	job satisfaction	n/a; positive relationship	Multilevel analysis	Job satisfaction (Brayfield and Rothe 1951). Leadership style (Yukl 2010; Northouse 2009; Bass and Bass 2008).	N=444 registered nurses, nursing homes	Norway	Cross-sectional survey based study		[87]
Working time	Emotional exhaustion	Beta: 0.16, p<0.05	hierarchical moderated regression analysis	Goal incongruence (Stengel 1990). Self-control demands (Neubach and Schmidt 2006). Job strain, Maslach Burnout Inventory and von Zerssen 1976.	N=242 nursing staff, elderly care	Germany	Cross-sectional questionnaire based study		[88]
Goal incongruence (between personal and organisational goals)	Emotional exhaustion	Beta: 0.45, p<0.01	hierarchical moderated regression analysis	Goal incongruence (Stengel 1990). Self-control demands (Neubach and Schmidt 2006). Job strain, Maslach Burnout Inventory and von Zerssen 1976.	N=242 nursing staff, elderly care	Germany	Cross-sectional questionnaire based study		[88]
Self-control demands	Emotional exhaustion	Beta: 0.28, p<0.01	hierarchical moderated regression analysis	Goal incongruence (Stengel 1990). Self-control demands (Neubach and Schmidt 2006). Job strain, Maslach Burnout Inventory and von Zerssen 1976.	N=242 nursing staff, elderly care	Germany	Cross-sectional questionnaire based study		[88]
Goal incongruence (between personal and	Depersonalisation	Beta: 0.19, p<0.01	hierarchical moderated regression analysis	Goal incongruence (Stengel 1990). Self-control demands (Neubach and Schmidt 2006). Job	N=242 nursing staff, elderly care	Germany	Cross-sectional questionnaire based study		[88]

organisational goals)				strain, Maslach Burnout Inventory and von Zerssen 1976.					
Self-control demands	Depersonalisation	Beta: 0.17, p<0.01	hierarchical moderated regression analysis	Goal incongruence (Stengel 1990). Self-control demands (Neubach and Schmidt 2006). Job strain, Maslach Burnout Inventory and von Zerssen 1976.	N=242 nursing staff, elderly care	Germany	Cross-sectional questionnaire based study		[88]
Age	psychosomatic complaints	Beta: 0.13, p<0.01	hierarchical moderated regression analysis	Goal incongruence (Stengel 1990). Self-control demands (Neubach and Schmidt 2006). Job strain, Maslach Burnout Inventory and von Zerssen 1976.	N=242 nursing staff, elderly care	Germany	Cross-sectional questionnaire based study		[88]
Goal incongruence (between personal and organisational goals)	psychosomatic complaints	Beta: 0.29, p<0.01	hierarchical moderated regression analysis	Goal incongruence (Stengel 1990). Self-control demands (Neubach and Schmidt 2006). Job strain, Maslach Burnout Inventory and von Zerssen 1976.	N=242 nursing staff, elderly care	Germany	Cross-sectional questionnaire based study		[88]
Self-control demands	psychosomatic complaints	Beta: 0.09, p<0.05	hierarchical moderated regression analysis	Goal incongruence (Stengel 1990). Self-control demands (Neubach and Schmidt 2006). Job strain, Maslach Burnout Inventory	N=242 nursing staff, elderly care	Germany	Cross-sectional questionnaire based study		[88]

				and von Zerssen 1976.					
Gender	time lost (absenteeism)	Beta: -0.19, p<0.01	hierarchical moderated regression analysis	Goal incongruence (Stengel 1990). Self-control demands (Neubach and Schmidt 2006). Job strain, Maslach Burnout Inventory and von Zerssen 1976.	N=242 nursing staff, elderly care	Germany	Cross-sectional questionnaire based study		[88]
Goal incongruence (between personal and organisational goals)	time lost (absenteeism)	Beta: 0.19, p<0.01	hierarchical moderated regression analysis	Goal incongruence (Stengel 1990). Self-control demands (Neubach and Schmidt 2006). Job strain, Maslach Burnout Inventory and von Zerssen 1976.	N=242 nursing staff, elderly care	Germany	Cross-sectional questionnaire based study		[88]
Self-control demands	time lost (absenteeism)	Beta: 0.14, p<0.05	hierarchical moderated regression analysis	Goal incongruence (Stengel 1990). Self-control demands (Neubach and Schmidt 2006). Job strain, Maslach Burnout Inventory and von Zerssen 1976.	N=242 nursing staff, elderly care	Germany	Cross-sectional questionnaire based study		[88]
Goal incongruence (between personal and organisational goals)	Absence frequency	Beta: 0.28, p<0.01	hierarchical moderated regression analysis	Goal incongruence (Stengel 1990). Self-control demands (Neubach and Schmidt 2006). Job strain, Maslach Burnout Inventory	N=242 nursing staff, elderly care	Germany	Cross-sectional questionnaire based study		[88]

				and von Zerssen 1976.					
Meaning	job satisfaction	Beta: 0.252, p=0.005	multiple regression analysis	Satisfaction with Work Questionnaire (SWQ), Conditions of Work Effectiveness Questionnaire II (CWEQ II).	N=125 caregivers with no formal competence	Sweden	Comparatie and correlative study design.		[119]
Self-determinatio n	job satisfaction	Beta: 0.19, p=0.045	multiple regression analysis	Satisfaction with Work Questionnaire (SWQ), Conditions of Work Effectiveness Questionnaire II (CWEQ II).	N=125 caregivers with no formal competence	Sweden	Comparatie and correlative study design.		[119]
Impact	job satisfaction	Beta: 0.301, p=0.002	multiple regression analysis	Satisfaction with Work Questionnaire (SWQ), Conditions of Work Effectiveness Questionnaire II (CWEQ II).	N=125 caregivers with no formal competence	Sweden	Comparatie and correlative study design.		[119]
Opportunity	job satisfaction	Beta: 0.254, p=0.011	multiple regression analysis	Satisfaction with Work Questionnaire (SWQ), Conditions of Work Effectiveness Questionnaire II (CWEQ II).	N=125 caregivers with no formal competence	Sweden	Comparatie and correlative study design.		[119]
Self-determinatio n	Stress symptoms	Beta: 0.283, p=0.01	multiple regression analysis	Satisfaction with Work Questionnaire (SWQ), Conditions of Work Effectiveness Questionnaire II (CWEQ II).	N=125 caregivers with no formal competence	Sweden	Comparatie and correlative study design.		[119]
Competence	quality of care	Beta: 0.317, p=0.003	multiple regression analysis	Satisfaction with Work Questionnaire (SWQ), Conditions of Work Effectiveness	N=125 caregivers with no formal competence	Sweden	Comparatie and correlative study design.		[119]

				Questionnaire II (CWEQ II).					
Opportunity	quality of care	Beta: 0.309, p=0.002	multiple regression analysis	Satisfaction with Work Questionnaire (SWQ), Conditions of Work Effectiveness Questionnaire II (CWEQ II).	N=125 caregivers with no formal competence	Sweden	Comparatie and correlative study design.		[119]
Resources	quality of care	Beta: 0.327, p=0.003	multiple regression analysis	Satisfaction with Work Questionnaire (SWQ), Conditions of Work Effectiveness Questionnaire II (CWEQ II).	N=125 caregivers with no formal competence	Sweden	Comparatie and correlative study design.		[119]
job activities scale	quality of care	Beta: -0.371, p<0.001	multiple regression analysis	Satisfaction with Work Questionnaire (SWQ), Conditions of Work Effectiveness Questionnaire II (CWEQ II).	N=125 caregivers with no formal competence	Sweden	Comparatie and correlative study design.		[119]
Meaning	job satisfaction	Beta: 0.339, p<0.001	multiple regression analysis	Satisfaction with Work Questionnaire (SWQ), Conditions of Work Effectiveness Questionnaire II (CWEQ II).	N=445 caregivers with formal competence	Sweden	Comparatie and correlative study design.		[119]
Self-determination	job satisfaction	Beta: 0211, p<0.001	multiple regression analysis	Satisfaction with Work Questionnaire (SWQ), Conditions of Work Effectiveness Questionnaire II (CWEQ II).	N=445 caregivers with formal competence	Sweden	Comparatie and correlative study design.		[119]
Impact	job satisfaction	Beta: 0.276, p<0.001	multiple regression analysis	Satisfaction with Work Questionnaire (SWQ), Conditions of Work Effectiveness Questionnaire II (CWEQ II).	N=445 caregivers with formal competence	Sweden	Comparatie and correlative study design.		[119]

Opportunity	job satisfaction	Beta: 0.281, p<0.001	multiple regression analysis	Satisfaction with Work Questionnaire (SWQ), Conditions of Work Effectiveness Questionnaire II (CWEQ II).	N=445 caregivers with formal competence	Sweden	Comparatie and correlative study design.		[119]
Support	job satisfaction	Beta: 0.123, p=0.008	multiple regression analysis	Satisfaction with Work Questionnaire (SWQ), Conditions of Work Effectiveness Questionnaire II (CWEQ II).	N=445 caregivers with formal competence	Sweden	Comparatie and correlative study design.		[119]
Resources	job satisfaction	Beta: 0.218,p<0.001	multiple regression analysis	Satisfaction with Work Questionnaire (SWQ), Conditions of Work Effectiveness Questionnaire II (CWEQ II).	N=445 caregivers with formal competence	Sweden	Comparatie and correlative study design.		[119]
job activities scale	job satisfaction	Beta: 0.185, p<0.001	multiple regression analysis	Satisfaction with Work Questionnaire (SWQ), Conditions of Work Effectiveness Questionnaire II (CWEQ II).	N=445 caregivers with formal competence	Sweden	Comparatie and correlative study design.		[119]
organisational relationship scale	job satisfaction	Beta: 0.103, p=0.02	multiple regression analysis	Satisfaction with Work Questionnaire (SWQ), Conditions of Work Effectiveness Questionnaire II (CWEQ II).	N=445 caregivers with formal competence	Sweden	Comparatie and correlative study design.		[119]
Self-determination	Stress symptoms	Beta: 0.201, p<0.001	multiple regression analysis	Satisfaction with Work Questionnaire (SWQ), Conditions of Work Effectiveness Questionnaire II (CWEQ II).	N=445 caregivers with formal competence	Sweden	Comparatie and correlative study design.		[119]

Impact	Stress symptoms	Beta: 0.198, p=0.001	multiple regression analysis	Satisfaction with Work Questionnaire (SWQ), Conditions of Work Effectiveness Questionnaire II (CWEQ II).	N=445 caregivers with formal competence	Sweden	Comparatie and correlative study design.		[119]
Support	Stress symptoms	Beta: 0.160, p=0.003	multiple regression analysis	Satisfaction with Work Questionnaire (SWQ), Conditions of Work Effectiveness Questionnaire II (CWEQ II).	N=445 caregivers with formal competence	Sweden	Comparatie and correlative study design.		[119]
Resources	Stress symptoms	Beta: 0.379, p<0.001	multiple regression analysis	Satisfaction with Work Questionnaire (SWQ), Conditions of Work Effectiveness Questionnaire II (CWEQ II).	N=445 caregivers with formal competence	Sweden	Comparatie and correlative study design.		[119]
job activities scale	Stress symptoms	Beta: 0.105, p=0.046	multiple regression analysis	Satisfaction with Work Questionnaire (SWQ), Conditions of Work Effectiveness Questionnaire II (CWEQ II).	N=445 caregivers with formal competence	Sweden	Comparatie and correlative study design.		[119]
organisation al relationship scale	Stress symptoms	Beta: -0.153, p=0.003	multiple regression analysis	Satisfaction with Work Questionnaire (SWQ), Conditions of Work Effectiveness Questionnaire II (CWEQ II).	N=445 caregivers with formal competence	Sweden	Comparatie and correlative study design.		[119]
Meaning	quality of care	Beta: 0.216, p<0.001	multiple regression analysis	Satisfaction with Work Questionnaire (SWQ), Conditions of Work Effectiveness Questionnaire II (CWEQ II).	N=445 caregivers with formal competence	Sweden	Comparatie and correlative study design.		[119]

Competence	quality of care	Beta: 0.129, p=0.006	multiple regression analysis	Satisfaction with Work Questionnaire (SWQ), Conditions of Work Effectiveness Questionnaire II (CWEQ II).	N=445 caregivers with formal competence	Sweden	Comparatie and correlative study design.		[119]
Impact	quality of care	Beta: 0.241, p<0.001	multiple regression analysis	Satisfaction with Work Questionnaire (SWQ), Conditions of Work Effectiveness Questionnaire II (CWEQ II).	N=445 caregivers with formal competence	Sweden	Comparatie and correlative study design.		[119]
Opportunity	quality of care	Beta: 0.117, p=0.016	multiple regression analysis	Satisfaction with Work Questionnaire (SWQ), Conditions of Work Effectiveness Questionnaire II (CWEQ II).	N=445 caregivers with formal competence	Sweden	Comparatie and correlative study design.		[119]
Support	quality of care	Beta: 0.140, p=0.011	multiple regression analysis	Satisfaction with Work Questionnaire (SWQ), Conditions of Work Effectiveness Questionnaire II (CWEQ II).	N=445 caregivers with formal competence	Sweden	Comparatie and correlative study design.		[119]
Resources	quality of care	Beta: 0.219, p<0.001	multiple regression analysis	Satisfaction with Work Questionnaire (SWQ), Conditions of Work Effectiveness Questionnaire II (CWEQ II).	N=445 caregivers with formal competence	Sweden	Comparatie and correlative study design.		[119]
organisational relationship scale	quality of care	Beta: 0.143, p=0.006	multiple regression analysis	Satisfaction with Work Questionnaire (SWQ), Conditions of Work Effectiveness Questionnaire II (CWEQ II).	N=445 caregivers with formal competence	Sweden	Comparatie and correlative study design.		[119]

Involvement in interdisciplinary care plan meetings	staff turnover	Beta: -0.365, $p<0.05$	regression analysis	Resident Assessment Instrument (RAI)	N=254 nursing aides, nursing homes	USA	Survey based study		[72]
Facility characterstics: proprietary status (profit vs. non-profit)	staff turnover	Beta: 0.542, $p<0.05$	regression analysis	Resident Assessment Instrument (RAI)	N=254 nursing aides, nursing homes	USA	Survey based study		[72]
County level characteristics: Unemployment rate	staff turnover	Beta: -0.022, $p<0.05$	regression analysis	Resident Assessment Instrument (RAI)	N=254 nursing aides, nursing homes	USA	Survey based study		[72]
County level characteristics: total number of beds in county	staff turnover	Beta: 0.804, $p<0.05$	regression analysis	Resident Assessment Instrument (RAI)	N=254 nursing aides, nursing homes	USA	Survey based study		[72]
County level characteristics: per-capita income	staff turnover	Beta: -0.215, $p<0.001$	regression analysis	Resident Assessment Instrument (RAI)	N=254 nursing aides, nursing homes	USA	Survey based study		[72]
Work environment: quality of care - high	conflict and lack of recognition	Beta: -0.287, $p<0.001$	multivariate linear GEE models	SHURP study, Health profession stress inventory (HPSI), Practice environment scale-nursing work index (PES-NWI), Safety attitudes questionnaire (SAQ).	N=3922 care workers, in 156 nursing homes.	Switzerland	Cross-sectional questionnaire based study, SHURP		[89]

Work environment : quality of care - high	workload	Beta -0.326, p<0.001	multivariate linear GEE models	SHURP study, Health profession stress inventory (HPSI), Practice environment scale-nursing work index (PES-NWI), Safety attitudes questionnaire (SAQ).	N=3922 care workers, in 156 nursing homes.	Switzerland	Cross-sectional questionnaire based study, SHURP		[89]
Work environment : collaboration with team colleagues - High	Conflict and lack of recognition	Beta: -0.164, p=0.006	multivariate linear GEE models	SHURP study, Health profession stress inventory (HPSI), Practice environment scale-nursing work index (PES-NWI), Safety attitudes questionnaire (SAQ).	N=3922 care workers, in 156 nursing homes.	Switzerland	Cross-sectional questionnaire based study, SHURP		[89]
Work environment : PES-NWI (practice environment scale-nursing work index) leadership	conflict and lack of recognition	Beta: -0.383, p<0.001	multivariate linear GEE models	SHURP study, Health profession stress inventory (HPSI), Practice environment scale-nursing work index (PES-NWI), Safety attitudes questionnaire (SAQ).	N=3922 care workers, in 156 nursing homes.	Switzerland	Cross-sectional questionnaire based study, SHURP		[89]
Work environment : PES-NWI (practice environment scale-nursing work index) leadership	lack of preparation	Beta: -0.045, p=0.05	multivariate linear GEE models	SHURP study, Health profession stress inventory (HPSI), Practice environment scale-nursing work index (PES-NWI), Safety attitudes questionnaire (SAQ).	N=3922 care workers, in 156 nursing homes.	Switzerland	Cross-sectional questionnaire based study, SHURP		[89]
Work environment : PES-NWI (practice	conflict and lack of recognition	Beta: -0.174, p<0.001	multivariate linear GEE models	SHURP study, Health profession stress inventory (HPSI), Practice environment	N=3922 care workers, in 156 nursing homes.	Switzerland	Cross-sectional questionnaire		[89]

environment scale-nursing work index) staffing and resources adequacy				scale-nursing work index (PES-NWI), Safety attitudes questionnaire (SAQ).			based study, SHURP		
Work environment: PES-NWI (practice environment scale-nursing work index) staffing and resources adequacy	workload	Beta: -0.555, p<0.001	multivariate linear GEE models	SHURP study, Health profession stress inventory (HPSI), Practice environment scale-nursing work index (PES-NWI), Safety attitudes questionnaire (SAQ).	N=3922 care workers, in 156 nursing homes.	Switzerland	Cross-sectional questionnaire based study, SHURP		[89]
Work environment: PES-NWI (practice environment scale-nursing work index) staffing and resources adequacy	lack of preparation	Beta: -0.126, p<0.001	multivariate linear GEE models	SHURP study, Health profession stress inventory (HPSI), Practice environment scale-nursing work index (PES-NWI), Safety attitudes questionnaire (SAQ).	N=3922 care workers, in 156 nursing homes.	Switzerland	Cross-sectional questionnaire based study, SHURP		[89]
Resident characteristics: Verbal agression against care worker - once per week or less	conflict and lack of recognition	Beta: 0.05, p=0.017	multivariate linear GEE models	SHURP study, Health profession stress inventory (HPSI), Practice environment scale-nursing work index (PES-NWI), Safety attitudes questionnaire (SAQ).	N=3922 care workers, in 156 nursing homes.	Switzerland	Cross-sectional questionnaire based study, SHURP		[89]

Resident characteristics: Verbal agression against care worker - once per week or less	workload	Beta: 0.188, p<0.001	multivariate linear GEE models	SHURP study, Health profession stress inventory (HPSI), Practice environment scale-nursing work index (PES-NWI), Safety attitudes questionnaire (SAQ).	N=3922 care workers, in 156 nursing homes.	Switzerland	Cross-sectional questionnaire based study, SHURP		[89]
Resident characteristics: Verbal agression against care worker - once per week or less	lack of preparation	Beta: 0.099, p<0.001	multivariate linear GEE models	SHURP study, Health profession stress inventory (HPSI), Practice environment scale-nursing work index (PES-NWI), Safety attitudes questionnaire (SAQ).	N=3922 care workers, in 156 nursing homes.	Switzerland	Cross-sectional questionnaire based study, SHURP		[89]
Resident characteristics: Verbal agression against care worker - several times/week to several times/day	conflict and lack of recognition	Beta: 0.097, p=0.002	multivariate linear GEE models	SHURP study, Health profession stress inventory (HPSI), Practice environment scale-nursing work index (PES-NWI), Safety attitudes questionnaire (SAQ).	N=3922 care workers, in 156 nursing homes.	Switzerland	Cross-sectional questionnaire based study, SHURP		[89]
Resident characteristics: Verbal agression against care worker - several times/week to several times/day	workload	Beta: 0.297, p<0.001	multivariate linear GEE models	SHURP study, Health profession stress inventory (HPSI), Practice environment scale-nursing work index (PES-NWI), Safety attitudes questionnaire (SAQ).	N=3922 care workers, in 156 nursing homes.	Switzerland	Cross-sectional questionnaire based study, SHURP		[89]

Resident characteristics: Verbal agression against care worker - several times/week to several times/day	lack of preparation	Beta: 0.09, p=0.008	multivariate linear GEE models	SHURP study, Health profession stress inventory (HPSI), Practice environment scale-nursing work index (PES-NWI), Safety attitudes questionnaire (SAQ).	N=3922 care workers, in 156 nursing homes.	Switzerland	Cross-sectional questionnaire based study, SHURP		[89]
Patient function	workload	n/a	n/a	Resident Assessment Instrument Healthcare version 2.0 (RAI-HC v. 2).	N=4010 assessments, care givers in home care.	11 European countries	cross-sectional retrospective study		[90]
role conflict	burnout	0.32 (p<0.001)	Beta - causal model	Role conflict tool, role-ambiguity tool, Organization Commitment Questionnaire, Maslach burnout tool, turnover-intention tool.	N=500 nurses, general hospital	South Korea	survey-based causal model		[91]
role ambiguity	burnout	0.34 (p<0.001)	Beta - causal model	Role conflict tool, role-ambiguity tool, Organization Commitment Questionnaire, Maslach burnout tool, turnover-intention tool.	N=500 nurses, general hospital	South Korea	survey-based causal model		[91]
role conflict	stress of role ambiguity	0.22 (p<0.001)	Beta - causal model	Role conflict tool, role-ambiguity tool, Organization Commitment Questionnaire, Maslach burnout tool, turnover-intention tool.	N=500 nurses, general hospital	South Korea	survey-based causal model		[91]

role ambiguity	stress of role conflict	0.22 (p<0.001)	Beta - causal model	Role conflict tool, role-ambiguity tool, Organization Commitment Questionnaire, Maslach burnout tool, turnover-intention tool.	N=500 nurses, general hospital	South Korea	survey-based causal model		[91]
ethnicity - Hispanic	Very satisfied with home care job	0.338 (95% CI: 0.211-0.542)	Odds ratio - multivariate logistic regression	Job demand-control/support framework.	N=1614 home care workers, 71% care for relatives(!)	USA	telephone interview		[92]
Age	Very satisfied with home care job	1.019 (95% CI: 1.009-1.029)	Odds ratio - multivariate logistic regression	Job demand-control/support framework.	N=1614 home care workers, 71% care for relatives(!)	USA	telephone interview		[92]
Education - less than high school	Very satisfied with home care job	1.414 (95% CI: 1.021-1.956)	Odds ratio - multivariate logistic regression	Job demand-control/support framework.	N=1614 home care workers, 71% care for relatives(!)	USA	telephone interview		[92]
consumer relative/coresidence - not related, live in same home	Very satisfied with home care job	0.448 (95% CI: 0.222-0.905)	Odds ratio - multivariate logistic regression	Job demand-control/support framework.	N=1614 home care workers, 71% care for relatives(!)	USA	telephone interview		[92]
subjective physical demands	Very satisfied with home care job	1.246 (95% CI: 1.089-1.426)	Odds ratio - multivariate logistic regression	Job demand-control/support framework.	N=1614 home care workers, 71% care for relatives(!)	USA	telephone interview		[92]
physical task frequency	Very satisfied with home care job	1.747 (95% CI: 1.334-2.289)	Odds ratio - multivariat	Job demand-control/support framework.	N=1614 home care workers, 71% care for relatives(!)	USA	telephone interview		[92]

			e logistic regression						
emotional suppression	Very satisfied with home care job	1.235 (95% CI: 1.097-1.390)	Odds ratio - multivariate logistic regression	Job demand-control/support framework.	N=1614 home care workers, 71% care for relatives(!)	USA	telephone interview		[92]
abuse	Very satisfied with home care job	0.634 (95% CI: 0.499-0.805)	Odds ratio - multivariate logistic regression	Job demand-control/support framework.	N=1614 home care workers, 71% care for relatives(!)	USA	telephone interview		[92]
overtime hours	Very satisfied with home care job	0.989 (95% CI: 0.979-0.999)	Odds ratio - multivariate logistic regression	Job demand-control/support framework.	N=1614 home care workers, 71% care for relatives(!)	USA	telephone interview		[92]
more than one consumer	Very satisfied with home care job	0.595 (95% CI: 0.433-0.818)	Odds ratio - multivariate logistic regression	Job demand-control/support framework.	N=1614 home care workers, 71% care for relatives(!)	USA	telephone interview		[92]
unable to see doctor due to cost	Very satisfied with home care job	0.668 (95% CI: 0.504-0.886)	Odds ratio - multivariate logistic regression	Job demand-control/support framework.	N=1614 home care workers, 71% care for relatives(!)	USA	telephone interview		[92]
not worried about becoming unemployed	Very satisfied with home care job	1.49 (95% CI: 1.081-2.054)	Odds ratio - multivariate logistic regression	Job demand-control/support framework.	N=1614 home care workers, 71% care for relatives(!)	USA	telephone interview		[92]
importance of belonging to union - very important	Very satisfied with home care job	2.632 (95% CI: 1.782-3.888)	Odds ratio - multivariate logistic regression	Job demand-control/support framework.	N=1614 home care workers, 71% care for relatives(!)	USA	telephone interview		[92]

social support	Very satisfied with home care job	1.412 (95% CI: 1.195-1669)	Odds ratio - multivariate logistic regression	Job demand-control/support framework.	N=1614 home care workers, 71% care for relatives(!)	USA	telephone interview		[92]
dementia care specialization: specialists	total strain	1.744 (p=0.004)	Beta - correlation	Strain in Dementia Care Scale (SDCS), Creative Climate Questionnaire (CCQ)	N=69 Dementia care specialists and other staff in Swedish home care services	Sweden	Cross-sectional survey study		[53]
dementia care specialization: specialists	frustrated empathy	2.353 (p=0.001)	Beta - correlation	Strain in Dementia Care Scale (SDCS), Creative Climate Questionnaire (CCQ)	N=69 Dementia care specialists and other staff in Swedish home care services	Sweden	Cross-sectional survey study		[53]
dementia care specialization: specialists	difficulties understanding and interpreting	1.229 (p=0.047)	Beta - correlation	Strain in Dementia Care Scale (SDCS), Creative Climate Questionnaire (CCQ)	N=69 Dementia care specialists and other staff in Swedish home care services	Sweden	Cross-sectional survey study		[53]
First language: not Swedish	difficulties understanding and interpreting	1.981 (p=0.001)	Beta - correlation	Strain in Dementia Care Scale (SDCS), Creative Climate Questionnaire (CCQ)	N=69 Dementia care specialists and other staff in Swedish home care services	Sweden	Cross-sectional survey study		[53]
dementia care specialization: specialists	balancing competing needs	2.065 (p=0.027)	Beta - correlation	Strain in Dementia Care Scale (SDCS), Creative Climate Questionnaire (CCQ)	N=69 Dementia care specialists and other staff in Swedish home care services	Sweden	Cross-sectional survey study		[53]
dementia care specialization: specialists	balancing emotional involvement	1.768 (p=0.023)	Beta - correlation	Strain in Dementia Care Scale (SDCS), Creative Climate Questionnaire (CCQ)	N=69 Dementia care specialists and other staff in Swedish home care services	Sweden	Cross-sectional survey study		[53]
dementia care	lack of recognition	1.921 (p=0.009)	Beta - correlation	Strain in Dementia Care Scale (SDCS),	N=69 Dementia care specialists and other staff in	Sweden	Cross-sectional survey study		[53]

specialization: specialists				Creative Climate Questionnaire (CCQ)	Swedish home care services				
organisational climate - stagnated	lack of recognition	1.921 (p=0.002)	Beta - correlation	Strain in Dementia Care Scale (SDCS), Creative Climate Questionnaire (CCQ)	N=69 Dementia care specialists and other staff in Swedish home care services	Sweden	Cross-sectional survey study		[53]
internal medicine wards	Stress of conscience	0.098 (p<=0.05)	Beta - multiple regression analysis	Stress of conscience questionnaire (SCQ), PCQ, General Nordic Questionnaire for Psychological and Social Factors at Work (QPS Nordic).	N=469 Healthcare personell with patient contact	Sweden	Cross-sectional survey		[93]
PCQ/conscience warns us against hurting others	Stress of conscience	0.164 (p<=0.001)	Beta - multiple regression analysis	Stress of conscience questionnaire (SCQ), PCQ, General Nordic Questionnaire for Psychological and Social Factors at Work (QPS Nordic).	N=469 Healthcare personell with patient contact	Sweden	Cross-sectional survey		[93]
PCQ/I follow my conscience in my work	Stress of conscience	-0.144 (p<=0.01)	Beta - multiple regression analysis	Stress of conscience questionnaire (SCQ), PCQ, General Nordic Questionnaire for Psychological and Social Factors at Work (QPS Nordic).	N=469 Healthcare personell with patient contact	Sweden	Cross-sectional survey		[93]
PCQ/I have to deaden my conscience to keep working in healthcare	Stress of conscience	0.158 (p<=0.001)	Beta - multiple regression analysis	Stress of conscience questionnaire (SCQ), PCQ, General Nordic Questionnaire for Psychological and Social Factors at Work (QPS Nordic).	N=469 Healthcare personell with patient contact	Sweden	Cross-sectional survey		[93]

Sense of moral burden - ability to sense patient's needs means doing more than one has the strength for	Stress of conscience	0.264 (p<=0.001)	Beta - multiple regression analysis	Stress of conscience questionnaire (SCQ), PCQ, General Nordic Questionnaire for Psychological and Social Factors at Work (QPS Nordic).	N=469 Healthcare personell with patient contact	Sweden	Cross-sectional survey		[93]
Sense of moral burden - difficult to deal with feelings aroused when a patient is suffering	Stress of conscience	0.110 (p<=0.05)	Beta - multiple regression analysis	Stress of conscience questionnaire (SCQ), PCQ, General Nordic Questionnaire for Psychological and Social Factors at Work (QPS Nordic).	N=469 Healthcare personell with patient contact	Sweden	Cross-sectional survey		[93]
Sense of moral burden - ability to sense patient's needs means feeling inadequate	Stress of conscience	0.147 (p<=0.01)	Beta - multiple regression analysis	Stress of conscience questionnaire (SCQ), PCQ, General Nordic Questionnaire for Psychological and Social Factors at Work (QPS Nordic).	N=469 Healthcare personell with patient contact	Sweden	Cross-sectional survey		[93]
Support from superiors	Stress of conscience	-0.13 (p<=0.001)	Beta - multiple regression analysis	Stress of conscience questionnaire (SCQ), PCQ, General Nordic Questionnaire for Psychological and Social Factors at Work (QPS Nordic).	N=469 Healthcare personell with patient contact	Sweden	Cross-sectional survey		[93]

Resilience	Stress of conscience	-0.118 (p<=0.01)	Beta - multiple regression analysis	Stress of conscience questionnaire (SCQ), PCQ, General Nordic Questionnaire for Psychological and Social Factors at Work (QPS Nordic).	N=469 Healthcare personell with patient contact	Sweden	Cross-sectional survey		[93]
Working environment : Physical work-related stressors	Stress	n/a	qualitative literature review	n/a	N=n/a, nurses	n/a	Qualitative literature review		[58]
Interpersona l relationships : conflicts with co-workers	Stress	n/a	qualitative literature review	n/a	N=n/a, nurses	n/a	Qualitative literature review		[58]
Interpersona l relationships : lack of staff support	Stress	n/a	qualitative literature review	n/a	N=n/a, nurses	n/a	Qualitative literature review		[58]
Interpersona l relationships : lack of social support from colleagues and superiors	Stress	n/a	qualitative literature review	n/a	N=n/a, nurses	n/a	Qualitative literature review		[58]
Interpersona l relationships : less satisfaction	Stress	n/a	qualitative literature review	n/a	N=n/a, nurses	n/a	Qualitative literature review		[58]

with the head nurses									
Interpersonal relationships: lack of understanding and support from their managers	Stress	n/a	qualitative literature review	n/a	N=n/a, nurses	n/a	Qualitative literature review		[58]
Nature of nursing: Working hours	Stress	n/a	qualitative literature review	n/a	N=n/a, nurses	n/a	Qualitative literature review		[58]
Nature of nursing: Working conditions	Stress	n/a	qualitative literature review	n/a	N=n/a, nurses	n/a	Qualitative literature review		[58]
Nature of nursing: Understaffing	Stress	n/a	qualitative literature review	n/a	N=n/a, nurses	n/a	Qualitative literature review		[58]
Nature of nursing: suffering and death of patients	Stress	n/a	qualitative literature review	n/a	N=n/a, nurses	n/a	Qualitative literature review		[58]
Nature of nursing: Risk of infection	Stress	n/a	qualitative literature review	n/a	N=n/a, nurses	n/a	Qualitative literature review		[58]
Nature of nursing: Risk when handling chemicals	Stress	n/a	qualitative literature review	n/a	N=n/a, nurses	n/a	Qualitative literature review		[58]

Organisational factors: increased job demand	Stress	n/a	qualitative literature review	n/a	N=n/a, nurses	n/a	Qualitative literature review		[58]
Organisational factors: patients' physical/psychological status	Stress	n/a	qualitative literature review	n/a	N=n/a, nurses	n/a	Qualitative literature review		[58]
Organisational factors: use of sophisticated technologies	Stress	n/a	qualitative literature review	n/a	N=n/a, nurses	n/a	Qualitative literature review		[58]
Organisational factors:competition among hospitals	Stress	n/a	qualitative literature review	n/a	N=n/a, nurses	n/a	Qualitative literature review		[58]
Organisational factors: nursing shortage	Stress	n/a	qualitative literature review	n/a	N=n/a, nurses	n/a	Qualitative literature review		[58]
Organisational factors: work overload	Stress	n/a	qualitative literature review	n/a	N=n/a, nurses	n/a	Qualitative literature review		[58]
Organisational factors: lack of task autonomy	Stress	n/a	qualitative literature review	n/a	N=n/a, nurses	n/a	Qualitative literature review		[58]
Organisational factors: lack of feedback	Stress	n/a	qualitative literature review	n/a	N=n/a, nurses	n/a	Qualitative literature review		[58]

Organisational factors: reduced advancement opportunities	Stress	n/a	qualitative literature review	n/a	N=n/a, nurses	n/a	Qualitative literature review		[58]
Occupational factors: workload	Stress	n/a	qualitative literature review	n/a	N=n/a, nurses	n/a	Qualitative literature review		[58]
Occupational factors: responsibility	Stress	n/a	qualitative literature review	n/a	N=n/a, nurses	n/a	Qualitative literature review		[58]
Role characteristics: lack of clarity concerning the employee's targets and duties	Stress	n/a	qualitative literature review	n/a	N=n/a, nurses	n/a	Qualitative literature review		[58]
Role characteristics: conflict among professional roles	Stress	n/a	qualitative literature review	n/a	N=n/a, nurses	n/a	Qualitative literature review		[58]
Role characteristics: overly complex/ambigious/unclear role	Stress	n/a	qualitative literature review	n/a	N=n/a, nurses	n/a	Qualitative literature review		[58]
Role characteristics: highly demanding	Stress	n/a	qualitative literature review	n/a	N=n/a, nurses	n/a	Qualitative literature review		[58]

in terms of competence									
Role characteristics: poor structural means to deal with demand	Stress	n/a	qualitative literature review	n/a	N=n/a, nurses	n/a	Qualitative literature review		[58]
Role characteristics: lack of opportunities to practice the professional role (conflict between expectations of their professional role and reality)	Stress	n/a	qualitative literature review	n/a	N=n/a, nurses	n/a	Qualitative literature review		[58]
Role characteristics: home-work interface	Stress	n/a	qualitative literature review	n/a	N=n/a, nurses	n/a	Qualitative literature review		[58]
Role characteristics: fulfilling others' expectations for the role	Stress	n/a	qualitative literature review	n/a	N=n/a, nurses	n/a	Qualitative literature review		[58]
Role characteristics:	Stress	n/a	qualitative literature review	n/a	N=n/a, nurses	n/a	Qualitative literature review		[58]

conflicting priorities									
Role characteristics: lack of recognition	Stress	n/a	qualitative literature review	n/a	N=n/a, nurses	n/a	Qualitative literature review		[58]
Role characteristics: helping patients with major health problems that are not cognizant of the efforts	Stress	n/a	qualitative literature review	n/a	N=n/a, nurses	n/a	Qualitative literature review		[58]
Individual characteristics: ability to cope with stress factors	Stress	n/a	qualitative literature review	n/a	N=n/a, nurses	n/a	Qualitative literature review		[58]
Individual characteristics: lack of preparation in handling emotional needs of patients	Anxiety	n/a	qualitative literature review	n/a	N=n/a, nurses	n/a	Qualitative literature review		[58]
Individual characteristics: working experience	Stress	n/a	qualitative literature review	n/a	N=n/a, nurses	n/a	Qualitative literature review		[58]
Individual characteristics: education	Stress	n/a	qualitative literature review	n/a	N=n/a, nurses	n/a	Qualitative literature review		[58]
Stress	ability to perform tasks	n/a	qualitative literature review	n/a	N=n/a, nurses	n/a	Qualitative literature review		[58]

Stress	poor decision making	n/a	qualitative literature review	n/a	N=n/a, nurses	n/a	Qualitative literature review		[58]
Stress	Lack of concentratio n	n/a	qualitative literature review	n/a	N=n/a, nurses	n/a	Qualitative literature review		[58]
Stress	Apathy	n/a	qualitative literature review	n/a	N=n/a, nurses	n/a	Qualitative literature review		[58]
Stress	decreased motivation	n/a	qualitative literature review	n/a	N=n/a, nurses	n/a	Qualitative literature review		[58]
Anxiety	jobb performance	n/a	qualitative literature review	n/a	N=n/a, nurses	n/a	Qualitative literature review		[58]
Stress	Anxiety	n/a	qualitative literature review	n/a	N=n/a, nurses	n/a	Qualitative literature review		[58]
Stress	depression	n/a	qualitative literature review	n/a	N=n/a, nurses	n/a	Qualitative literature review		[58]
Stress	insomnia	n/a	qualitative literature review	n/a	N=n/a, nurses	n/a	Qualitative literature review		[58]
Stress	feelings of inadequacy	n/a	qualitative literature review	n/a	N=n/a, nurses	n/a	Qualitative literature review		[58]
Stress	physical health	n/a	qualitative literature review	n/a	N=n/a, nurses	n/a	Qualitative literature review		[58]
Age	Job strain	p=0.024	Students t-test	Job strain in dementia care scale (SDCS), Sense of coherence questionnaire, Person-centred care assessment tool, Person-centred climate questionnaire,	Nurse assistants (N=225), residential care	Sweden	Cross-sectional, descriptive, explorative study		[59]

				Quality of care aspect questionnaire, The creative organisational climat questionnaire, leadership behaviour questionnaire.					
Civil status	Job strain	p=0.023	Chi-squared test	Job strain in dementia care scale (SDCS), Sense of coherence questionnaire, Person-centred care assessment tool, Person-centred climate questionnaire, Quality of care aspect questionnaire, The creative organisational climat questionnaire, leadership behaviour questionnaire.	Nurse assistants (N=225), residential care	Sweden	Cross-sectional, descriptive, explorative study		[59]
Formal education	Job strain	p=0.001	Cramer's V	Job strain in dementia care scale (SDCS), Sense of coherence questionnaire, Person-centred care assessment tool, Person-centred climate questionnaire, Quality of care aspect questionnaire, The creative organisational climat questionnaire, leadership behaviour questionnaire.	Nurse assistants (N=225), residential care	Sweden	Cross-sectional, descriptive, explorative study		[59]

Work experience	Job strain	p=0.003	Mann-Whitney U-test	Job strain in dementia care scale (SDCS), Sense of coherence questionnaire, Person-centred care assessment tool, Person-centred climate questionnaire, Quality of care aspect questionnaire, The creative organisational climat questionnaire, leadership behaviour questionnaire.	Nurse assistants (N=225), residential care	Sweden	Cross-sectional, descriptive, explorative study		[59]
Position/rank	Job strain	p=0.004	Chi-squared test	Job strain in dementia care scale (SDCS), Sense of coherence questionnaire, Person-centred care assessment tool, Person-centred climate questionnaire, Quality of care aspect questionnaire, The creative organisational climat questionnaire, leadership behaviour questionnaire.	Nurse assistants (N=225), residential care	Sweden	Cross-sectional, descriptive, explorative study		[59]
Feeling worried/restless	Job strain	p=0.001	Chi-squared test	Job strain in dementia care scale (SDCS), Sense of coherence questionnaire, Person-centred care assessment tool, Person-centred climate questionnaire, Quality of care aspect	Nurse assistants (N=225), residential care	Sweden	Cross-sectional, descriptive, explorative study		[59]

				questionnaire, The creative organisational climat questionnaire, leadership behaviour questionnaire.					
Physically exhausted after work	Job strain	p=0.005	Chi-squared test	Job strain in dementia care scale (SDCS), Sense of coherence questionnaire, Person-centred care assessment tool, Person-centred climate questionnaire, Quality of care aspect questionnaire, The creative organisational climat questionnaire, leadership behaviour questionnaire.	Nurse assistants (N=225), residential care	Sweden	Cross-sectional, descriptive, explorative study		[59]
Feeling sad and depressed	Job strain	p=0.005	Chi-squared test	Job strain in dementia care scale (SDCS), Sense of coherence questionnaire, Person-centred care assessment tool, Person-centred climate questionnaire, Quality of care aspect questionnaire, The creative organisational climat questionnaire, leadership behaviour questionnaire.	Nurse assistants (N=225), residential care	Sweden	Cross-sectional, descriptive, explorative study		[59]

Sense of coherence	Job strain	p<0.001	Mann-Whitney U-test	Job strain in dementia care scale (SDCS), Sense of coherence questionnaire, Person-centred care assessment tool, Person-centred climate questionnaire, Quality of care aspect questionnaire, The creative organisational climat questionnaire, leadership behaviour questionnaire.	Nurse assistants (N=225), residential care	Sweden	Cross-sectional, descriptive, explorative study		[59]
Organisational and environmental support	Job strain	p<0.001	Mann-Whitney U-test	Job strain in dementia care scale (SDCS), Sense of coherence questionnaire, Person-centred care assessment tool, Person-centred climate questionnaire, Quality of care aspect questionnaire, The creative organisational climat questionnaire, leadership behaviour questionnaire.	Nurse assistants (N=225), residential care	Sweden	Cross-sectional, descriptive, explorative study		[59]
Caring climate	Job strain	p=0.007	Mann-Whitney U-test	Job strain in dementia care scale (SDCS), Sense of coherence questionnaire, Person-centred care assessment tool, Person-centred climate questionnaire, Quality of care aspect	Nurse assistants (N=225), residential care	Sweden	Cross-sectional, descriptive, explorative study		[59]

				questionnaire, The creative organisational climat questionnaire, leadership behaviour questionnaire.					
Quality of care	Job strain	p<0.001	Mann-Whitney U-test	Job strain in dementia care scale (SDCS), Sense of coherence questionnaire, Person-centred care assessment tool, Person-centred climate questionnaire, Quality of care aspect questionnaire, The creative organisational climat questionnaire, leadership behaviour questionnaire.	Nurse assistants (N=225), residential care	Sweden	Cross-sectional, descriptive, explorative study		[59]
Leadership	Job strain	p=0.019	Mann-Whitney U-test	Job strain in dementia care scale (SDCS), Sense of coherence questionnaire, Person-centred care assessment tool, Person-centred climate questionnaire, Quality of care aspect questionnaire, The creative organisational climat questionnaire, leadership behaviour questionnaire.	Nurse assistants (N=225), residential care	Sweden	Cross-sectional, descriptive, explorative study		[59]

Formal education	Stress of conscience	p=0.041	Cramer's V	Job strain in dementia care scale (SDCS), Sense of coherence questionnaire, Person-centred care assessment tool, Person-centred climate questionnaire, Quality of care aspect questionnaire, The creative organisational climat questionnaire, leadership behaviour questionnaire.	Nurse assistants (N=225), residential care	Sweden	Cross-sectional, descriptive, explorative study		[59]
Position/ran k	Stress of conscience	p=0.016	Chi-squared test	Job strain in dementia care scale (SDCS), Sense of coherence questionnaire, Person-centred care assessment tool, Person-centred climate questionnaire, Quality of care aspect questionnaire, The creative organisational climat questionnaire, leadership behaviour questionnaire.	Nurse assistants (N=225), residential care	Sweden	Cross-sectional, descriptive, explorative study		[59]
Shift work	Stress of conscience	p=0.012	Chi-squared test	Job strain in dementia care scale (SDCS), Sense of coherence questionnaire, Person-centred care assessment tool, Person-centred climate questionnaire, Quality of care aspect	Nurse assistants (N=225), residential care	Sweden	Cross-sectional, descriptive, explorative study		[59]

				questionnaire, The creative organisational climat questionnaire, leadership behaviour questionnaire.					
Feeling worried/restless	Stress of conscience	p=0.012	Chi-squared test	Job strain in dementia care scale (SDCS), Sense of coherence questionnaire, Person-centred care assessment tool, Person-centred climate questionnaire, Quality of care aspect questionnaire, The creative organisational climat questionnaire, leadership behaviour questionnaire.	Nurse assistants (N=225), residential care	Sweden	Cross-sectional, descriptive, explorative study		[59]
Physically exhausted after work	Stress of conscience	p=0.008	Chi-squared test	Job strain in dementia care scale (SDCS), Sense of coherence questionnaire, Person-centred care assessment tool, Person-centred climate questionnaire, Quality of care aspect questionnaire, The creative organisational climat questionnaire, leadership behaviour questionnaire.	Nurse assistants (N=225), residential care	Sweden	Cross-sectional, descriptive, explorative study		[59]

Sleeping problems	Stress of conscience	p=0.018	Chi-squared test	Job strain in dementia care scale (SDCS), Sense of coherence questionnaire, Person-centred care assessment tool, Person-centred climate questionnaire, Quality of care aspect questionnaire, The creative organisational climat questionnaire, leadership behaviour questionnaire.	Nurse assistants (N=225), residential care	Sweden	Cross-sectional, descriptive, explorative study		[59]
Feeling sad and depressed	Stress of conscience	p=0.003	Chi-squared test	Job strain in dementia care scale (SDCS), Sense of coherence questionnaire, Person-centred care assessment tool, Person-centred climate questionnaire, Quality of care aspect questionnaire, The creative organisational climat questionnaire, leadership behaviour questionnaire.	Nurse assistants (N=225), residential care	Sweden	Cross-sectional, descriptive, explorative study		[59]
Sense of coherence	Stress of conscience	p=0.012	Mann-Whitney U-test	Job strain in dementia care scale (SDCS), Sense of coherence questionnaire, Person-centred care assessment tool, Person-centred climate questionnaire, Quality of care aspect	Nurse assistants (N=225), residential care	Sweden	Cross-sectional, descriptive, explorative study		[59]

				questionnaire, The creative organisational climat questionnaire, leadership behaviour questionnaire.					
Personalised care provision	Stress of conscience	p=0.015	Mann-Whitney U-test	Job strain in dementia care scale (SDCS), Sense of coherence questionnaire, Person-centred care assessment tool, Person-centred climate questionnaire, Quality of care aspect questionnaire, The creative organisational climat questionnaire, leadership behaviour questionnaire.	Nurse assistants (N=225), residential care	Sweden	Cross-sectional, descriptive, explorative study		[59]
Organisational and environmental support	Stress of conscience	p<0.001	Mann-Whitney U-test	Job strain in dementia care scale (SDCS), Sense of coherence questionnaire, Person-centred care assessment tool, Person-centred climate questionnaire, Quality of care aspect questionnaire, The creative organisational climat questionnaire, leadership behaviour questionnaire.	Nurse assistants (N=225), residential care	Sweden	Cross-sectional, descriptive, explorative study		[59]

Caring climate	Stress of conscience	p=0.001	Mann-Whitney U-test	Job strain in dementia care scale (SDCS), Sense of coherence questionnaire, Person-centred care assessment tool, Person-centred climate questionnaire, Quality of care aspect questionnaire, The creative organisational climat questionnaire, leadership behaviour questionnaire.	Nurse assistants (N=225), residential care	Sweden	Cross-sectional, descriptive, explorative study		[59]
Quality of care	Stress of conscience	p=0.007	Mann-Whitney U-test	Job strain in dementia care scale (SDCS), Sense of coherence questionnaire, Person-centred care assessment tool, Person-centred climate questionnaire, Quality of care aspect questionnaire, The creative organisational climat questionnaire, leadership behaviour questionnaire.	Nurse assistants (N=225), residential care	Sweden	Cross-sectional, descriptive, explorative study		[59]
Leadership	Stress of conscience	p=0.006	Mann-Whitney U-test	Job strain in dementia care scale (SDCS), Sense of coherence questionnaire, Person-centred care assessment tool, Person-centred climate questionnaire, Quality of care aspect	Nurse assistants (N=225), residential care	Sweden	Cross-sectional, descriptive, explorative study		[59]

				questionnaire, The creative organisational climat questionnaire, leadership behaviour questionnaire.					
Cumpolsury school only (1)	job strain	Beta: -0.19 (p=0.007)	multiple linear regression	Job strain in dementia care scale (SDCS), Sense of coherence questionnaire, Person-centred care assessment tool, Person-centred climate questionnaire, Quality of care aspect questionnaire, The creative organisational climat questionnaire, leadership behaviour questionnaire.	Nurse assistants (N=225), residential care	Sweden	Cross-sectional, descriptive, explorative study		[59]
Shift work: day (1), night (0)	job strain	Beta: 0.18 (p=0.011)	multiple linear regression	Job strain in dementia care scale (SDCS), Sense of coherence questionnaire, Person-centred care assessment tool, Person-centred climate questionnaire, Quality of care aspect questionnaire, The creative organisational climat questionnaire, leadership behaviour questionnaire.	Nurse assistants (N=225), residential care	Sweden	Cross-sectional, descriptive, explorative study		[59]

Dementia specific unit (1), general residential care unit (0)	job strain	Beta: 0.17 (p=0.015)	multiple linear regression	Job strain in dementia care scale (SDCS), Sense of coherence questionnaire, Person-centred care assessment tool, Person-centred climate questionnaire, Quality of care aspect questionnaire, The creative organisational climat questionnaire, leadership behaviour questionnaire.	Nurse assistants (N=225), residential care	Sweden	Cross-sectional, descriptive, explorative study		[59]
Feeling worried and restless: Sometimes-very often (1), seldom-never (0)	job strain	Beta: 0.25 (p<0.001)	multiple linear regression	Job strain in dementia care scale (SDCS), Sense of coherence questionnaire, Person-centred care assessment tool, Person-centred climate questionnaire, Quality of care aspect questionnaire, The creative organisational climat questionnaire, leadership behaviour questionnaire.	Nurse assistants (N=225), residential care	Sweden	Cross-sectional, descriptive, explorative study		[59]
Organisational and environmental support	job strain	Beta: -0.23 (p=0.003)	multiple linear regression	Job strain in dementia care scale (SDCS), Sense of coherence questionnaire, Person-centred care assessment tool, Person-centred climate questionnaire, Quality of care aspect	Nurse assistants (N=225), residential care	Sweden	Cross-sectional, descriptive, explorative study		[59]

				questionnaire, The creative organisational climat questionnaire, leadership behaviour questionnaire.					
Caring climate	job strain	Beta: -0.22 (p=0.004)	multiple linear regression	Job strain in dementia care scale (SDCS), Sense of coherence questionnaire, Person-centred care assessment tool, Person-centred climate questionnaire, Quality of care aspect questionnaire, The creative organisational climat questionnaire, leadership behaviour questionnaire.	Nurse assistants (N=225), residential care	Sweden	Cross-sectional, descriptive, explorative study		[59]
Cumpolsury school only (1)	F1: frustrated empathy	Beta: -0.22 (p=0.001)	multiple linear regression	Job strain in dementia care scale (SDCS), Sense of coherence questionnaire, Person-centred care assessment tool, Person-centred climate questionnaire, Quality of care aspect questionnaire, The creative organisational climat questionnaire, leadership behaviour questionnaire.	Nurse assistants (N=225), residential care	Sweden	Cross-sectional, descriptive, explorative study		[59]

Position: LPN (licensed practicing nurse; 1), nurse's aide (0)	F1: frustrated empathy	Beta: -0.14 (p=0.049)	multiple linear regression	Job strain in dementia care scale (SDCS), Sense of coherence questionnaire, Person-centred care assessment tool, Person-centred climate questionnaire, Quality of care aspect questionnaire, The creative organisational climat questionnaire, leadership behaviour questionnaire.	Nurse assistants (N=225), residential care	Sweden	Cross-sectional, descriptive, explorative study		[59]
Shift work: day (1), night (0)	F1: frustrated empathy	Beta: 0.15 (p=0.03)	multiple linear regression	Job strain in dementia care scale (SDCS), Sense of coherence questionnaire, Person-centred care assessment tool, Person-centred climate questionnaire, Quality of care aspect questionnaire, The creative organisational climat questionnaire, leadership behaviour questionnaire.	Nurse assistants (N=225), residential care	Sweden	Cross-sectional, descriptive, explorative study		[59]
Dementia specific unit (1), general residential care unit (0)	F1: frustrated empathy	Beta: 0.19 (p=0.007)	multiple linear regression	Job strain in dementia care scale (SDCS), Sense of coherence questionnaire, Person-centred care assessment tool, Person-centred climate questionnaire, Quality of care aspect	Nurse assistants (N=225), residential care	Sweden	Cross-sectional, descriptive, explorative study		[59]

				questionnaire, The creative organisational climat questionnaire, leadership behaviour questionnaire.					
Feeling worried and restless: Sometimes-very often (1), seldom-never (0)	F1: frustrated empathy	Beta: 0.31 (p<0.001)	multiple linear regression	Job strain in dementia care scale (SDCS), Sense of coherence questionnaire, Person-centred care assessment tool, Person-centred climate questionnaire, Quality of care aspect questionnaire, The creative organisational climat questionnaire, leadership behaviour questionnaire.	Nurse assistants (N=225), residential care	Sweden	Cross-sectional, descriptive, explorative study		[59]
Caring climate	F1: frustrated empathy	Beta: -0.29 (p<0.001)	multiple linear regression	Job strain in dementia care scale (SDCS), Sense of coherence questionnaire, Person-centred care assessment tool, Person-centred climate questionnaire, Quality of care aspect questionnaire, The creative organisational climat questionnaire, leadership behaviour questionnaire.	Nurse assistants (N=225), residential care	Sweden	Cross-sectional, descriptive, explorative study		[59]

Sense of coherence	F2: difficulties understandin g and intepreting	Beta: -0.24 (p=0.001)	multiple linear regression	Job strain in dementia care scale (SDCS), Sense of coherence questionnaire, Person-centred care assessment tool, Person-centred climate questionnaire, Quality of care aspect questionnaire, The creative organisational climat questionnaire, leadership behaviour questionnaire.	Nurse assistants (N=225), residential care	Sweden	Cross-sectional, descriptive, explorative study		[59]
Personalised care provision	F2: difficulties understandin g and intepreting	Beta: 0.17 (p=0.041)	multiple linear regression	Job strain in dementia care scale (SDCS), Sense of coherence questionnaire, Person-centred care assessment tool, Person-centred climate questionnaire, Quality of care aspect questionnaire, The creative organisational climat questionnaire, leadership behaviour questionnaire.	Nurse assistants (N=225), residential care	Sweden	Cross-sectional, descriptive, explorative study		[59]
Caring climate	F2: difficulties understandin g and intepreting	Beta: -0.19 (p=0.026)	multiple linear regression	Job strain in dementia care scale (SDCS), Sense of coherence questionnaire, Person-centred care assessment tool, Person-centred climate questionnaire, Quality of care aspect	Nurse assistants (N=225), residential care	Sweden	Cross-sectional, descriptive, explorative study		[59]

				questionnaire, The creative organisational climat questionnaire, leadership behaviour questionnaire.					
quality of care	F2: difficulties understanding and intepreting	Beta: -0.27 (p=0.001)	multiple linear regression	Job strain in dementia care scale (SDCS), Sense of coherence questionnaire, Person-centred care assessment tool, Person-centred climate questionnaire, Quality of care aspect questionnaire, The creative organisational climat questionnaire, leadership behaviour questionnaire.	Nurse assistants (N=225), residential care	Sweden	Cross-sectional, descriptive, explorative study		[59]
Cumpolsury school only (1)	F3: Balancing competing needs	Beta: -0.18 (p=0.011)	multiple linear regression	Job strain in dementia care scale (SDCS), Sense of coherence questionnaire, Person-centred care assessment tool, Person-centred climate questionnaire, Quality of care aspect questionnaire, The creative organisational climat questionnaire, leadership behaviour questionnaire.	Nurse assistants (N=225), residential care	Sweden	Cross-sectional, descriptive, explorative study		[59]

Physically exhausted after work: Sometimes-very often (1), seldom-never (0)	F3: Balancing competing needs	Beta: 0.16 (p=0.022)	multiple linear regression	Job strain in dementia care scale (SDCS), Sense of coherence questionnaire, Person-centred care assessment tool, Person-centred climate questionnaire, Quality of care aspect questionnaire, The creative organisational climat questionnaire, leadership behaviour questionnaire.	Nurse assistants (N=225), residential care	Sweden	Cross-sectional, descriptive, explorative study		[59]
Sleeping problems: sometimes-very often (1), seldom-never (0)	F3: Balancing competing needs	Beta: -0.16 (p=0.031)	multiple linear regression	Job strain in dementia care scale (SDCS), Sense of coherence questionnaire, Person-centred care assessment tool, Person-centred climate questionnaire, Quality of care aspect questionnaire, The creative organisational climat questionnaire, leadership behaviour questionnaire.	Nurse assistants (N=225), residential care	Sweden	Cross-sectional, descriptive, explorative study		[59]
Feeling worried and restless: Sometimes-very often (1), seldom-never (0)	F3: Balancing competing needs	Beta: 0.22 (p=0.004)	multiple linear regression	Job strain in dementia care scale (SDCS), Sense of coherence questionnaire, Person-centred care assessment tool, Person-centred climate questionnaire, Quality of care aspect	Nurse assistants (N=225), residential care	Sweden	Cross-sectional, descriptive, explorative study		[59]

				questionnaire, The creative organisational climat questionnaire, leadership behaviour questionnaire.					
Organisational and environmental support	F3: Balancing competing needs	Beta: -0.33 (p<0.001)	multiple linear regression	Job strain in dementia care scale (SDCS), Sense of coherence questionnaire, Person-centred care assessment tool, Person-centred climate questionnaire, Quality of care aspect questionnaire, The creative organisational climat questionnaire, leadership behaviour questionnaire.	Nurse assistants (N=225), residential care	Sweden	Cross-sectional, descriptive, explorative study		[59]
The general work climate	F3: Balancing competing needs	Beta: 0.24 (p=0.002)	multiple linear regression	Job strain in dementia care scale (SDCS), Sense of coherence questionnaire, Person-centred care assessment tool, Person-centred climate questionnaire, Quality of care aspect questionnaire, The creative organisational climat questionnaire, leadership behaviour questionnaire.	Nurse assistants (N=225), residential care	Sweden	Cross-sectional, descriptive, explorative study		[59]

Leadership	F3: Balancing competing needs	Beta: -0.22 (p=.005)	multiple linear regression	Job strain in dementia care scale (SDCS), Sense of coherence questionnaire, Person-centred care assessment tool, Person-centred climate questionnaire, Quality of care aspect questionnaire, The creative organisational climat questionnaire, leadership behaviour questionnaire.	Nurse assistants (N=225), residential care	Sweden	Cross-sectional, descriptive, explorative study		[59]
Civil status: married/partner (1), single (0)	F4: Balancing emotional involvement	Beta: -0.15 (p=0.022)	multiple linear regression	Job strain in dementia care scale (SDCS), Sense of coherence questionnaire, Person-centred care assessment tool, Person-centred climate questionnaire, Quality of care aspect questionnaire, The creative organisational climat questionnaire, leadership behaviour questionnaire.	Nurse assistants (N=225), residential care	Sweden	Cross-sectional, descriptive, explorative study		[59]
Dementia specific unit (1), general residential care unit (0)	F4: Balancing emotional involvement	Beta: 0.16 (p=0.022)	multiple linear regression	Job strain in dementia care scale (SDCS), Sense of coherence questionnaire, Person-centred care assessment tool, Person-centred climate questionnaire, Quality of care aspect	Nurse assistants (N=225), residential care	Sweden	Cross-sectional, descriptive, explorative study		[59]

				questionnaire, The creative organisational climat questionnaire, leadership behaviour questionnaire.					
Feeling worried and restless: Sometimes-very often (1), seldom-never (0)	F4: Balancing emotional involvement	Beta: 0.16 (p=0.036)	multiple linear regression	Job strain in dementia care scale (SDCS), Sense of coherence questionnaire, Person-centred care assessment tool, Person-centred climate questionnaire, Quality of care aspect questionnaire, The creative organisational climat questionnaire, leadership behaviour questionnaire.	Nurse assistants (N=225), residential care	Sweden	Cross-sectional, descriptive, explorative study		[59]
Organisational and environmental support	F4: Balancing emotional involvement	Beta: -0.20 (p=0.006)	multiple linear regression	Job strain in dementia care scale (SDCS), Sense of coherence questionnaire, Person-centred care assessment tool, Person-centred climate questionnaire, Quality of care aspect questionnaire, The creative organisational climat questionnaire, leadership behaviour questionnaire.	Nurse assistants (N=225), residential care	Sweden	Cross-sectional, descriptive, explorative study		[59]

Cumpolsury school only (1)	F5: Lack of recognition	Beta: -0.27 (p<0.001)	multiple linear regression	Job strain in dementia care scale (SDCS), Sense of coherence questionnaire, Person-centred care assessment tool, Person-centred climate questionnaire, Quality of care aspect questionnaire, The creative organisational climat questionnaire, leadership behaviour questionnaire.	Nurse assistants (N=225), residential care	Sweden	Cross-sectional, descriptive, explorative study		[59]
Position: LPN (licensed practicing nurse; 1), nurse's aide (0)	F5: Lack of recognition	Beta: -0.15 (p=0.02)	multiple linear regression	Job strain in dementia care scale (SDCS), Sense of coherence questionnaire, Person-centred care assessment tool, Person-centred climate questionnaire, Quality of care aspect questionnaire, The creative organisational climat questionnaire, leadership behaviour questionnaire.	Nurse assistants (N=225), residential care	Sweden	Cross-sectional, descriptive, explorative study		[59]
Shift work: day (1), night (0)	F5: Lack of recognition	Beta: 0.14 (p=0.027)	multiple linear regression	Job strain in dementia care scale (SDCS), Sense of coherence questionnaire, Person-centred care assessment tool, Person-centred climate questionnaire, Quality of care aspect	Nurse assistants (N=225), residential care	Sweden	Cross-sectional, descriptive, explorative study		[59]

				questionnaire, The creative organisational climat questionnaire, leadership behaviour questionnaire.					
Dementia specific unit (1), general residential care unit (0)	F5: Lack of recognition	Beta: 0.14 (p=0.026)	multiple linear regression	Job strain in dementia care scale (SDCS), Sense of coherence questionnaire, Person-centred care assessment tool, Person-centred climate questionnaire, Quality of care aspect questionnaire, The creative organisational climat questionnaire, leadership behaviour questionnaire.	Nurse assistants (N=225), residential care	Sweden	Cross-sectional, descriptive, explorative study		[59]
Physically exhausted after work: Sometimes-very often (1), seldom-never (0)	F5: Lack of recognition	Beta: 0.16 (p=0.011)	multiple linear regression	Job strain in dementia care scale (SDCS), Sense of coherence questionnaire, Person-centred care assessment tool, Person-centred climate questionnaire, Quality of care aspect questionnaire, The creative organisational climat questionnaire, leadership behaviour questionnaire.	Nurse assistants (N=225), residential care	Sweden	Cross-sectional, descriptive, explorative study		[59]

Feeling worried and restless: Sometimes-very often (1), seldom-never (0)	F5: Lack of recognition	Beta: 0.15 (p=0.019)	multiple linear regression	Job strain in dementia care scale (SDCS), Sense of coherence questionnaire, Person-centred care assessment tool, Person-centred climate questionnaire, Quality of care aspect questionnaire, The creative organisational climat questionnaire, leadership behaviour questionnaire.	Nurse assistants (N=225), residential care	Sweden	Cross-sectional, descriptive, explorative study		[59]
Personalised care provision	F5: Lack of recognition	Beta: 0.30 (p<0.001)	multiple linear regression	Job strain in dementia care scale (SDCS), Sense of coherence questionnaire, Person-centred care assessment tool, Person-centred climate questionnaire, Quality of care aspect questionnaire, The creative organisational climat questionnaire, leadership behaviour questionnaire.	Nurse assistants (N=225), residential care	Sweden	Cross-sectional, descriptive, explorative study		[59]
Organisational and environmental support	F5: Lack of recognition	Beta: -0.36 (p<0.001)	multiple linear regression	Job strain in dementia care scale (SDCS), Sense of coherence questionnaire, Person-centred care assessment tool, Person-centred climate questionnaire, Quality of care aspect	Nurse assistants (N=225), residential care	Sweden	Cross-sectional, descriptive, explorative study		[59]

				questionnaire, The creative organisational climat questionnaire, leadership behaviour questionnaire.					
Leadership	F5: Lack of recognition	Beta: -0.34 (p<0.001)	multiple linear regression	Job strain in dementia care scale (SDCS), Sense of coherence questionnaire, Person-centred care assessment tool, Person-centred climate questionnaire, Quality of care aspect questionnaire, The creative organisational climat questionnaire, leadership behaviour questionnaire.	Nurse assistants (N=225), residential care	Sweden	Cross-sectional, descriptive, explorative study		[59]
Frustrated empathy	Strain	n/a	factor analysis	Job strain in dementia care scale (SDCS)	N=346 dementia caregivers	Sweden	Instrument development	SDCS ; Factors intercorrelated	[51]
Difficulties understanding and interpreting	Strain	n/a	factor analysis	Job strain in dementia care scale (SDCS)	N=346 dementia caregivers	Sweden	Instrument development	SDCS ; Factors intercorrelated	[51]
balancing competing needs	Strain	n/a	factor analysis	Job strain in dementia care scale (SDCS)	N=346 dementia caregivers	Sweden	Instrument development	SDCS ; Factors intercorrelated	[51]
balancing emotional involvement	Strain	n/a	factor analysis	Job strain in dementia care scale (SDCS)	N=346 dementia caregivers	Sweden	Instrument development	SDCS ; Factors intercorrelated	[51]
lack of recognition	Strain	n/a	factor analysis	Job strain in dementia care scale (SDCS)	N=346 dementia caregivers	Sweden	Instrument development	SDCS ; Factors intercorrelated	[51]

Age	total stress	p=0.042	correlation analysis	Irish Nursing Association instrument	N=206 nurses in and outside hospital setting	Iceland	cross-sectional questionnaire survey study		[62]
Unscheduled work	total stress	p<0.001	correlation analysis	Irish Nursing Association instrument	N=206 nurses in and outside hospital setting	Iceland	cross-sectional questionnaire survey study		[62]
Years of total experience	total stress	p=0.041	correlation analysis	Irish Nursing Association instrument	N=206 nurses in and outside hospital setting	Iceland	cross-sectional questionnaire survey study		[62]
Job satisfaction	total stress	p=0.002	correlation analysis	Irish Nursing Association instrument	N=206 nurses in and outside hospital setting	Iceland	cross-sectional questionnaire survey study		[62]
satisfaction from head nurse	total stress	p<0.001	correlation analysis	Irish Nursing Association instrument	N=206 nurses in and outside hospital setting	Iceland	cross-sectional questionnaire survey study		[62]
satisfaction from nurse directors	total stress	p<0.001	correlation analysis	Irish Nursing Association instrument	N=206 nurses in and outside hospital setting	Iceland	cross-sectional questionnaire survey study		[62]
satisfaction from co-workers	total stress	p=0.007	correlation analysis	Irish Nursing Association instrument	N=206 nurses in and outside hospital setting	Iceland	cross-sectional questionnaire survey study		[62]
support from head nurses	total stress	p=0.031	correlation analysis	Irish Nursing Association instrument	N=206 nurses in and outside hospital setting	Iceland	cross-sectional questionnaire survey study		[62]
support from nurse directors	total stress	p=0.029	correlation analysis	Irish Nursing Association instrument	N=206 nurses in and outside hospital setting	Iceland	cross-sectional questionnaire survey study		[62]
opportunity to practice different aspects of the professional role	frequency of stress	Beta: -0.243	correlation analysis	Irish Nursing Association instrument	N=206 nurses in and outside hospital setting	Iceland	cross-sectional questionnaire survey study		[62]
Unscheduled work	frequency of stress	Beta: 0.226	correlation analysis	Irish Nursing Association instrument	N=206 nurses in and outside hospital setting	Iceland	cross-sectional questionnaire survey study		[62]

satisfaction with head nurses	frequency of stress	Beta: 0.233	correlation analysis	Irish Nursing Association instrument	N=206 nurses in and outside hospital setting	Iceland	cross-sectional questionnaire survey study		[62]
Years of work experience	frequency of stress	Beta: -0.170	correlation analysis	Irish Nursing Association instrument	N=206 nurses in and outside hospital setting	Iceland	cross-sectional questionnaire survey study		[62]
Quality of job content	intrinsic work motivation	Beta: 0.28	significant relations, structural equation modelling with LISREL	Multi-item questionnaires.	N=175 nurses, general hospital	The Netherla nds	cross-sectional questionnaire survey study		[94]
Mental work overload	emotional exhaustion	Beta: 0.38	significant relations, structural equation modelling with LISREL	Multi-item questionnaires.	N=175 nurses, general hospital	The Netherla nds	cross-sectional questionnaire survey study		[94]
social support	emotional exhaustion	Beta: -0.26	significant relations, structural equation modelling with LISREL	Multi-item questionnaires.	N=175 nurses, general hospital	The Netherla nds	cross-sectional questionnaire survey study		[94]
umet career expectations	turnover intention	Beta: 0.51	significant relations, structural equation modelling with LISREL	Multi-item questionnaires.	N=175 nurses, general hospital	The Netherla nds	cross-sectional questionnaire survey study		[94]
instrinsic work motivation	turnover intention	n/a	significant relations, structural equation	Multi-item questionnaires.	N=175 nurses, general hospital	The Netherla nds	cross-sectional questionnaire survey study		[94]

			modelling with LISREL						
intrinsic work motivation	emotional exhaustion	n/a	significant relations, structural equation modelling with LISREL	Multi-item questionnaires.	N=175 nurses, general hospital	The Netherla nds	cross-sectional questionnaire survey study		[94]
emotional exhaustion	intrinsic work motivation	n/a	significant relations, structural equation modelling with LISREL	Multi-item questionnaires.	N=175 nurses, general hospital	The Netherla nds	cross-sectional questionnaire survey study		[94]
emotional exhaustion	turnover intention	n/a	significant relations, structural equation modelling with LISREL	Multi-item questionnaires.	N=175 nurses, general hospital	The Netherla nds	cross-sectional questionnaire survey study		[94]
turnover intention	emotional exhaustion	n/a	significant relations, structural equation modelling with LISREL	Multi-item questionnaires.	N=175 nurses, general hospital	The Netherla nds	cross-sectional questionnaire survey study		[94]
turnover intention	intrinsic work motivation	n/a	significant relations, structural equation modelling with LISREL	Multi-item questionnaires.	N=175 nurses, general hospital	The Netherla nds	cross-sectional questionnaire survey study		[94]

mastery of work	stress	n/a	multiple analyses	QPS Nordic	N=197 care staff, in 13 dementia wards, at 4 nursing homes	Norway	cross-sectional questionnaire survey study		[95]
control at work	stress	n/a	multiple analyses	QPS Nordic	N=197 care staff, in 13 dementia wards, at 4 nursing homes	Norway	cross-sectional questionnaire survey study		[95]
control at work	stress	n/a	multiple analyses	QPS Nordic	N=197 care staff, in 13 dementia wards, at 4 nursing homes	Norway	cross-sectional questionnaire survey study		[95]
mastery of work	stress	n/a	multiple analyses	QPS Nordic	N=197 care staff, in 13 dementia wards, at 4 nursing homes	Norway	cross-sectional questionnaire survey study		[95]
Leadership	stress	n/a	multiple analyses	QPS Nordic	N=197 care staff, in 13 dementia wards, at 4 nursing homes	Norway	cross-sectional questionnaire survey study		[95]
physical function of care recipient (Kartz index)	caregiver strain	-0.23	Significant relations, path analysis	Care giver strain index (CSI), resilience levels (RES).	N=230 Alzheimer's care givers	Malaysia	cross-sectional questionnaire survey study		[96]
employment status	resilience	0.22	Significant relations, path analysis	Care giver strain index (CSI), resilience levels (RES).	N=230 Alzheimer's care givers	Malaysia	cross-sectional questionnaire survey study		[96]
Gender	resilience	-0.25	Significant relations, path analysis	Care giver strain index (CSI), resilience levels (RES).	N=230 Alzheimer's care givers	Malaysia	cross-sectional questionnaire survey study		[96]
Kinship	caregiver strain	0.18	Significant relations, path analysis	Care giver strain index (CSI), resilience levels (RES).	N=230 Alzheimer's care givers	Malaysia	cross-sectional questionnaire survey study		[96]

Years of care	caregiver strain	0.17	Significant relations, path analysis	Care giver strain index (CSI), resilience levels (RES).	N=230 Alzheimer's care givers	Malaysia	cross-sectional questionnaire survey study		[96]
Resilience	caregiver strain	-0.37	Significant relations, path analysis	Care giver strain index (CSI), resilience levels (RES).	N=230 Alzheimer's care givers	Malaysia	cross-sectional questionnaire survey study		[96]
quantitative demands	workload	1	significant relations, structural equation modelling with LISREL	QPS Nordic	n/a	n/a	n/a	QPS Nordic user manual	[57]
decision demands	workload	0.6	significant relations, structural equation modelling with LISREL	QPS Nordic	n/a	n/a	n/a	QPS Nordic user manual	[57]
decision demands	complexity of work	0.64	significant relations, structural equation modelling with LISREL	QPS Nordic	n/a	n/a	n/a	QPS Nordic user manual	[57]
learning demands	complexity of work	1	significant relations, structural equation modelling with LISREL	QPS Nordic	n/a	n/a	n/a	QPS Nordic user manual	[57]
empowering leadership	complexity of work	0.6	significant relations,	QPS Nordic	n/a	n/a	n/a	QPS Nordic user manual	[57]

			structural equation modelling with LISREL						
empowering leadership	quality of supervision	0.85	significant relations, structural equation modelling with LISREL	QPS Nordic	n/a	n/a	n/a	QPS Nordic user manual	[57]
right leadership	quality of supervision	0.91	significant relations, structural equation modelling with LISREL	QPS Nordic	n/a	n/a	n/a	QPS Nordic user manual	[57]
support from superior	quality of supervision	1	significant relations, structural equation modelling with LISREL	QPS Nordic	n/a	n/a	n/a	QPS Nordic user manual	[57]
workload	role conflict	0.78	significant relations, structural equation modelling with LISREL	QPS Nordic	n/a	n/a	n/a	QPS Nordic user manual	[57]
complexity of work	positive challenge at work	0.8	significant relations, structural equation modelling	QPS Nordic	n/a	n/a	n/a	QPS Nordic user manual	[57]

			with LISREL						
complexity of work	control of decision	0.61	significant relations, structural equation modelling with LISREL	QPS Nordic	n/a	n/a	n/a	QPS Nordic user manual	[57]
quality of supervision	role conflict	-0.36	significant relations, structural equation modelling with LISREL	QPS Nordic	n/a	n/a	n/a	QPS Nordic user manual	[57]
quality of supervision	control of decision	0.46	significant relations, structural equation modelling with LISREL	QPS Nordic	n/a	n/a	n/a	QPS Nordic user manual	[57]
quality of supervision	role clarity	0.53	significant relations, structural equation modelling with LISREL	QPS Nordic	n/a	n/a	n/a	QPS Nordic user manual	[57]
quality of supervision	social climate	0.55	significant relations, structural equation modelling with LISREL	QPS Nordic	n/a	n/a	n/a	QPS Nordic user manual	[57]

quality of supervision	support from coworkers	0.55	significant relations, structural equation modelling with LISREL	QPS Nordic	n/a	n/a	n/a	QPS Nordic user manual	[57]
quality of supervision	human resource primacy	0.77	significant relations, structural equation modelling with LISREL	QPS Nordic	n/a	n/a	n/a	QPS Nordic user manual	[57]
quality of supervision	commitment to organisation	0.27	significant relations, structural equation modelling with LISREL	QPS Nordic	n/a	n/a	n/a	QPS Nordic user manual	[57]
control of decision	role conflict	-0.13	significant relations, structural equation modelling with LISREL	QPS Nordic	n/a	n/a	n/a	QPS Nordic user manual	[57]
control of decision	positive challenge at work	0.27	significant relations, structural equation modelling with LISREL	QPS Nordic	n/a	n/a	n/a	QPS Nordic user manual	[57]
role clarity	positive challenge at work	0.26	significant relations, structural	QPS Nordic	n/a	n/a	n/a	QPS Nordic user manual	[57]

			equation modelling with LISREL						
positive challenge at work	social climate	0.18	significant relations, structural equation modelling with LISREL	QPS Nordic	n/a	n/a	n/a	QPS Nordic user manual	[57]
positive challenge at work	commitment to organisation	0.23	significant relations, structural equation modelling with LISREL	QPS Nordic	n/a	n/a	n/a	QPS Nordic user manual	[57]
support from coworkers	social climate	0.28	significant relations, structural equation modelling with LISREL	QPS Nordic	n/a	n/a	n/a	QPS Nordic user manual	[57]
human resource primacy	commitment to organisation	0.43	significant relations, structural equation modelling with LISREL	QPS Nordic	n/a	n/a	n/a	QPS Nordic user manual	[57]
work centrality	job involvement	n/a	significant relations, structural equation modelling	QPS Nordic	n/a	n/a	n/a	QPS Nordic user manual	[57]

			with LISREL						
commitment to organisation	job involvement	n/a	significant relations, structural equation modelling with LISREL	QPS Nordic	n/a	n/a	n/a	QPS Nordic user manual	[57]
positive challenge at work	job involvement	n/a	significant relations, structural equation modelling with LISREL	QPS Nordic	n/a	n/a	n/a	QPS Nordic user manual	[57]
empowering leadership	job involvement	n/a	significant relations, structural equation modelling with LISREL	QPS Nordic	n/a	n/a	n/a	QPS Nordic user manual	[57]
commitment to organisation	job satisfaction	n/a	significant relations, structural equation modelling with LISREL	QPS Nordic	n/a	n/a	n/a	QPS Nordic user manual	[57]
positive challenge at work	job satisfaction	n/a	significant relations, structural equation modelling with LISREL	QPS Nordic	n/a	n/a	n/a	QPS Nordic user manual	[57]

empowering leadership	job satisfaction	n/a	significant relations, structural equation modelling with LISREL	QPS Nordic	n/a	n/a	n/a	QPS Nordic user manual	[57]
social climate	job satisfaction	n/a	significant relations, structural equation modelling with LISREL	QPS Nordic	n/a	n/a	n/a	QPS Nordic user manual	[57]
role conflict	job satisfaction	negative correlation	significant relations, structural equation modelling with LISREL	QPS Nordic	n/a	n/a	n/a	QPS Nordic user manual	[57]
other leadership and organisation al factors	job satisfaction	medium strength relationship	significant relations, structural equation modelling with LISREL	QPS Nordic	n/a	n/a	n/a	QPS Nordic user manual	[57]
commitment to organisation	emotional exhaustion	negative correlation	significant relations, structural equation modelling with LISREL	QPS Nordic	n/a	n/a	n/a	QPS Nordic user manual	[57]
quantitative demands	emotional exhaustion	n/a	significant relations, structural	QPS Nordic	n/a	n/a	n/a	QPS Nordic user manual	[57]

			equation modelling with LISREL						
role conflict	emotional exhaustion	n/a	significant relations, structural equation modelling with LISREL	QPS Nordic	n/a	n/a	n/a	QPS Nordic user manual	[57]
social climate	emotional exhaustion	negative correlation	significant relations, structural equation modelling with LISREL	QPS Nordic	n/a	n/a	n/a	QPS Nordic user manual	[57]
role conflict	stress symptoms	n/a	significant relations, structural equation modelling with LISREL	QPS Nordic	n/a	n/a	n/a	QPS Nordic user manual	[57]
quantitative demands	stress symptoms	n/a	significant relations, structural equation modelling with LISREL	QPS Nordic	n/a	n/a	n/a	QPS Nordic user manual	[57]
mastery of work	stress symptoms	negative correlation	significant relations, structural equation modelling	QPS Nordic	n/a	n/a	n/a	QPS Nordic user manual	[57]

			with LISREL						
Educational training intervention	Ability to balance needs of family and care recipients	p<=0.05	significanc e testing	Strain in Dementia Care Scale (SDCS), Creative Climate Questionnaire (CCQ)	N=34 baseline, N=30 follow-up, dementia care specialists in home care service	Sweden	Quasi-experimental one-group intervention study		[97]
Educational training intervention	Ability to balance needs between care recipients	p<=0.05	significanc e testing	Strain in Dementia Care Scale (SDCS), Creative Climate Questionnaire (CCQ)	N=34 baseline, N=30 follow-up, dementia care specialists in home care service	Sweden	Quasi-experimental one-group intervention study		[97]
Ability to balance needs	Job strain	p<=0.05	significanc e testing	Strain in Dementia Care Scale (SDCS), Creative Climate Questionnaire (CCQ)	N=34 baseline, N=30 follow-up, dementia care specialists in home care service	Sweden	Quasi-experimental one-group intervention study		[97]
Social skills	trustful care relationship	n/a	qualitative research design through collection of interview data		N=26 registered nurses, in mental healthcare, hospital care, home care, nursing home care.	The Netherla nds	Descriptive qualitative data collection using interviews.		[65]
Making time for patients (listening/e mphaty)	trustful care relationship	n/a	qualitative research design through collection of interview data		N=26 registered nurses, in mental healthcare, hospital care, home care, nursing home care.	The Netherla nds	Descriptive qualitative data collection using interviews.		[65]
Knowledge	quality of patient care	n/a	qualitative research design through		N=26 registered nurses, in mental healthcare, hospital care,	The Netherla nds	Descriptive qualitative data collection		[65]

			collection of interview data		home care, nursing home care.		using interviews.		
Technical skills	quality of patient care	n/a	qualitative research design through collection of interview data		N=26 registered nurses, in mental healthcare, hospital care, home care, nursing home care.	The Netherlands	Descriptive qualitative data collection using interviews.		[65]
communicative capabilities	quality of patient care	n/a	qualitative research design through collection of interview data		N=26 registered nurses, in mental healthcare, hospital care, home care, nursing home care.	The Netherlands	Descriptive qualitative data collection using interviews.		[65]
Continual development and education	quality of patient care	n/a	qualitative research design through collection of interview data		N=26 registered nurses, in mental healthcare, hospital care, home care, nursing home care.	The Netherlands	Descriptive qualitative data collection using interviews.		[65]
experience	coordinate care and priority setting	n/a	qualitative research design through collection of interview data		N=26 registered nurses, in mental healthcare, hospital care, home care, nursing home care.	The Netherlands	Descriptive qualitative data collection using interviews.		[65]

High workload	reduce right and needed care	n/a	qualitative research design through collection of interview data		N=26 registered nurses, in mental healthcare, hospital care, home care, nursing home care.	The Netherlands	Descriptive qualitative data collection using interviews.		[65]
Collaborative working relationships	Sharing information and communication	n/a	qualitative research design through collection of interview data		N=26 registered nurses, in mental healthcare, hospital care, home care, nursing home care.	The Netherlands	Descriptive qualitative data collection using interviews.		[65]
Sharing information and communication	Aligned, uniform care	n/a	qualitative research design through collection of interview data		N=26 registered nurses, in mental healthcare, hospital care, home care, nursing home care.	The Netherlands	Descriptive qualitative data collection using interviews.		[65]
Autonomy	Make decisions to improve process	n/a	qualitative research design through collection of interview data		N=26 registered nurses, in mental healthcare, hospital care, home care, nursing home care.	The Netherlands	Descriptive qualitative data collection using interviews.		[65]
Number of staff	quality of patient care	n/a	qualitative research design through collection of		N=26 registered nurses, in mental healthcare, hospital care, home care,	The Netherlands	Descriptive qualitative data collection using interviews.		[65]

			interview data		nursing home care.				
Control over practice	quality of patient care	n/a	qualitative research design through collection of interview data		N=26 registered nurses, in mental healthcare, hospital care, home care, nursing home care.	The Netherla nds	Descriptive qualitative data collection using interviews.		[65]
Managerial support	quality of patient care	n/a	qualitative research design through collection of interview data		N=26 registered nurses, in mental healthcare, hospital care, home care, nursing home care.	The Netherla nds	Descriptive qualitative data collection using interviews.		[65]
Services controlled by cost	reduce quality of care	n/a	qualitative research design through collection of interview data		N=26 registered nurses, in mental healthcare, hospital care, home care, nursing home care.	The Netherla nds	Descriptive qualitative data collection using interviews.		[65]
Organisation al emphasis on cost-effectiveness	reduce quality of care	n/a	qualitative research design through collection of interview data		N=26 registered nurses, in mental healthcare, hospital care, home care, nursing home care.	The Netherla nds	Descriptive qualitative data collection using interviews.		[65]
increased administrati ve workload	reduce quality of care	n/a	qualitative research design through		N=26 registered nurses, in mental healthcare, hospital care,	The Netherla nds	Descriptive qualitative data collection		[65]

			collection of interview data		home care, nursing home care.		using interviews.		
Client/patient unmet/met needs	quality of care	n/a	Qualitative interview	semi-structured interview	N=33 inteviews in 11 triads (older adults, caregiver, healthcare provider, in health and social services centres	Canada	qualitative semi-structured interview study		[98]
Job demands	job satisfaction	Beta: -0.20, p<0.01	hierarchical multiple regression analysis		N=379 nursing staff, elderly care	Germany	Cross-sectional questionnaire survey		[118]
Job control	job satisfaction	Beta: 0.31, p<0.01	hierarchical multiple regression analysis		N=379 nursing staff, elderly care	Germany	Cross-sectional questionnaire survey		[118]
demand-control interaction	job satisfaction	Beta: 0.12, p<0.01	hierarchical multiple regression analysis		N=379 nursing staff, elderly care	Germany	Cross-sectional questionnaire survey		[118]
Job demands	psychosomatic complaints	Beta: 0.31,p<0.01	hierarchical multiple regression analysis		N=379 nursing staff, elderly care	Germany	Cross-sectional questionnaire survey		[118]
Job control	psychosomatic complaints	Beta: -0.07, p<0.01	hierarchical multiple regression analysis		N=379 nursing staff, elderly care	Germany	Cross-sectional questionnaire survey		[118]
demand-control interaction	psychosomatic complaints	Beta: -0.07, p<0.01	hierarchical multiple regression analysis		N=379 nursing staff, elderly care	Germany	Cross-sectional questionnaire survey		[118]
Job demands	emotional exhaustion	Beta: 0.43, p<0.01	hierarchical multiple		N=379 nursing staff, elderly care	Germany	Cross-sectional		[118]

			regression analysis				questionnaire survey		
Job control	emotional exhaustion	Beta: -0.21, p<0.01	hierarchical multiple regression analysis		N=379 nursing staff, elderly care	Germany	Cross-sectional questionnaire survey		[118]
demand-control interaction	emotional exhaustion	Beta: -0.14, p<0.01	hierarchical multiple regression analysis		N=379 nursing staff, elderly care	Germany	Cross-sectional questionnaire survey		[118]
Organisational context: less favourable vs. more favourable	Missed care	RR: 1.59, 95% CI: 1.34-1.90, p<0.001	Multivariable analysis	Alberta context tool (ACT)	N=4016 care aides, nursing homes	Canada	Cross-sectional questionnaire survey	Organisational context, defined as: leadership, culture, evaluation, formal interactions, informal interactions, structural and electronic resources, social capital, organisational slack in use of time, organisational slack in use of staff, and organisational slack in use of space (ACT: Alberta Context Tool).	[99]
Organisational context: less favourable vs. more favourable	Rushed care	RR: 1.66, 95% CI: 1.38-1.99	Multivariable analysis	Alberta context tool (ACT)	N=4016 care aides, nursing homes	Canada	Cross-sectional questionnaire survey	Organisational context, defined as: leadership, culture, evaluation, formal interactions, informal interactions, structural and electronic resources, social capital, organisational slack in use of time, organisational slack in use of staff, and organisational slack in use of space (ACT: Alberta Context Tool).	[99]
Unbefriended patient/client/resident	Lack of social support	n/a	Qualitative interview	n/a	N=42 Long-term care staff and public guardians, residential care	Canada	Cross-sectional qualitatitive descriptive interview study		[100]
Lack of social support	Lower quality of care	n/a	Qualitative interview	n/a	N=42 Long-term care staff and public guardians, residential care	Canada	Cross-sectional qualitatitive descriptive		[100]

							interview study		
English (native) as first language - No relative to Yes (ref)	Exhaustion	Beta: 0.227, p=0.008	Mixed-effects regression modelling	Alberta context tool (ACT)	N=1194 Care aides, urban nursing homes	Canada	Longitudinal cross-sectional questionnaire survey, TREC survey		[67]
HCA (healthcare assistant) certificate received - No relative to yes (ref)	Exhaustion	Beta: 0.227, p=0.035	Mixed-effects regression modelling	Alberta context tool (ACT)	N=1194 Care aides, urban nursing homes	Canada	Longitudinal cross-sectional questionnaire survey, TREC survey		[67]
Job satisfaction	Exhaustion	Beta: -0.281, p<0.001	Mixed-effects regression modelling	Alberta context tool (ACT)	N=1194 Care aides, urban nursing homes	Canada	Longitudinal cross-sectional questionnaire survey, TREC survey		[67]
Facility bed size: medium (80-120), relative to small (35-79; ref)	Exhaustion	Beta: 0.428. p=0.002	Mixed-effects regression modelling	Alberta context tool (ACT)	N=1194 Care aides, urban nursing homes	Canada	Longitudinal cross-sectional questionnaire survey, TREC survey		[67]
ACT organisation al slack - space	Exhaustion	Beta: -0.0147, p<0.001	Mixed-effects regression modelling	Alberta context tool (ACT)	N=1194 Care aides, urban nursing homes	Canada	Longitudinal cross-sectional questionnaire survey, TREC survey		[67]
ACT organisation al slack - time	Exhaustion	Beta: 0.185, p<0.001	Mixed-effects regression modelling	Alberta context tool (ACT)	N=1194 Care aides, urban nursing homes	Canada	Longitudinal cross-sectional questionnaire survey, TREC survey		[67]

responsive behaviours	Exhaustion	Beta: 0.105, p<0.001	Mixed-effects regression modelling	Alberta context tool (ACT)	N=1194 Care aides, urban nursing homes	Canada	Longitudinal cross-sectional questionnaire survey, TREC survey		[67]
Physical health	Exhaustion	Beta: -0.054, p<0.001	Mixed-effects regression modelling	Alberta context tool (ACT)	N=1194 Care aides, urban nursing homes	Canada	Longitudinal cross-sectional questionnaire survey, TREC survey		[67]
Mental health	Exhaustion	Beta: -0.056, p<0.001	Mixed-effects regression modelling	Alberta context tool (ACT)	N=1194 Care aides, urban nursing homes	Canada	Longitudinal cross-sectional questionnaire survey, TREC survey		[67]
Age	cynicism	Beta: -0.098, p=0.008	Mixed-effects regression modelling	Alberta context tool (ACT)	N=1194 Care aides, urban nursing homes	Canada	Longitudinal cross-sectional questionnaire survey, TREC survey		[67]
English (native) as first language - No relative to Yes (ref)	cynicism	Beta: 0.281, p=0.002	Mixed-effects regression modelling	Alberta context tool (ACT)	N=1194 Care aides, urban nursing homes	Canada	Longitudinal cross-sectional questionnaire survey, TREC survey		[67]
Job satisfaction	cynicism	Beta: -0.330, p<0.001	Mixed-effects regression modelling	Alberta context tool (ACT)	N=1194 Care aides, urban nursing homes	Canada	Longitudinal cross-sectional questionnaire survey, TREC survey		[67]
responsive behaviours	cynicism	Beta: 0.127, p<0.001	Mixed-effects regression modelling	Alberta context tool (ACT)	N=1194 Care aides, urban nursing homes	Canada	Longitudinal cross-sectional questionnaire survey, TREC survey		[67]

physical health	cynicism	Beta: -0.014, p=0.007	Mixed-effects regression modelling	Alberta context tool (ACT)	N=1194 Care aides, urban nursing homes	Canada	Longitudinal cross-sectional questionnaire survey, TREC survey		[67]
mental health	cynicism	Beta: -0.049, p<0.001	Mixed-effects regression modelling	Alberta context tool (ACT)	N=1194 Care aides, urban nursing homes	Canada	Longitudinal cross-sectional questionnaire survey, TREC survey		[67]
Job satisfaction	efficacy	Beta: 0.151, p<0.001	Mixed-effects regression modelling	Alberta context tool (ACT)	N=1194 Care aides, urban nursing homes	Canada	Longitudinal cross-sectional questionnaire survey, TREC survey		[67]
Unit type - Secure dementia/Alzheimer's	job satisfaction	OR: 0.71, p=0.022	Multivariable analysis	Alberta context tool (ACT)	N=1224 care aides, long-term residential care	Canada	Longitudinal cross-sectional questionnaire survey, TREC survey		[68]
ACT concept: leadership	job satisfaction	OR: 1.30, p=0.021	Multivariable analysis	Alberta context tool (ACT)	N=1224 care aides, long-term residential care	Canada	Longitudinal cross-sectional questionnaire survey, TREC survey		[68]
ACT concept: culture	job satisfaction	OR: 2.86, p<0.001	Multivariable analysis	Alberta context tool (ACT)	N=1224 care aides, long-term residential care	Canada	Longitudinal cross-sectional questionnaire survey, TREC survey		[68]
ACT concept: social capital	job satisfaction	OR: 1.50, p=0.005	Multivariable analysis	Alberta context tool (ACT)	N=1224 care aides, long-term residential care	Canada	Longitudinal cross-sectional questionnaire survey, TREC survey		[68]
ACT concept: organisation	job satisfaction	OR: 1.23, p=0.002	Multivariable analysis	Alberta context tool (ACT)	N=1224 care aides, long-term residential care	Canada	Longitudinal cross-sectional questionnaire		[68]

al slack - staff							survey, TREC survey		
ACT concept: organisational slack - space	job satisfaction	OR: 1.14, p=0.05	Multivariable analysis	Alberta context tool (ACT)	N=1224 care aides, long-term residential care	Canada	Longitudinal cross-sectional questionnaire survey, TREC survey		[68]
ACT concept: organisational slack - time	job satisfaction	OR: 1.27, p=0.01	Multivariable analysis	Alberta context tool (ACT)	N=1224 care aides, long-term residential care	Canada	Longitudinal cross-sectional questionnaire survey, TREC survey		[68]
MBI scales: exhaustion	job satisfaction	OR: 0.85, p<0.001	Multivariable analysis	Alberta context tool (ACT)	N=1224 care aides, long-term residential care	Canada	Longitudinal cross-sectional questionnaire survey, TREC survey		[68]
MBI scales: cynicism	job satisfaction	OR: 0.87, p=0.005	Multivariable analysis	Alberta context tool (ACT)	N=1224 care aides, long-term residential care	Canada	Longitudinal cross-sectional questionnaire survey, TREC survey		[68]
MBI scales: professional efficacy	job satisfaction	OR: 1.33, p<0.001	Multivariable analysis	Alberta context tool (ACT)	N=1224 care aides, long-term residential care	Canada	Longitudinal cross-sectional questionnaire survey, TREC survey		[68]
English as first language (ref=English as additional language)	Conceptual use of best practices by care aides	p<0.001	significance testing	Alberta context tool (ACT)	N=1262 care aides, long-term residential care	Canada	Longitudinal cross-sectional questionnaire survey, TREC survey		[69]
MBI efficacy (job efficacy)	Conceptual use of best practices by care aides	p<0.001	significance testing	Alberta context tool (ACT)	N=1262 care aides, long-term residential care	Canada	Longitudinal cross-sectional questionnaire survey, TREC survey		[69]

Belief suspension - implement	Conceptual use of best practices by care aides	p<0.001	significanc e testing	Alberta context tool (ACT)	N=1262 care aides, long-term residential care	Canada	Longitudinal cross-sectional questionnaire survey, TREC survey		[69]
intent to use research	Conceptual use of best practices by care aides	p<0.001	significanc e testing	Alberta context tool (ACT)	N=1262 care aides, long-term residential care	Canada	Longitudinal cross-sectional questionnaire survey, TREC survey		[69]
information sources	Conceptual use of best practices by care aides	p<0.001	significanc e testing	Alberta context tool (ACT)	N=1262 care aides, long-term residential care	Canada	Longitudinal cross-sectional questionnaire survey, TREC survey		[69]
adequate knowledge	Conceptual use of best practices by care aides	p<0.001	significanc e testing	Alberta context tool (ACT)	N=1262 care aides, long-term residential care	Canada	Longitudinal cross-sectional questionnaire survey, TREC survey		[69]
ACT evaluation	Conceptual use of best practices by care aides	p<0.001	significanc e testing	Alberta context tool (ACT)	N=1262 care aides, long-term residential care	Canada	Longitudinal cross-sectional questionnaire survey, TREC survey		[69]
ACT organisation al slack - time	Conceptual use of best practices by care aides	p<0.001	significanc e testing	Alberta context tool (ACT)	N=1262 care aides, long-term residential care	Canada	Longitudinal cross-sectional questionnaire survey, TREC survey		[69]
ACT structural resources	Conceptual use of best practices by care aides	p<0.001	significanc e testing	Alberta context tool (ACT)	N=1262 care aides, long-term residential care	Canada	Longitudinal cross-sectional questionnaire survey, TREC survey		[69]
Province - Saskatchewa n	Conceptual use of best	p<0.001	significanc e testing	Alberta context tool (ACT)	N=1262 care aides, long-term residential care	Canada	Longitudinal cross-sectional questionnaire		[69]

	practices by care aides						survey, TREC survey		
Age	Instrumental use of best practices by care aides	p<0.001	significance testing	Alberta context tool (ACT)	N=1262 care aides, long-term residential care	Canada	Longitudinal cross-sectional questionnaire survey, TREC survey		[69]
Sex (ref=female)	Instrumental use of best practices by care aides	p=0.013	significance testing	Alberta context tool (ACT)	N=1262 care aides, long-term residential care	Canada	Longitudinal cross-sectional questionnaire survey, TREC survey		[69]
Shift worked - day (ref=night)	Instrumental use of best practices by care aides	p<0.001	significance testing	Alberta context tool (ACT)	N=1262 care aides, long-term residential care	Canada	Longitudinal cross-sectional questionnaire survey, TREC survey		[69]
MBI efficacy (job efficacy)	Instrumental use of best practices by care aides	p<0.001	significance testing	Alberta context tool (ACT)	N=1262 care aides, long-term residential care	Canada	Longitudinal cross-sectional questionnaire survey, TREC survey		[69]
Belief suspension - implement	Instrumental use of best practices by care aides	p<0.001	significance testing	Alberta context tool (ACT)	N=1262 care aides, long-term residential care	Canada	Longitudinal cross-sectional questionnaire survey, TREC survey		[69]
ACT social capital	Instrumental use of best practices by care aides	p<0.001	significance testing	Alberta context tool (ACT)	N=1262 care aides, long-term residential care	Canada	Longitudinal cross-sectional questionnaire survey, TREC survey		[69]
ACT organisational slack - staff	Instrumental use of best practices by care aides	p<0.001	significance testing	Alberta context tool (ACT)	N=1262 care aides, long-term residential care	Canada	Longitudinal cross-sectional questionnaire survey, TREC survey		[69]

ACT organisational slack - time	Instrumental use of best practices by care aides	p<0.001	significance testing	Alberta context tool (ACT)	N=1262 care aides, long-term residential care	Canada	Longitudinal cross-sectional questionnaire survey, TREC survey		[69]
ACT informal interaction	Instrumental use of best practices by care aides	p<0.001	significance testing	Alberta context tool (ACT)	N=1262 care aides, long-term residential care	Canada	Longitudinal cross-sectional questionnaire survey, TREC survey		[69]
Unit type (ref=general long-term care)	Instrumental use of best practices by care aides	p<0.001	significance testing	Alberta context tool (ACT)	N=1262 care aides, long-term residential care	Canada	Longitudinal cross-sectional questionnaire survey, TREC survey		[69]
Operation model - private for profit	Instrumental use of best practices by care aides	p<0.001	significance testing	Alberta context tool (ACT)	N=1262 care aides, long-term residential care	Canada	Longitudinal cross-sectional questionnaire survey, TREC survey		[69]
Province - Saskatchewan	Instrumental use of best practices by care aides	p<0.001	significance testing	Alberta context tool (ACT)	N=1262 care aides, long-term residential care	Canada	Longitudinal cross-sectional questionnaire survey, TREC survey		[69]
Burnout: cynicism	job satisfaction	Beta: -0.113, p=0.001	generalised estimating equations	Alberta context tool (ACT)	N=334 allied healthcare providers, residential long-term care	Canada	Longitudinal cross-sectional questionnaire survey, TREC survey		[70]
Empowerment: competence	job satisfaction	Beta: -0.224, p<0.001	generalised estimating equations	Alberta context tool (ACT)	N=334 allied healthcare providers, residential long-term care	Canada	Longitudinal cross-sectional questionnaire survey, TREC survey		[70]
Empowerment: meaning	job satisfaction	Beta: 0.232, p=0.001	generalised estimating equations	Alberta context tool (ACT)	N=334 allied healthcare providers,	Canada	Longitudinal cross-sectional questionnaire		[70]

					residential long-term care		survey, TREC survey		
Empowerment: self-determination	job satisfaction	Beta: 0.128, p=0.005	generalised estimating equations	Alberta context tool (ACT)	N=334 allied healthcare providers, residential long-term care	Canada	Longitudinal cross-sectional questionnaire survey, TREC survey		[70]
Empowerment: Impact	job satisfaction	Beta: 0.10, p=0.014	generalised estimating equations	Alberta context tool (ACT)	N=334 allied healthcare providers, residential long-term care	Canada	Longitudinal cross-sectional questionnaire survey, TREC survey		[70]
Social capital	job satisfaction	Beta: 0.158, p=0.012,	generalised estimating equations	Alberta context tool (ACT)	N=334 allied healthcare providers, residential long-term care	Canada	Longitudinal cross-sectional questionnaire survey, TREC survey		[70]
Organisational slack-time	job satisfaction	Beta: 0.096, p=0.029	generalised estimating equations	Alberta context tool (ACT)	N=334 allied healthcare providers, residential long-term care	Canada	Longitudinal cross-sectional questionnaire survey, TREC survey		[70]
formal interactions	job satisfaction	Beta: -0.057, p=0.033	generalised estimating equations	Alberta context tool (ACT)	N=334 allied healthcare providers, residential long-term care	Canada	Longitudinal cross-sectional questionnaire survey, TREC survey		[70]
adequate orientation	job satisfaction	Beta: 0.088, p=0.005	generalised estimating equations	Alberta context tool (ACT)	N=334 allied healthcare providers, residential long-term care	Canada	Longitudinal cross-sectional questionnaire survey, TREC survey		[70]
Suitability	Care-recipient satisfaction of quality	Rank: 0.215, P<0.001	Centrality	MAUT scaling procedure	N=76 homecare users	Canada	MAUT		[56]
Personal relation	Care-recipient	Rank: 0.200, P<0.001	Centrality	MAUT scaling procedure	N=76 homecare users	Canada	MAUT		[56]

	satisfaction of quality								
Continuity	Care-recipient satisfaction of quality	Rank: 0.198, P<0.001	Centrality	MAUT scaling procedure	N=76 homecare users	Canada	MAUT		[56]
Influence	Care-recipient satisfaction of quality	Rank: 0.196,P<0.001	Centrality	MAUT scaling procedure	N=76 homecare users	Canada	MAUT		[56]
Time/availability	Care-recipient satisfaction of quality	Rank: 0.191, P<0.001	Centrality	MAUT scaling procedure	N=76 homecare users	Canada	MAUT		[56]
staff continuity	continuity	n/a	n/a	The Multiattribute Utility Technology Diagram of Quality Attributes of Home Care	Homecare	n/a	Theoretical framework		[56]
care continuity	continuity	n/a	n/a	The Multiattribute Utility Technology Diagram of Quality Attributes of Home Care	Homecare	n/a	Theoretical framework		[56]
Respectful and considerate	Personal qualities	n/a	n/a	The Multiattribute Utility Technology Diagram of Quality Attributes of Home Care	Homecare	n/a	Theoretical framework		[56]
Quiet and reliable	Personal qualities	n/a	n/a	The Multiattribute Utility Technology Diagram of Quality Attributes of Home Care	Homecare	n/a	Theoretical framework		[56]
Careful and orderly	Personal qualities	n/a	n/a	The Multiattribute Utility Technology Diagram of Quality	Homecare	n/a	Theoretical framework		[56]

				Attributes of Home Care					
Good at housework	Professional competence	n/a	n/a	The Multiattribute Utility Technology Diagram of Quality Attributes of Home Care	Homecare	n/a	Theoretical framework		[56]
Good at giving personal care	Professional competence	n/a	n/a	The Multiattribute Utility Technology Diagram of Quality Attributes of Home Care	Homecare	n/a	Theoretical framework		[56]
Good at giving social care	Professional competence	n/a	n/a	The Multiattribute Utility Technology Diagram of Quality Attributes of Home Care	Homecare	n/a	Theoretical framework		[56]
Personal qualities	Suitability	n/a	n/a	The Multiattribute Utility Technology Diagram of Quality Attributes of Home Care	Homecare	n/a	Theoretical framework		[56]
Professional competence	Suitability	n/a	n/a	The Multiattribute Utility Technology Diagram of Quality Attributes of Home Care	Homecare	n/a	Theoretical framework		[56]
When (receive help)	Time/availab ility	n/a	n/a	The Multiattribute Utility Technology Diagram of Quality Attributes of Home Care	Homecare	n/a	Theoretical framework		[56]
Times are kept	Time/availab ility	n/a	n/a	The Multiattribute Utility Technology Diagram of Quality Attributes of Home Care	Homecare	n/a	Theoretical framework		[56]

Plenty of time	Time/availability	n/a	n/a	The Multiattribute Utility Technology Diagram of Quality Attributes of Home Care	Homecare	n/a	Theoretical framework		[56]
Be friendly	Personal relation	n/a	n/a	The Multiattribute Utility Technology Diagram of Quality Attributes of Home Care	Homecare	n/a	Theoretical framework		[56]
Sympathetic	Personal relation	n/a	n/a	The Multiattribute Utility Technology Diagram of Quality Attributes of Home Care	Homecare	n/a	Theoretical framework		[56]
Trustworthy	Personal relation	n/a	n/a	The Multiattribute Utility Technology Diagram of Quality Attributes of Home Care	Homecare	n/a	Theoretical framework		[56]
Continuity	Care-recipient satisfaction of quality	n/a	n/a	The Multiattribute Utility Technology Diagram of Quality Attributes of Home Care	Homecare	n/a	Theoretical framework		[56]
Suitability	Care-recipient satisfaction of quality	n/a	n/a	The Multiattribute Utility Technology Diagram of Quality Attributes of Home Care	Homecare	n/a	Theoretical framework		[56]
Time/availability	Care-recipient satisfaction of quality	n/a	n/a	The Multiattribute Utility Technology Diagram of Quality Attributes of Home Care	Homecare	n/a	Theoretical framework		[56]
Influence	Care-recipient	n/a	n/a	The Multiattribute Utility Technology Diagram of Quality	Homecare	n/a	Theoretical framework		[56]

	satisfaction of quality			Attributes of Home Care					
Personal relation	Care-recipient satisfaction of quality	n/a	n/a	The Multiattribute Utility Technology Diagram of Quality Attributes of Home Care	Homecare	n/a	Theoretical framework		[56]
Treatment-related needs	Patient needs	n/a	Literature review	literature review	Homecare	n/a	Literature review; theoretical framework		[63]
Activities of daily living (ADL)	Patient needs	n/a	Literature review	literature review	Homecare	n/a	Literature review; theoretical framework		[63]
Self-sufficiency needs	Patient needs	n/a	Literature review	literature review	Homecare	n/a	Literature review; theoretical framework		[63]
Social needs	Patient needs	n/a	Literature review	literature review	Homecare	n/a	Literature review; theoretical framework		[63]
Emotional needs	Patient needs	n/a	Literature review	literature review	Homecare	n/a	Literature review; theoretical framework		[63]
Informational needs	Patient needs	n/a	Literature review	literature review	Homecare	n/a	Literature review; theoretical framework		[63]
Living in urban area	More likely to live alone and have less access to informal caregiving	n/a	Literature review	literature review	Homecare	n/a	Literature review; theoretical framework		[63]
Living in rural area	Rely on informal care due to lack of access to formal services	n/a	Literature review	literature review	Homecare	n/a	Literature review; theoretical framework		[63]
Municipality	Amount and type of care received	n/a	Literature review	literature review	Homecare	n/a	Literature review; theoretical framework		[63]

Lack of recognition of self-harm ideation and untreated depression	Increased morbidity, mortality, institutionalisation, increased caregiver distress	n/a	Literature review	literature review	Homecare	n/a	Literature review; theoretical framework	[63]
Patients with higher depedency (lower functional ability), e.g., cancer	higher risk of malnutrition	n/a	Literature review	literature review	Homecare	n/a	Literature review; theoretical framework	[63]
Anorexia	higher risk of mortality	n/a	Literature review	literature review	Homecare	n/a	Literature review; theoretical framework	[63]
Close supervision by primary caregiver	reducing pressure ulcers	n/a	Literature review	literature review	Homecare	n/a	Literature review; theoretical framework	[63]
Education	reducing pressure ulcers	n/a	Literature review	literature review	Homecare	n/a	Literature review; theoretical framework	[63]
Polypharmacy	medication-related problems	n/a	Literature review	literature review	Homecare	n/a	Literature review; theoretical framework	[63]
Medication not suitable for dispensing system	medication-related problems	n/a	Literature review	literature review	Homecare	n/a	Literature review; theoretical framework	[63]
carer being interrupted when preparing medications at home	medication-related problems	n/a	Literature review	literature review	Homecare	n/a	Literature review; theoretical framework	[63]

changes in medication not being communicated	medication-related problems	n/a	Literature review	literature review	Homecare	n/a	Literature review; theoretical framework	[63]
suboptimal therapy	medication-related problems	n/a	Literature review	literature review	Homecare	n/a	Literature review; theoretical framework	[63]
use of unnecessary drugs	medication-related problems	n/a	Literature review	literature review	Homecare	n/a	Literature review; theoretical framework	[63]
medication-related problems	adverse events	n/a	Literature review	literature review	Homecare	n/a	Literature review; theoretical framework	[63]
falls	adverse events	n/a	Literature review	literature review	Homecare	n/a	Literature review; theoretical framework	[63]
malnourishment	falls	n/a	Literature review	literature review	Homecare	n/a	Literature review; theoretical framework	[63]
specific disorders: cardivascular disease, hypertension, diabetes mellitus	falls	n/a	Literature review	literature review	Homecare	n/a	Literature review; theoretical framework	[63]
pain	falls	n/a	Literature review	literature review	Homecare	n/a	Literature review; theoretical framework	[63]
reduced vision	falls	n/a	Literature review	literature review	Homecare	n/a	Literature review; theoretical framework	[63]
reduced cognitive ability	falls	n/a	Literature review	literature review	Homecare	n/a	Literature review; theoretical framework	[63]
environmental hazards	falls	n/a	Literature review	literature review	Homecare	n/a	Literature review; theoretical framework	[63]
hazards in home environment	falls	n/a	Literature review	literature review	Homecare	n/a	Literature review; theoretical framework	[63]

oral health	nutrition	n/a	Literature review	literature review	Homecare	n/a	Literature review; theoretical framework	[63]
visual decline	reduced social contact	n/a	Literature review	literature review	Homecare	n/a	Literature review; theoretical framework	[63]
contact with caregiver	meet comfort needs, alleviating loneliness	n/a	Literature review	literature review	Homecare	n/a	Literature review; theoretical framework	[63]
feels safe at home, receive empathy, receive encouragem ent	participation in healthcare	n/a	Literature review	literature review	Homecare	n/a	Literature review; theoretical framework	[63]
medication regimens	informationa l need	n/a	Literature review	literature review	Homecare	n/a	Literature review; theoretical framework	[63]
disease or condition, and severity	informationa l need	n/a	Literature review	literature review	Homecare	n/a	Literature review; theoretical framework	[63]
nonmedical care	informationa l need	n/a	Literature review	literature review	Homecare	n/a	Literature review; theoretical framework	[63]
functional limitations	informationa l need	n/a	Literature review	literature review	Homecare	n/a	Literature review; theoretical framework	[63]
continious updated information during treatment, care and disease	informationa l need	n/a	Literature review	literature review	Homecare	n/a	Literature review; theoretical framework	[63]
homecare	increase acceptibility of treatment	n/a	Literature review	literature review	Homecare	n/a	Literature review; theoretical framework	[63]
social contact with	quality of life	n/a	Literature review	literature review	Homecare	n/a	Literature review; theoretical framework	[63]

lives and families								
patient-centred homecare (emotional, psychosocial aspects of disease, health-promoting activities, restorative homecare)	increase empowerment and independence	n/a	Literature review	literature review	Homecare	n/a	Literature review; theoretical framework	[63]
increased empowerment and independence	increases quality of life	n/a	Literature review	literature review	Homecare	n/a	Literature review; theoretical framework	[63]
emotional support	increases quality of life	n/a	Literature review	literature review	Homecare	n/a	Literature review; theoretical framework	[63]
depression, anxiety, pain	reduces quality of life	n/a	Literature review	literature review	Homecare	n/a	Literature review; theoretical framework	[63]
delivery of therapies at home	increases quality of life	n/a	Literature review	literature review	Homecare	n/a	Literature review; theoretical framework	[63]
assistive devices	increased patient satisfaction	n/a	Literature review	literature review	Homecare	n/a	Literature review; theoretical framework	[63]
availability	increased patient satisfaction	n/a	Literature review	literature review	Homecare	n/a	Literature review; theoretical framework	[63]
accessibility (nondiscriminatory, physical	increased patient satisfaction	n/a	Literature review	literature review	Homecare	n/a	Literature review; theoretical framework	[63]

accessibility, affordability, information accessibility)								
acceptability (e.g., adequate conditions at home, not being afraid to use assistive devices or follow treatments)	increased patient satisfaction	n/a	Literature review	literature review	Homecare	n/a	Literature review; theoretical framework	[63]
quality	increased patient satisfaction	n/a	Literature review	literature review	Homecare	n/a	Literature review; theoretical framework	[63]
homecare	reduces hospitalisati on, readmission rates, use of emergency services	n/a	Literature review	literature review	Homecare	n/a	Literature review; theoretical framework	[63]
Homecare	reduces exposure to nosocomial risks	n/a	Literature review	literature review	Homecare	n/a	Literature review; theoretical framework	[63]
homecare	higher attendance rates	n/a	Literature review	literature review	Homecare	n/a	Literature review; theoretical framework	[63]
homecare	alleviating risk of seeking care to late	n/a	Literature review	literature review	Homecare	n/a	Literature review; theoretical framework	[63]

reduces exposure to nosocomial risks	reduces risk of hospitalisation	n/a	Literature review	literature review	Homecare	n/a	Literature review; theoretical framework	[63]
higher attendance rates	reduces risk of hospitalisation	n/a	Literature review	literature review	Homecare	n/a	Literature review; theoretical framework	[63]
alleviating risk of seeking care to late	reduces risk of hospitalisation	n/a	Literature review	literature review	Homecare	n/a	Literature review; theoretical framework	[63]
burden of care	informal caregiver concern (The Family Difficulty Scale)	n/a	Literature review	literature review	Homecare	n/a	Literature review; theoretical framework	[63]
concerns about homecare doctor	informal caregiver concern (The Family Difficulty Scale)	n/a	Literature review	literature review	Homecare	n/a	Literature review; theoretical framework	[63]
balance of work and care	informal caregiver concern (The Family Difficulty Scale)	n/a	Literature review	literature review	Homecare	n/a	Literature review; theoretical framework	[63]
patient's pain and condition	informal caregiver concern (The Family Difficulty Scale)	n/a	Literature review	literature review	Homecare	n/a	Literature review; theoretical framework	[63]
concerns about	informal caregiver	n/a	Literature review	literature review	Homecare	n/a	Literature review; theoretical framework	[63]

visiting nurse	concern (The Family Difficulty Scale)							
concerns about homecare service	informal caregiver concern (The Family Difficulty Scale)	n/a	Literature review	literature review	Homecare	n/a	Literature review; theoretical framework	[63]
relationships between family caregivers and their families	informal caregiver concern (The Family Difficulty Scale)	n/a	Literature review	literature review	Homecare	n/a	Literature review; theoretical framework	[63]
funeral preparations	informal caregiver concern (The Family Difficulty Scale)	n/a	Literature review	literature review	Homecare	n/a	Literature review; theoretical framework	[63]
Severity of patient's condition	informal caregiver distress	n/a	Literature review	literature review	Homecare	n/a	Literature review; theoretical framework	[63]
Patient functionality	informal caregiver distress	n/a	Literature review	literature review	Homecare	n/a	Literature review; theoretical framework	[63]
degree of carer's positive outlook and personal resources	informal caregiver distress	n/a	Literature review	literature review	Homecare	n/a	Literature review; theoretical framework	[63]
Uncertainty with regard to outlook and	informal caregiver distress	n/a	Literature review	literature review	Homecare	n/a	Literature review; theoretical framework	[63]

continuity of financial burdens								
Provision of care	informal caregiver burden	n/a	Literature review	literature review	Homecare	n/a	Literature review; theoretical framework	[63]
prevention of injuries	informal caregiver burden	n/a	Literature review	literature review	Homecare	n/a	Literature review; theoretical framework	[63]
home safety	informal caregiver burden	n/a	Literature review	literature review	Homecare	n/a	Literature review; theoretical framework	[63]
Good caregiver-nurse relationship	assisting assessment of needs, improving access to care	n/a	Literature review	literature review	Homecare	n/a	Literature review; theoretical framework	[63]
Poor caregiver-nurse relationship	feelings of powerlessness	n/a	Literature review	literature review	Homecare	n/a	Literature review; theoretical framework	[63]
gendered standards	female informal care givers	n/a	Literature review	literature review	Homecare	n/a	Literature review; theoretical framework	[63]
Cocreation	prerequisite of intimate care	n/a	Literature review	literature review	Homecare	n/a	Literature review; theoretical framework	[63]
Cooperation of entire family	greater family health	n/a	Literature review	literature review	Homecare	n/a	Literature review; theoretical framework	[63]
presence of informal caregiver	reduces nonadherence	n/a	Literature review	literature review	Homecare	n/a	Literature review; theoretical framework	[63]
caregiver strain	increased non-adherence	n/a	Literature review	literature review	Homecare	n/a	Literature review; theoretical framework	[63]

caregiver confidence in patient's abilities	improvements in ADL	n/a	Literature review	literature review	Homecare	n/a	Literature review; theoretical framework	[63]
caregiver and care recipient confident about abilities	improvements in ADL	n/a	Literature review	literature review	Homecare	n/a	Literature review; theoretical framework	[63]
professional training	improvements in ADL	n/a	Literature review	literature review	Homecare	n/a	Literature review; theoretical framework	[63]
partnerships with pharmacists	improvements in ADL	n/a	Literature review	literature review	Homecare	n/a	Literature review; theoretical framework	[63]
community activities	improve communication and collaboration	n/a	Literature review	literature review	Homecare	n/a	Literature review; theoretical framework	[63]
ICT tools	increase accessibility	n/a	Literature review	literature review	Homecare	n/a	Literature review; theoretical framework	[63]
ICT tools	patient gain control and promote self-management	n/a	Literature review	literature review	Homecare	n/a	Literature review; theoretical framework	[63]
educational resources	technological implementation	n/a	Literature review	literature review	Homecare	n/a	Literature review; theoretical framework	[63]
user-centred design	technological implementation	n/a	Literature review	literature review	Homecare	n/a	Literature review; theoretical framework	[63]
simulation technology	aid training	n/a	Literature review	literature review	Homecare	n/a	Literature review; theoretical framework	[63]

spouse	informal caregiving	p<0.001	t-test	WHOQoL-Age questionnaire; Abbreviated Mental Test Score; Depression Scale Short Form	Homecare, N=138 informal caregivers	Poland	Cross-sectional study		[101]
children	informal caregiving	p<0.001	t-test	WHOQoL-Age questionnaire; Abbreviated Mental Test Score; Depression Scale Short Form	Homecare, N=138 informal caregivers	Poland	Cross-sectional study		[101]
support provided by others	QoL of informal caregiver	Beta: 0.605 (P=0.000)	multivariate analysis.	WHOQoL-Age questionnaire; Abbreviated Mental Test Score; Depression Scale Short Form	Homecare, N=138 informal caregivers	Poland	Cross-sectional study		[101]
Experience in care	QoL of informal caregiver	Beta: -0.220 (P=0.002)	multivariate analysis.	WHOQoL-Age questionnaire; Abbreviated Mental Test Score; Depression Scale Short Form	Homecare, N=138 informal caregivers	Poland	Cross-sectional study		[101]
IC's health self-assessment	QoL of informal caregiver	Beta: 0.174 (P=0.012)	multivariate analysis.	WHOQoL-Age questionnaire; Abbreviated Mental Test Score; Depression Scale Short Form	Homecare, N=138 informal caregivers	Poland	Cross-sectional study		[101]
Depressive symptoms in care-receiver	QoL of informal caregiver	Beta: 0.178 (P=0.010)	multivariate analysis.	WHOQoL-Age questionnaire; Abbreviated Mental Test Score; Depression Scale Short Form	Homecare, N=138 informal caregivers	Poland	Cross-sectional study		[101]

Polypharmacy	Hospitalisation	P<0.05	t-test	Medication assessment; Delphi consensus	833 discharges, homecare patients	USA	Retrospective chart review		[102]
Sociability	Homecare incidence	Marginal effect: 0.02, z-value: 2.25	multivariate modelling	Survey of Health, Ageing, and Retirement in Europe (SHARE)	Informal homecare, single person households, N=1259 single men, N=3528 women	Europe (Austria, Germany, Sweden, The Netherlands, Spain, Italy, France, Denmark, Belgium)	Survey based study		[71]
Men, age	Homecare incidence	Marginal effect: 0.01, z-value: 3.54	multivariate modelling	Survey of Health, Ageing, and Retirement in Europe (SHARE)	Informal homecare, single person households, N=1259 single men, N=3528 women	Europe (Austria, Germany, Sweden, The Netherlands, Spain, Italy, France, Denmark, Belgium)	Survey based study		[71]
Men, gali	Homecare incidence	Marginal effect: 0.11, z-value: 2.98	multivariate modelling	Survey of Health, Ageing, and Retirement in Europe (SHARE)	Informal homecare, single person households, N=1259 single men, N=3528 women	Europe (Austria, Germany, Sweden, The Netherlands,	Survey based study		[71]

						Spain, Italy, France, Denmark, Belgium)			
Men, IADL	Homecare incidence	Marginal effect: 0.31, z-value: 6.56	multivariate modelling	Survey of Health, Ageing, and Retirement in Europe (SHARE)	Informal homecare, single person households, N=1259 single men, N=3528 women	Europe (Austria, Germany, Sweden, The Netherlands, Spain, Italy, France, Denmark, Belgium)	Survey based study		[71]
Men, mobility	Homecare incidence	Marginal effect: 0.08, z-value: 1.98	multivariate modelling	Survey of Health, Ageing, and Retirement in Europe (SHARE)	Informal homecare, single person households, N=1259 single men, N=3528 women	Europe (Austria, Germany, Sweden, The Netherlands, Spain, Italy, France, Denmark, Belgium)	Survey based study		[71]
Men, mental health	Homecare incidence	Marginal effect: 0.08, z-value: 1.99	multivariate modelling	Survey of Health, Ageing, and Retirement in Europe (SHARE)	Informal homecare, single person households, N=1259 single	Europe (Austria, Germany, Sweden,	Survey based study		[71]

					men, N=3528 women	The Netherlands, Spain, Italy, France, Denmark, Belgium)			
Women, age	Homecare incidence	Marginal effect: 0.01, z-value: 5.49	multivariate modelling	Survey of Health, Ageing, and Retirement in Europe (SHARE)	Informal homecare, single person households, N=1259 single men, N=3528 women	Europe (Austria, Germany, Sweden, The Netherlands, Spain, Italy, France, Denmark, Belgium)	Survey based study		[71]
Women, ADL	Homecare incidence	Marginal effect: 0.13, z-value: 4.58	multivariate modelling	Survey of Health, Ageing, and Retirement in Europe (SHARE)	Informal homecare, single person households, N=1259 single men, N=3528 women	Europe (Austria, Germany, Sweden, The Netherlands, Spain, Italy, France, Denmark, Belgium)	Survey based study		[71]

Women, gali	Homecare incidence	Marginal effect: 0.12, z-value: 5.61	multivariate modelling	Survey of Health, Ageing, and Retirement in Europe (SHARE)	Informal homecare, single person households, N=1259 single men, N=3528 women	Europe (Austria, Germany, Sweden, The Netherlands, Spain, Italy, France, Denmark, Belgium)	Survey based study		[71]
Women, IADL	Homecare incidence	Marginal effect: 0.22, z-value: 9.42	multivariate modelling	Survey of Health, Ageing, and Retirement in Europe (SHARE)	Informal homecare, single person households, N=1259 single men, N=3528 women	Europe (Austria, Germany, Sweden, The Netherlands, Spain, Italy, France, Denmark, Belgium)	Survey based study		[71]
Women, severe condition	Homecare incidence	Marginal effect: 0.07, z-value: 3.41	multivariate modelling	Survey of Health, Ageing, and Retirement in Europe (SHARE)	Informal homecare, single person households, N=1259 single men, N=3528 women	Europe (Austria, Germany, Sweden, The Netherlands, Spain, Italy, France,	Survey based study		[71]

						Denmark, Belgium)			
Women, mobility	Homecare incidence	Marginal effect: 0.13, z-value: 5.59	multivariate modelling	Survey of Health, Ageing, and Retirement in Europe (SHARE)	Informal homecare, single person households, N=1259 single men, N=3528 women	Europe (Austria, Germany, Sweden, The Netherlands, Spain, Italy, France, Denmark, Belgium)	Survey based study		[71]
Women, reading	Homecare incidence	Marginal effect: 0.08, z-value: 2.53	multivariate modelling	Survey of Health, Ageing, and Retirement in Europe (SHARE)	Informal homecare, single person households, N=1259 single men, N=3528 women	Europe (Austria, Germany, Sweden, The Netherlands, Spain, Italy, France, Denmark, Belgium)	Survey based study		[71]
Single male	Hours of homecare children	Marginal effect: -47.05, z-value: -3.46	multivariate modelling	Survey of Health, Ageing, and Retirement in Europe (SHARE)	Informal homecare, single person households, N=1259 single men, N=3528 women	Europe (Austria, Germany, Sweden, The Netherlands,	Survey based study		[71]

						Spain, Italy, France, Denmark, Belgium)			
number of grandchildren	Hours of homecare children	Marginal effect: -0.68, z-value: -3.89	multivariate modelling	Survey of Health, Ageing, and Retirement in Europe (SHARE)	Informal homecare, single person households, N=1259 single men, N=3528 women	Europe (Austria, Germany, Sweden, The Netherlands, Spain, Italy, France, Denmark, Belgium)	Survey based study		[71]
number of sons	Hours of homecare children	Marginal effect: 4.63, z-value: 4.78	multivariate modelling	Survey of Health, Ageing, and Retirement in Europe (SHARE)	Informal homecare, single person households, N=1259 single men, N=3528 women	Europe (Austria, Germany, Sweden, The Netherlands, Spain, Italy, France, Denmark, Belgium)	Survey based study		[71]
distance of sons (average)	Hours of homecare children	Marginal effect: -12.37, z-value: -4.68	multivariate modelling	Survey of Health, Ageing, and Retirement in Europe (SHARE)	Informal homecare, single person households, N=1259 single	Europe (Austria, Germany, Sweden,	Survey based study		[71]

					men, N=3528 women	The Netherlands, Spain, Italy, France, Denmark, Belgium)			
Has daughters	Hours of homecare children	Marginal effect: 22.52, z-value: 3.17	multivariate modelling	Survey of Health, Ageing, and Retirement in Europe (SHARE)	Informal homecare, single person households, N=1259 single men, N=3528 women	Europe (Austria, Germany, Sweden, The Netherlands, Spain, Italy, France, Denmark, Belgium)	Survey based study		[71]
Distance of daugthers	Hours of homecare children	Marginal effect: -14.89, z-value: 4.95	multivariate modelling	Survey of Health, Ageing, and Retirement in Europe (SHARE)	Informal homecare, single person households, N=1259 single men, N=3528 women	Europe (Austria, Germany, Sweden, The Netherlands, Spain, Italy, France, Denmark, Belgium)	Survey based study		[71]

Employment daughters	Hours of homecare children	Marginal effect: -3.2, z-value: -2.01	multivariate modelling	Survey of Health, Ageing, and Retirement in Europe (SHARE)	Informal homecare, single person households, N=1259 single men, N=3528 women	Europe (Austria, Germany, Sweden, The Netherlands, Spain, Italy, France, Denmark, Belgium)	Survey based study		[71]
Men, age	Hours of homecare children	Marginal effect: 0.67, z-value: 3.85	multivariate modelling	Survey of Health, Ageing, and Retirement in Europe (SHARE)	Informal homecare, single person households, N=1259 single men, N=3528 women	Europe (Austria, Germany, Sweden, The Netherlands, Spain, Italy, France, Denmark, Belgium)	Survey based study		[71]
Men, years of education	Hours of homecare children	Marginal effect: -0.54, z-value: -2.2	multivariate modelling	Survey of Health, Ageing, and Retirement in Europe (SHARE)	Informal homecare, single person households, N=1259 single men, N=3528 women	Europe (Austria, Germany, Sweden, The Netherlands, Spain, Italy, France,	Survey based study		[71]

						Denmark, Belgium)			
Men, reading	Hours of homecare children	Marginal effect: 6.77, z-value: 2.04	multivariate modelling	Survey of Health, Ageing, and Retirement in Europe (SHARE)	Informal homecare, single person households, N=1259 single men, N=3528 women	Europe (Austria, Germany, Sweden, The Netherlands, Spain, Italy, France, Denmark, Belgium)	Survey based study		[71]
Women, years of education	Hours of homecare children	Marginal effect: -0.46, z-value: -3.3	multivariate modelling	Survey of Health, Ageing, and Retirement in Europe (SHARE)	Informal homecare, single person households, N=1259 single men, N=3528 women	Europe (Austria, Germany, Sweden, The Netherlands, Spain, Italy, France, Denmark, Belgium)	Survey based study		[71]
Women, IADL	Hours of homecare children	Marginal effect: 2.79, z-value: 2.29	multivariate modelling	Survey of Health, Ageing, and Retirement in Europe (SHARE)	Informal homecare, single person households, N=1259 single men, N=3528 women	Europe (Austria, Germany, Sweden, The Netherlands,	Survey based study		[71]

						Spain, Italy, France, Denmark, Belgium)			
Women, severe condition	Hours of homecare children	Marginal effect: -3.18, z-value: -2.7	multivariate modelling	Survey of Health, Ageing, and Retirement in Europe (SHARE)	Informal homecare, single person households, N=1259 single men, N=3528 women	Europe (Austria, Germany, Sweden, The Netherlands, Spain, Italy, France, Denmark, Belgium)	Survey based study		[71]
Children	Relatives	Marginal effect: -0.32, z-value: -4.84	multivariate modelling	Survey of Health, Ageing, and Retirement in Europe (SHARE)	Informal homecare, single person households, N=1259 single men, N=3528 women	Europe (Austria, Germany, Sweden, The Netherlands, Spain, Italy, France, Denmark, Belgium)	Survey based study		[71]
Chidren	Friends	Marginal effect: -0.17, z-value: -2.52	multivariate modelling	Survey of Health, Ageing, and Retirement in Europe (SHARE)	Informal homecare, single person households, N=1259 single	Europe (Austria, Germany, Sweden,	Survey based study		[71]

					men, N=3528 women	The Netherlands, Spain, Italy, France, Denmark, Belgium)			
Children	Formal care	Marginal effect: -0.17, z-value: -4.19	multivariate modelling	Survey of Health, Ageing, and Retirement in Europe (SHARE)	Informal homecare, single person households, N=1259 single men, N=3528 women	Europe (Austria, Germany, Sweden, The Netherlands, Spain, Italy, France, Denmark, Belgium)	Survey based study		[71]
Relatives	Friends	Marginal effect: -0.17, z-value: -2.22	multivariate modelling	Survey of Health, Ageing, and Retirement in Europe (SHARE)	Informal homecare, single person households, N=1259 single men, N=3528 women	Europe (Austria, Germany, Sweden, The Netherlands, Spain, Italy, France, Denmark, Belgium)	Survey based study		[71]

Relatives	Formal care	Marginal effect: -0.11, z-value: -2.1	multivariate modelling	Survey of Health, Ageing, and Retirement in Europe (SHARE)	Informal homecare, single person households, N=1259 single men, N=3528 women	Europe (Austria, Germany, Sweden, The Netherlands, Spain, Italy, France, Denmark, Belgium)	Survey based study		[71]
Friends	Formal care	Marginal effect: -0.2, z-value: -4.71	multivariate modelling	Survey of Health, Ageing, and Retirement in Europe (SHARE)	Informal homecare, single person households, N=1259 single men, N=3528 women	Europe (Austria, Germany, Sweden, The Netherlands, Spain, Italy, France, Denmark, Belgium)	Survey based study		[71]
number of grandchildren	Hours of homecare relatives	Marginal effect: 0.36, z-value: 2.02	multivariate modelling	Survey of Health, Ageing, and Retirement in Europe (SHARE)	Informal homecare, single person households, N=1259 single men, N=3528 women	Europe (Austria, Germany, Sweden, The Netherlands, Spain, Italy, France,	Survey based study		[71]

						Denmark, Belgium)			
Number of siblings	Hours of homecare relatives	Marginal effect: 0.68, z-value: 2.91	multivariate modelling	Survey of Health, Ageing, and Retirement in Europe (SHARE)	Informal homecare, single person households, N=1259 single men, N=3528 women	Europe (Austria, Germany, Sweden, The Netherlands, Spain, Italy, France, Denmark, Belgium)	Survey based study		[71]
Number of sons	Hours of homecare relatives	Marginal effect: -3.27, z-value: -2.69	multivariate modelling	Survey of Health, Ageing, and Retirement in Europe (SHARE)	Informal homecare, single person households, N=1259 single men, N=3528 women	Europe (Austria, Germany, Sweden, The Netherlands, Spain, Italy, France, Denmark, Belgium)	Survey based study		[71]
Number of daugthers	Hours of homecare relatives	Marginal effect: -2.97, z-value: -2.67	multivariate modelling	Survey of Health, Ageing, and Retirement in Europe (SHARE)	Informal homecare, single person households, N=1259 single men, N=3528 women	Europe (Austria, Germany, Sweden, The Netherlands,	Survey based study		[71]

						Spain, Italy, France, Denmark , Belgium)			
Distance of daugthers (average)	Hours of homecare relatives	Marginal effect: 7.88, z-value: 2.13	multivariat e modelling	Survey of Health, Ageing, and Retirement in Europe (SHARE)	Informal homecare, single person households, N=1259 single men, N=3528 women	Europe (Austria, Germany , Sweden, The Netherla nds, Spain, Italy, France, Denmark , Belgium)	Survey based study		[71]
Number of sons	Hours of homecare friends	Marginal effect: -2.00, z-value: - 2.77	multivariat e modelling	Survey of Health, Ageing, and Retirement in Europe (SHARE)	Informal homecare, single person households, N=1259 single men, N=3528 women	Europe (Austria, Germany , Sweden, The Netherla nds, Spain, Italy, France, Denmark , Belgium)	Survey based study		[71]
Distance sons (average)	Hours of homecare friends	Marginal effect: 6.14, z-value: 2.70	multivariat e modelling	Survey of Health, Ageing, and Retirement in Europe (SHARE)	Informal homecare, single person households, N=1259 single	Europe (Austria, Germany , Sweden,	Survey based study		[71]

					men, N=3528 women	The Netherlands, Spain, Italy, France, Denmark, Belgium)			
Number of daughters	Hours of homecare friends	Marginal effect: -2.07, z-value: -2.67	multivariate modelling	Survey of Health, Ageing, and Retirement in Europe (SHARE)	Informal homecare, single person households, N=1259 single men, N=3528 women	Europe (Austria, Germany, Sweden, The Netherlands, Spain, Italy, France, Denmark, Belgium)	Survey based study		[71]
Distance of daughters (average)	Hours of homecare friends	Marginal effect: 11.66, z-value: 4.41	multivariate modelling	Survey of Health, Ageing, and Retirement in Europe (SHARE)	Informal homecare, single person households, N=1259 single men, N=3528 women	Europe (Austria, Germany, Sweden, The Netherlands, Spain, Italy, France, Denmark, Belgium)	Survey based study		[71]

Women, age	Hours of homecare friends	Marginal effect: -0.17, z-value: -2.3	multivariate modelling	Survey of Health, Ageing, and Retirement in Europe (SHARE)	Informal homecare, single person households, N=1259 single men, N=3528 women	Europe (Austria, Germany, Sweden, The Netherlands, Spain, Italy, France, Denmark, Belgium)	Survey based study		[71]
Men, age	Hours of formal homecare	Marginal effect: 0.21, z-value: 3.09	multivariate modelling	Survey of Health, Ageing, and Retirement in Europe (SHARE)	Informal homecare, single person households, N=1259 single men, N=3528 women	Europe (Austria, Germany, Sweden, The Netherlands, Spain, Italy, France, Denmark, Belgium)	Survey based study		[71]
Men, IADL	Hours of formal homecare	Marginal effect: 3.77, z-value: 3.44	multivariate modelling	Survey of Health, Ageing, and Retirement in Europe (SHARE)	Informal homecare, single person households, N=1259 single men, N=3528 women	Europe (Austria, Germany, Sweden, The Netherlands, Spain, Italy, France,	Survey based study		[71]

						Denmark<br>,<br>Belgium)			
Women, age	Hours of formal homecare	Marginal effect: 0.26, z-value: 5.2	multivariat e modelling	Survey of Health, Ageing, and Retirement in Europe (SHARE)	Informal homecare, single person households, N=1259 single men, N=3528 women	Europe (Austria, Germany<br>,<br>Sweden, The Netherla nds, Spain, Italy, France, Denmark<br>,<br>Belgium)	Survey based study		[71]
Women, years of education	Hours of formal homecare	Marginal effect: 0.21, z-value: 2.57	multivariat e modelling	Survey of Health, Ageing, and Retirement in Europe (SHARE)	Informal homecare, single person households, N=1259 single men, N=3528 women	Europe (Austria, Germany<br>,<br>Sweden, The Netherla nds, Spain, Italy, France, Denmark<br>,<br>Belgium)	Survey based study		[71]
Women, ADL	Hours of formal homecare	Marginal effect: 3.74, z-value: 5.89	multivariat e modelling	Survey of Health, Ageing, and Retirement in Europe (SHARE)	Informal homecare, single person households, N=1259 single men, N=3528 women	Europe (Austria, Germany<br>,<br>Sweden, The Netherla nds,	Survey based study		[71]

						Spain, Italy, France, Denmark, Belgium)			
Women, gali	Hours of formal homecare	Marginal effect: 1.78, z-value: 2.90	multivariate modelling	Survey of Health, Ageing, and Retirement in Europe (SHARE)	Informal homecare, single person households, N=1259 single men, N=3528 women	Europe (Austria, Germany, Sweden, The Netherlands, Spain, Italy, France, Denmark, Belgium)	Survey based study		[71]
Women, IADL	Hours of formal homecare	Marginal effect: 3.83, z-value: 6.96	multivariate modelling	Survey of Health, Ageing, and Retirement in Europe (SHARE)	Informal homecare, single person households, N=1259 single men, N=3528 women	Europe (Austria, Germany, Sweden, The Netherlands, Spain, Italy, France, Denmark, Belgium)	Survey based study		[71]
Women, severe condition	Hours of formal homecare	Marginal effect: 1.66, z-value: 2.94	multivariate modelling	Survey of Health, Ageing, and Retirement in Europe (SHARE)	Informal homecare, single person households, N=1259 single	Europe (Austria, Germany, Sweden,	Survey based study		[71]

					men, N=3528 women	The Netherlands, Spain, Italy, France, Denmark, Belgium)			
Women, numeracy	Hours of formal homecare	Marginal effect: 1.27, z-value: 2.11	multivariate modelling	Survey of Health, Ageing, and Retirement in Europe (SHARE)	Informal homecare, single person households, N=1259 single men, N=3528 women	Europe (Austria, Germany, Sweden, The Netherlands, Spain, Italy, France, Denmark, Belgium)	Survey based study		[71]
Number of sons	Homecare incidence	Marginal effect: 0.03, z-value: 2.8	multivariate modelling	Survey of Health, Ageing, and Retirement in Europe (SHARE)	Informal homecare, couples households N=5557	Europe (Austria, Germany, Sweden, The Netherlands, Spain, Italy, France, Denmark, Belgium)	Survey based study		[71]

Employment sons (average)	Homecare incidence	Marginal effect: -0.08, z-value: -3.16	multivariate modelling	Survey of Health, Ageing, and Retirement in Europe (SHARE)	Informal homecare, couples households N=5557	Europe (Austria, Germany, Sweden, The Netherlands, Spain, Italy, France, Denmark, Belgium)	Survey based study		[71]
Men, ADL	Homecare incidence	Marginal effect: 0.05, z-value: 2.23	multivariate modelling	Survey of Health, Ageing, and Retirement in Europe (SHARE)	Informal homecare, couples households N=5557	Europe (Austria, Germany, Sweden, The Netherlands, Spain, Italy, France, Denmark, Belgium)	Survey based study		[71]
Men, Gali	Homecare incidence	Marginal effect: 0.05, z-value: 3.52	multivariate modelling	Survey of Health, Ageing, and Retirement in Europe (SHARE)	Informal homecare, couples households N=5557	Europe (Austria, Germany, Sweden, The Netherlands, Spain, Italy, France,	Survey based study		[71]

						Denmark, Belgium)			
Men, IADL	Homecare incidence	Marginal effect: 0.16, z-value: 7.89	multivariate modelling	Survey of Health, Ageing, and Retirement in Europe (SHARE)	Informal homecare, couples households N=5557	Europe (Austria, Germany, Sweden, The Netherlands, Spain, Italy, France, Denmark, Belgium)	Survey based study		[71]
Men, mobility	Homecare incidence	Marginal effect: 0.07, z-value: 4.92	multivariate modelling	Survey of Health, Ageing, and Retirement in Europe (SHARE)	Informal homecare, couples households N=5557	Europe (Austria, Germany, Sweden, The Netherlands, Spain, Italy, France, Denmark, Belgium)	Survey based study		[71]
Women, ADL	Homecare incidence	Marginal effect: 0.05, z-value: 2.54	multivariate modelling	Survey of Health, Ageing, and Retirement in Europe (SHARE)	Informal homecare, couples households N=5557	Europe (Austria, Germany, Sweden, The Netherlands,	Survey based study		[71]

						Spain, Italy, France, Denmark, Belgium)			
Women, Gali	Homecare incidence	Marginal effect: 0.06, z-value: 4.63	multivariate modelling	Survey of Health, Ageing, and Retirement in Europe (SHARE)	Informal homecare, couples households N=5557	Europe (Austria, Germany, Sweden, The Netherlands, Spain, Italy, France, Denmark, Belgium)	Survey based study		[71]
Women, IADL	Homecare incidence	Marginal effect: 0.1, z-value: 6.20	multivariate modelling	Survey of Health, Ageing, and Retirement in Europe (SHARE)	Informal homecare, couples households N=5557	Europe (Austria, Germany, Sweden, The Netherlands, Spain, Italy, France, Denmark, Belgium)	Survey based study		[71]
Women, severe condition	Homecare incidence	Marginal effect: 0.05, z-value: 3.31	multivariate modelling	Survey of Health, Ageing, and Retirement in Europe (SHARE)	Informal homecare, couples households N=5557	Europe (Austria, Germany, Sweden,	Survey based study		[71]

						The Netherlands, Spain, Italy, France, Denmark, Belgium)			
Women, numeracy	Homecare incidence	Marginal effect: 0.03, z-value: 2.07	multivariate modelling	Survey of Health, Ageing, and Retirement in Europe (SHARE)	Informal homecare, couples households N=5557	Europe (Austria, Germany, Sweden, The Netherlands, Spain, Italy, France, Denmark, Belgium)	Survey based study		[71]
Number of sons	Hours of homecare children	Marginal effect: 3.04, z-value: 2.36	multivariate modelling	Survey of Health, Ageing, and Retirement in Europe (SHARE)	Informal homecare, couples households N=5557	Europe (Austria, Germany, Sweden, The Netherlands, Spain, Italy, France, Denmark, Belgium)	Survey based study		[71]

Distance sons (average)	Hours of homecare children	Marginal effect: -10.34, z-value: -2.55	multivariate modelling	Survey of Health, Ageing, and Retirement in Europe (SHARE)	Informal homecare, couples households N=5557	Europe (Austria, Germany, Sweden, The Netherlands, Spain, Italy, France, Denmark, Belgium)	Survey based study		[71]
Has daughters	Hours of homecare children	Marginal effect: 31.25, z-value: 3.13	multivariate modelling	Survey of Health, Ageing, and Retirement in Europe (SHARE)	Informal homecare, couples households N=5557	Europe (Austria, Germany, Sweden, The Netherlands, Spain, Italy, France, Denmark, Belgium)	Survey based study		[71]
Number of daugthers	Hours of homecare children	Marginal effect: 2.82, z-value: 2.2	multivariate modelling	Survey of Health, Ageing, and Retirement in Europe (SHARE)	Informal homecare, couples households N=5557	Europe (Austria, Germany, Sweden, The Netherlands, Spain, Italy, France,	Survey based study		[71]

						Denmark , Belgium)			
Distance daughters (average)	Hours of homecare children	Marginal effect: -10.54, z-value: -3.11	multivariate modelling	Survey of Health, Ageing, and Retirement in Europe (SHARE)	Informal homecare, couples households N=5557	Europe (Austria, Germany , Sweden, The Netherlands, Spain, Italy, France, Denmark , Belgium)	Survey based study		[71]
Employment daughters (average)	Hours of homecare children	Marginal effect: -4.96, z-value: -2.24	multivariate modelling	Survey of Health, Ageing, and Retirement in Europe (SHARE)	Informal homecare, couples households N=5557	Europe (Austria, Germany , Sweden, The Netherlands, Spain, Italy, France, Denmark , Belgium)	Survey based study		[71]
Men, age	Hours of homecare children	Marginal effect: 0.34, z-value: 2.32	multivariate modelling	Survey of Health, Ageing, and Retirement in Europe (SHARE)	Informal homecare, couples households N=5557	Europe (Austria, Germany , Sweden, The Netherlands,	Survey based study		[71]

						Spain, Italy, France, Denmark, Belgium)			
Men, numeracy	Hours of homecare children	Marginal effect: 4.53, z-value: 2.01	multivariate modelling	Survey of Health, Ageing, and Retirement in Europe (SHARE)	Informal homecare, couples households N=5557	Europe (Austria, Germany, Sweden, The Netherlands, Spain, Italy, France, Denmark, Belgium)	Survey based study		[71]
Children	Friends	Marginal effect: -0.49, z-value: -5.44	multivariate modelling	Survey of Health, Ageing, and Retirement in Europe (SHARE)	Informal homecare, couples households N=5557	Europe (Austria, Germany, Sweden, The Netherlands, Spain, Italy, France, Denmark, Belgium)	Survey based study		[71]
Children	Formal care	Marginal effect: -0.23, z-value: -2.89	multivariate modelling	Survey of Health, Ageing, and Retirement in Europe (SHARE)	Informal homecare, couples households N=5557	Europe (Austria, Germany, Sweden,	Survey based study		[71]

						The Netherlands, Spain, Italy, France, Denmark, Belgium)			
Relatives	Friends	Marginal effect: -0.42, z-value: -5.42	multivariate modelling	Survey of Health, Ageing, and Retirement in Europe (SHARE)	Informal homecare, couples households N=5557	Europe (Austria, Germany, Sweden, The Netherlands, Spain, Italy, France, Denmark, Belgium)	Survey based study		[71]
Relatives	Formal care	Marginal effect: -0.29, z-value: -3.11	multivariate modelling	Survey of Health, Ageing, and Retirement in Europe (SHARE)	Informal homecare, couples households N=5557	Europe (Austria, Germany, Sweden, The Netherlands, Spain, Italy, France, Denmark, Belgium)	Survey based study		[71]

Women, years of education	Hours of homecare relatives	Marginal effect: 0.59, z-value: 2.00	multivariate modelling	Survey of Health, Ageing, and Retirement in Europe (SHARE)	Informal homecare, couples households N=5557	Europe (Austria, Germany, Sweden, The Netherlands, Spain, Italy, France, Denmark, Belgium)	Survey based study		[71]
Sociability	Hours of homecare friends	Marginal effect: 0.92, z-value: 2.24	multivariate modelling	Survey of Health, Ageing, and Retirement in Europe (SHARE)	Informal homecare, couples households N=5557	Europe (Austria, Germany, Sweden, The Netherlands, Spain, Italy, France, Denmark, Belgium)	Survey based study		[71]
Has daughters	Hours of homecare friends	Marginal effect: -21.10, z-value: -2.18	multivariate modelling	Survey of Health, Ageing, and Retirement in Europe (SHARE)	Informal homecare, couples households N=5557	Europe (Austria, Germany, Sweden, The Netherlands, Spain, Italy, France,	Survey based study		[71]

						Denmark, Belgium)			
Distance daughters (average)	Hours of homecare friends	Marginal effect: 5.87, z-value: 2.29	multivariate modelling	Survey of Health, Ageing, and Retirement in Europe (SHARE)	Informal homecare, couples households N=5557	Europe (Austria, Germany, Sweden, The Netherlands, Spain, Italy, France, Denmark, Belgium)	Survey based study		[71]
Men, mobility	Hours of homecare friends	Marginal effect: 2.67, z-value: 2.43	multivariate modelling	Survey of Health, Ageing, and Retirement in Europe (SHARE)	Informal homecare, couples households N=5557	Europe (Austria, Germany, Sweden, The Netherlands, Spain, Italy, France, Denmark, Belgium)	Survey based study		[71]
Sociability	Hours of formal homecare	Marginal effect: -1.22, z-value: -3.71	multivariate modelling	Survey of Health, Ageing, and Retirement in Europe (SHARE)	Informal homecare, couples households N=5557	Europe (Austria, Germany, Sweden, The Netherlands,	Survey based study		[71]

						Spain, Italy, France, Denmark, Belgium)			
Men, ADL	Hours of formal homecare	Marginal effect: 3.54, z-value: 3.47	multivariate modelling	Survey of Health, Ageing, and Retirement in Europe (SHARE)	Informal homecare, couples households N=5557	Europe (Austria, Germany, Sweden, The Netherlands, Spain, Italy, France, Denmark, Belgium)	Survey based study		[71]
Women, ADL	Hours of formal homecare	Marginal effect: 4.76, z-value: 4.87	multivariate modelling	Survey of Health, Ageing, and Retirement in Europe (SHARE)	Informal homecare, couples households N=5557	Europe (Austria, Germany, Sweden, The Netherlands, Spain, Italy, France, Denmark, Belgium)	Survey based study		[71]
Unfamiliarity with programs or websites that facilitate	Barriers to health information technology use for out-	n/a	qualitatitve	Survey	N=316 informal caregivers	USA	Survey based study		[103]

out-of-home caregiving	of-home caregiving								
Privacy rules of care recipient's healthcare provider	Barriers to health information technology use for out-of-home caregiving	n/a	qualitatitve	Survey	N=316 informal caregivers	USA	Survey based study		[103]
Insufficient time	Barriers to health information technology use for out-of-home caregiving	n/a	qualitatitve	Survey	N=316 informal caregivers	USA	Survey based study		[103]
Computer/Internet too complicated	Barriers to health information technology use for out-of-home caregiving	n/a	qualitatitve	Survey	N=316 informal caregivers	USA	Survey based study		[103]
Distrust or functional limitations	Barriers to health information technology use for out-of-home caregiving	n/a	qualitatitve	Survey	N=316 informal caregivers	USA	Survey based study		[103]
One or more of the above barriers	Barriers to health information technology use for out-of-home caregiving	n/a	qualitatitve	Survey	N=316 informal caregivers	USA	Survey based study		[103]

Out of home caregiving intensity: low, medium, high	Use of health information technology to support individuals with chronic conditions	Adjuster OR (95% CI): 1.88 (1.01-3.50), 2.39 (1.11-5.15), 3.7 (1.62-8.45)	multivariate analysis.	Survey	N=301 informal caregivers	USA	Survey based study		[103]
Caregiver's comfort with technology: Low, medium, High	Use of health information technology to support individuals with chronic conditions	Adjuster OR (95% CI): 1.23 (0.41-3.67), 2.09 (0.87-5.02), 3.49 (1.34-9.11)	multivariate analysis.	Survey	N=301 informal caregivers	USA	Survey based study		[103]
help care recipient find health information online	Interest in health IT	n/a	qualitatitve	Survey	N=316 informal caregivers	USA	Survey based study		[103]
Help care recipient track his or her health information	Interest in health IT	n/a	qualitatitve	Survey	N=316 informal caregivers	USA	Survey based study		[103]
Help care recipient use health portal or personal HER	Interest in health IT	n/a	qualitatitve	Survey	N=316 informal caregivers	USA	Survey based study		[103]
Help care recipient fill medications or medical supplies online	Interest in health IT	n/a	qualitatitve	Survey	N=316 informal caregivers	USA	Survey based study		[103]

Help care recipient keep track of his or her HER	Interest in health IT	n/a	qualitatitve	Survey	N=316 informal caregivers	USA	Survey based study		[103]
Send email messages to care recipients doctor or other health provider	Interest in health IT	n/a	qualitatitve	Survey	N=316 informal caregivers	USA	Survey based study		[103]
interested in one or more of the above functions	Interest in health IT	n/a	qualitatitve	Survey	N=316 informal caregivers	USA	Survey based study		[103]
Male	Functional ability	Beta: -2.04, p<0.01	linear regression	Survey	N=1234 homecare patients	USA	Survey based study		[104]
Other assistance (type of care)	Functional ability	Beta: -5.80, p<0.01	linear regression	Survey	N=1234 homecare patients	USA	Survey based study		[104]
Cognitive functioning scale	Functional ability	Beta: 1.88, p<0.01	linear regression	Survey	N=1234 homecare patients	USA	Survey based study		[104]
Live alone	Functional ability	Beta: -1.04, p<0.01	linear regression	Survey	N=1234 homecare patients	USA	Survey based study		[104]
Age	Functional ability	Beta: 0.09, p<0.01	linear regression	Survey	N=1234 homecare patients	USA	Survey based study		[104]
Obesity	Functional ability	Beta: 2.12, p<0.01	linear regression	Survey	N=1234 homecare patients	USA	Survey based study		[104]
Frequency of care	Functional ability	Beta: -0.65, p<0.01	linear regression	Survey	N=1234 homecare patients	USA	Survey based study		[104]
Clinical status score, therapeutic self care demand	functional ability score, self care agency	n/a	n/a	Orem's theory of self-care deficit(ref: 9)	n/a	n/a	Theoretical framework		[104]

functional ability score, self care agency	Re-hospitalisation, self-care deficit	n/a	n/a	Orem's theory of self-care deficit(ref: 9)	n/a	n/a	Theoretical framework		[104]
Basic conditions factors (age, gender, high risk characteristics, social environmental factors)	functional ability score, self care agency	n/a	n/a	Orem's theory of self-care deficit(ref: 9)	n/a	n/a	Theoretical framework		[104]
Cogrnitive functioning, power component	functional ability score, self care agency	n/a	n/a	Orem's theory of self-care deficit(ref: 9)	n/a	n/a	Theoretical framework		[104]
Behavioural competence	Quality of Life	n/a	n/a	Lawton's model of quality of life in persons with dementia	n/a	n/a	Theoretical framework		[105]
Psychosocial wellbeing	Quality of Life	n/a	n/a	Lawton's model of quality of life in persons with dementia	n/a	n/a	Theoretical framework		[105]
Perceived quality of life	Quality of Life	n/a	n/a	Lawton's model of quality of life in persons with dementia	n/a	n/a	Theoretical framework		[105]
environmental quality	Quality of Life	n/a	n/a	Lawton's model of quality of life in persons with dementia	n/a	n/a	Theoretical framework		[105]
Client interview instrument (CLINT) Total:	Environmental quality	z=-2.1, P=0.039	independent samples Mann-Whitney U-test	Lawson's model	N=164 patients with alzheimer's disease receiving formal home care	Sweden	Cross-sectional qualitatitive descriptive interview study		[105]

personal interactions, staff doing what you want them to do, staff being honest, hygiene, cleaning, food portions, appreciating meals, overall satisfaction, receiving dementia care, satisfaction with dementia care, quality of care indicators: daily pain, fall the past month, injured when falling, pressure ulcer, weight loss >= 4% the previous year.								
Client interview instrument (CLINT)	Quality of life in Alzheimer's disease	z=-2.8, P=0.006	independe nt samples Mann-	Lawson's model	N=164 patients with alzheimer's disease receiving formal home care	Sweden	Cross-sectional qualitatitive descriptive interview study	[105]

Total: personal interactions, staff doing what you want them to do, staff being honest, hygiene, cleaning, food portions, appreciating meals, overall satisfaction, receiving dementia care, satisfaction with dementia care, quality of care indicators: daily pain, fall the past month, injured when falling, pressure ulcer, weight loss >= 4% the previous year.			Whitney U-test					
Informal homecare (relatives)	Complementary labour: providing	n/a	qualitative interview	Qualitative in-depth interviews	N=118 home support workers	Canada	Cross-sectional qualitative descriptive interview study	[106]

	informational support							
Informal homecare (relatives)	Complementary labour: providing instrumental support	n/a	qualitative interview	Qualitative in-depth interviews	N=118 home support workers	Canada	Cross-sectional qualitatitive descriptive interview study	[106]
Informal homecare (relatives)	Disrupted labour: additional instrumental work (creating messes etc.)	n/a	qualitative interview	Qualitative in-depth interviews	N=118 home support workers	Canada	Cross-sectional qualitatitive descriptive interview study	[106]
Informal homecare (relatives)	Disrupted labour: additional emotional work (disrespectful, rude conflict)	n/a	qualitative interview	Qualitative in-depth interviews	N=118 home support workers	Canada	Cross-sectional qualitatitive descriptive interview study	[106]
Informal homecare (relatives)	If Care plan is too rigid, then can create conflict and disagreement between health workers and relatives.	n/a	qualitative interview	Qualitative in-depth interviews	N=118 home support workers	Canada	Cross-sectional qualitatitive descriptive interview study	[106]
Emphatic awareness	Mitigates conflicts	n/a	qualitative interview	Qualitative in-depth interviews	N=118 home support workers	Canada	Cross-sectional qualitatitive descriptive interview study	[106]
Patient education	Composite quality of life	Beta: 0.356, $p<0.001$	stepwise multiple regression		N=77 Alzheimer's disease patients in community	USA	Survey based study	[107]

	Alzheimer's disease				dwelling, and care-givers				
Geriatric depression scale (patient report)	Composite quality of life Alzheimer's disease	Beta: -0.360,p<0.001	stepwise multiple regression		N=77 Alzheimer's disease patients in community dwelling, and care-givers	USA	Survey based study		[107]
Geriatric depression scale (caregiver report)	Composite quality of life Alzheimer's disease	Beta: -0.326, p<0.01	stepwise multiple regression		N=77 Alzheimer's disease patients in community dwelling, and care-givers	USA	Survey based study		[107]
Activities of daily living (ADL)	Composite quality of life Alzheimer's disease	Beta: -0.281, p<0.01	stepwise multiple regression		N=77 Alzheimer's disease patients in community dwelling, and care-givers	USA	Survey based study		[107]
safety comfort	subjective quality of life	n/a	n/a	n/a	n/a	n/a	Theoretical framework		[64]
functional abilities	subjective quality of life	n/a	n/a	n/a	n/a	n/a	Theoretical framework		[64]
social identity + relations	subjective quality of life	n/a	n/a	n/a	n/a	n/a	Theoretical framework		[64]
psychological well-being	subjective quality of life	n/a	n/a	n/a	n/a	n/a	Theoretical framework		[64]
care as service support	care-related quality of life	n/a	n/a	n/a	n/a	n/a	Theoretical framework		[64]
care as autonomy support	care-related quality of life	n/a	n/a	n/a	n/a	n/a	Theoretical framework		[64]

care as social support	care-related quality of life	n/a	n/a	n/a	n/a	n/a	Theoretical framework		[64]
care as emotional support	care-related quality of life	n/a	n/a	n/a	n/a	n/a	Theoretical framework		[64]
quality of management of resources/finances	quality of management	n/a	n/a	n/a	n/a	n/a	Theoretical framework		[64]
quality of management of work organisation	quality of management	n/a	n/a	n/a	n/a	n/a	Theoretical framework		[64]
quality of management of mission/concepts	quality of management	n/a	n/a	n/a	n/a	n/a	Theoretical framework		[64]
quality of management of cooperation/motivation	quality of management	n/a	n/a	n/a	n/a	n/a	Theoretical framework		[64]
conditions/risks	care-related quality of life	n/a	n/a	n/a	n/a	n/a	Theoretical framework		[64]
life events	care-related quality of life	n/a	n/a	n/a	n/a	n/a	Theoretical framework		[64]
D1: physical dimension	WHO quality of life	n/a	n/a	n/a	n/a	n/a	Theoretical framework		[64]
D2: psychological dimension	WHO quality of life	n/a	n/a	n/a	n/a	n/a	Theoretical framework		[64]

D3: social dimension	WHO quality of life	n/a	n/a	n/a	n/a	n/a	Theoretical framework		[64]
D4: environmental dimension	WHO quality of life	n/a	n/a	n/a	n/a	n/a	Theoretical framework		[64]
Age	Quality of life: WHO dimensions	WHO physical/psychological/social/environment dimensions of which at least one P<0.05	Pearson correlations	WHOQOL-Bref-Scale; Philadelphia Geriatric Centre Moral Scale; and other instrumentation.	Older homecare clients, N=1913 + N=669 + N=83, N=63.	Finland	Survey based study		[64]
Traumatic life changes during past 12 months	Quality of life: WHO dimensions	WHO physical/psychological/social/environment dimensions of which at least one P<0.05	Pearson correlations	WHOQOL-Bref-Scale; Philadelphia Geriatric Centre Moral Scale; and other instrumentation.	Older homecare clients, N=1913 + N=669 + N=83, N=63.	Finland	Survey based study		[64]
Physical-functional dimension: Education (ISCED97)	Quality of life: WHO dimensions	WHO physical/psychological/social/environment dimensions of which at least one P<0.05	Pearson correlations	WHOQOL-Bref-Scale; Philadelphia Geriatric Centre Moral Scale; and other instrumentation.	Older homecare clients, N=1913 + N=669 + N=83, N=63.	Finland	Survey based study		[64]
Physical-functional dimension: Subjective health	Quality of life: WHO dimensions	WHO physical/psychological/social/environment	Pearson correlations	WHOQOL-Bref-Scale; Philadelphia Geriatric Centre Moral Scale; and other instrumentation.	Older homecare clients, N=1913 + N=669 + N=83, N=63.	Finland	Survey based study		[64]

		dimensions of which at least one P<0.05							
Physical-functional dimension: Need of help in ADL-activities/healthcare	Quality of life: WHO dimensions	WHO physical/psychological/social/environment dimensions of which at least one P<0.05	Pearson correlations	WHOQOL-Bref-Scale; Philadelphia Geriatric Centre Moral Scale; and other instrumentation.	Older homecare clients, N=1913 + N=669 + N=83, N=63.	Finland	Survey based study		[64]
Physical-functional dimension: Feeling of self-efficacy	Quality of life: WHO dimensions	WHO physical/psychological/social/environment dimensions of which at least one P<0.05	Pearson correlations	WHOQOL-Bref-Scale; Philadelphia Geriatric Centre Moral Scale; and other instrumentation.	Older homecare clients, N=1913 + N=669 + N=83, N=63.	Finland	Survey based study		[64]
Physical-functional dimension: active in sports	Quality of life: WHO dimensions	WHO physical/psychological/social/environment dimensions of which at least one P<0.05	Pearson correlations	WHOQOL-Bref-Scale; Philadelphia Geriatric Centre Moral Scale; and other instrumentation.	Older homecare clients, N=1913 + N=669 + N=83, N=63.	Finland	Survey based study		[64]
Physical-functional dimension: use of intramural hospital care during the	Quality of life: WHO dimensions	WHO physical/psychological/social/environment dimensions of which at	Pearson correlations	WHOQOL-Bref-Scale; Philadelphia Geriatric Centre Moral Scale; and other instrumentation.	Older homecare clients, N=1913 + N=669 + N=83, N=63.	Finland	Survey based study		[64]

past 12 months		least one P<0.05							
Physical-functional dimension: Visited hospital polyclinics during past 12 months	Quality of life: WHO dimensions	WHO physical/psychological/social/environment dimensions of which at least one P<0.05	Pearson correlations	WHOQOL-Bref-Scale; Philadelphia Geriatric Centre Moral Scale; and other instrumentation.	Older homecare clients, N=1913 + N=669 + N=83, N=63.	Finland	Survey based study		[64]
Psychological dimension: Positive attitude toward own ageing (PGCMS)	Quality of life: WHO dimensions	WHO physical/psychological/social/environment dimensions of which at least one P<0.05	Pearson correlations	WHOQOL-Bref-Scale; Philadelphia Geriatric Centre Moral Scale; and other instrumentation.	Older homecare clients, N=1913 + N=669 + N=83, N=63.	Finland	Survey based study		[64]
Psychological dimension: Less lonely (PGCMS)	Quality of life: WHO dimensions	WHO physical/psychological/social/environment dimensions of which at least one P<0.05	Pearson correlations	WHOQOL-Bref-Scale; Philadelphia Geriatric Centre Moral Scale; and other instrumentation.	Older homecare clients, N=1913 + N=669 + N=83, N=63.	Finland	Survey based study		[64]
Psychological dimension: Less agitation (PGCMS)	Quality of life: WHO dimensions	WHO physical/psychological/social/environment dimensions of which at least one P<0.05	Pearson correlations	WHOQOL-Bref-Scale; Philadelphia Geriatric Centre Moral Scale; and other instrumentation.	Older homecare clients, N=1913 + N=669 + N=83, N=63.	Finland	Survey based study		[64]

Psychological dimension: Feeling of self-determination	Quality of life: WHO dimensions	WHO physical/psychological/social/environment dimensions of which at least one P<0.05	Pearson correlations	WHOQOL-Bref-Scale; Philadelphia Geriatric Centre Moral Scale; and other instrumentation.	Older homecare clients, N=1913 + N=669 + N=83, N=63.	Finland	Survey based study		[64]
Psychological dimension: feels not bored	Quality of life: WHO dimensions	WHO physical/psychological/social/environment dimensions of which at least one P<0.05	Pearson correlations	WHOQOL-Bref-Scale; Philadelphia Geriatric Centre Moral Scale; and other instrumentation.	Older homecare clients, N=1913 + N=669 + N=83, N=63.	Finland	Survey based study		[64]
Social dimension: intensive social relationships	Quality of life: WHO dimensions	WHO physical/psychological/social/environment dimensions of which at least one P<0.05	Pearson correlations	WHOQOL-Bref-Scale; Philadelphia Geriatric Centre Moral Scale; and other instrumentation.	Older homecare clients, N=1913 + N=669 + N=83, N=63.	Finland	Survey based study		[64]
Social dimension: Lives alone	Quality of life: WHO dimensions	WHO physical/psychological/social/environment dimensions of which at least one P<0.05	Pearson correlations	WHOQOL-Bref-Scale; Philadelphia Geriatric Centre Moral Scale; and other instrumentation.	Older homecare clients, N=1913 + N=669 + N=83, N=63.	Finland	Survey based study		[64]

Social dimension: Gives regular informal help/care to someone else	Quality of life: WHO dimensions	WHO physical/psychological/social/environment dimensions of which at least one $P<0.05$	Pearson correlations	WHOQOL-Bref-Scale; Philadelphia Geriatric Centre Moral Scale; and other instrumentation.	Older homecare clients, N=1913 + N=669 + N=83, N=63.	Finland	Survey based study		[64]
Environmental dimension: Net income	Quality of life: WHO dimensions	WHO physical/psychological/social/environment dimensions of which at least one $P<0.05$	Pearson correlations	WHOQOL-Bref-Scale; Philadelphia Geriatric Centre Moral Scale; and other instrumentation.	Older homecare clients, N=1913 + N=669 + N=83, N=63.	Finland	Survey based study		[64]
Environmental dimension: Need of help in IAD-activities	Quality of life: WHO dimensions	WHO physical/psychological/social/environment dimensions of which at least one $P<0.05$	Pearson correlations	WHOQOL-Bref-Scale; Philadelphia Geriatric Centre Moral Scale; and other instrumentation.	Older homecare clients, N=1913 + N=669 + N=83, N=63.	Finland	Survey based study		[64]
Environmental dimension: Poor access to services and amenities	Quality of life: WHO dimensions	WHO physical/psychological/social/environment dimensions of which at least one $P<0.05$	Pearson correlations	WHOQOL-Bref-Scale; Philadelphia Geriatric Centre Moral Scale; and other instrumentation.	Older homecare clients, N=1913 + N=669 + N=83, N=63.	Finland	Survey based study		[64]

Environmental dimension: Barrier-free flat/house	Quality of life: WHO dimensions	WHO physical/psychological/social/environment dimensions of which at least one P<0.05	Pearson correlations	WHOQOL-Bref-Scale; Philadelphia Geriatric Centre Moral Scale; and other instrumentation.	Older homecare clients, N=1913 + N=669 + N=83, N=63.	Finland	Survey based study		[64]
Environmental dimension: Uses regularly formal homecare	Quality of life: WHO dimensions	WHO physical/psychological/social/environment dimensions of which at least one P<0.05	Pearson correlations	WHOQOL-Bref-Scale; Philadelphia Geriatric Centre Moral Scale; and other instrumentation.	Older homecare clients, N=1913 + N=669 + N=83, N=63.	Finland	Survey based study		[64]
Environmental dimension: receives informal help from family	Quality of life: WHO dimensions	WHO physical/psychological/social/environment dimensions of which at least one P<0.05	Pearson correlations	WHOQOL-Bref-Scale; Philadelphia Geriatric Centre Moral Scale; and other instrumentation.	Older homecare clients, N=1913 + N=669 + N=83, N=63.	Finland	Survey based study		[64]
Environmental dimension: uses private care services	Quality of life: WHO dimensions	WHO physical/psychological/social/environment dimensions of which at least one P<0.05	Pearson correlations	WHOQOL-Bref-Scale; Philadelphia Geriatric Centre Moral Scale; and other instrumentation.	Older homecare clients, N=1913 + N=669 + N=83, N=63.	Finland	Survey based study		[64]

Environmental dimension: Gets enough help in right things	Quality of life: WHO dimensions	WHO physical/psychological/social/environment dimensions of which at least one P<0.05	Pearson correlations	WHOQOL-Bref-Scale; Philadelphia Geriatric Centre Moral Scale; and other instrumentation.	Older homecare clients, N=1913 + N=669 + N=83, N=63.	Finland	Survey based study		[64]
Environmental dimension: Satisfied with help received	Quality of life: WHO dimensions	WHO physical/psychological/social/environment dimensions of which at least one P<0.05	Pearson correlations	WHOQOL-Bref-Scale; Philadelphia Geriatric Centre Moral Scale; and other instrumentation.	Older homecare clients, N=1913 + N=669 + N=83, N=63.	Finland	Survey based study		[64]
patient/clinician perception of effectiveness	sustainable home tele-homecare programs	n/a	Qualitative literature review	n/a	Homecare	n/a	Qualitative review		[108]
tailoring to patients	sustainable home tele-homecare programs	n/a	Qualitative literature review	n/a	Homecare	n/a	Qualitative review		[108]
nurse-patient communication and collaboration	sustainable home tele-homecare programs	n/a	Qualitative literature review	n/a	Homecare	n/a	Qualitative review		[108]
interprofessional communication and collaboration	sustainable home tele-homecare programs	n/a	Qualitative literature review	n/a	Homecare	n/a	Qualitative review		[108]

organisational process and culture	sustainable home tele-homecare programs	n/a	Qualitative literature review	n/a	Homecare	n/a	Qualitative review		[108]
quality of tele-homecare technology	sustainable home tele-homecare programs	n/a	Qualitative literature review	n/a	Homecare	n/a	Qualitative review		[108]
lack of training and orientation	lack of understanding of tele-homecare use to improve clinical and behavioural outcomes	n/a	Qualitative literature review	n/a	Homecare	n/a	Qualitative review		[108]
tele-homecare	promote daily self-monitoring activities	n/a	Qualitative literature review	n/a	Homecare	n/a	Qualitative review		[108]
uncertainty about evidence	barrier to tele-homecare	n/a	Qualitative literature review	n/a	Homecare	n/a	Qualitative review		[108]
tele-homecare	increased access to clinicians	n/a	Qualitative literature review	n/a	Homecare	n/a	Qualitative review		[108]
increased access to clinicians	patient sense of security and reassurance	n/a	Qualitative literature review	n/a	Homecare	n/a	Qualitative review		[108]
tele-homecare	interprofessional collaboration	n/a	Qualitative literature review	n/a	Homecare	n/a	Qualitative review		[108]
lack of interoperability	barrier to tele-homecare	n/a	Qualitative literature review	n/a	Homecare	n/a	Qualitative review		[108]

top-down decision making	adoption of tele-homecare	n/a	Qualitative literature review	n/a	Homecare	n/a	Qualitative review		[108]
top-down decision making regarding tele-homecare	professional resistance	n/a	Qualitative literature review	n/a	Homecare	n/a	Qualitative review		[108]
poor home health workflow adaptability to tele-homecare	increased workload	n/a	Qualitative literature review	n/a	Homecare	n/a	Qualitative review		[108]
non-innovative tele-homecare	unsustainable implementation of tele-homecare	n/a	Qualitative literature review	n/a	Homecare	n/a	Qualitative review		[108]
Performance expectancy	Intention to use technology	n/a	Mixed method	Unified theory of acceptance and uses of technology (UTAUT) model	Homecare	USA	Mixed method		[109]
Effort expectancy	Intention to use technology	n/a	Mixed method	Unified theory of acceptance and uses of technology (UTAUT) model	Homecare	USA	Mixed method		[109]
Social influence	Intention to use technology	n/a	Mixed method	Unified theory of acceptance and uses of technology (UTAUT) model	Homecare	USA	Mixed method		[109]
Facilitating conditions	Intention to use technology	n/a	Mixed method	Unified theory of acceptance and uses of technology (UTAUT) model	Homecare	USA	Mixed method		[109]

Intention to use technology	Individual behaviour	n/a	Mixed method	Unified theory of acceptance and uses of technology (UTAUT) model	Homecare	USA	Mixed method		[109]
Facilitating conditions	Individual behaviour	n/a	Mixed method	Unified theory of acceptance and uses of technology (UTAUT) model	Homecare	USA	Mixed method		[109]
Sense of security	Nurses perceived benefits of using telehealth for heart failure	n/a	Questionnaire and analysis	UTAUT model	N=31 nurses and N=4 patients in homecare setting	USA	Questionnaire based study		[109]
Objective and timely patient status	Nurses perceived benefits of using telehealth for heart failure	n/a	Questionnaire and analysis	UTAUT model	N=31 nurses and N=4 patients in homecare setting	USA	Questionnaire based study		[109]
Daily patient monitoring	Nurses perceived benefits of using telehealth for heart failure	n/a	Questionnaire and analysis	UTAUT model	N=31 nurses and N=4 patients in homecare setting	USA	Questionnaire based study		[109]
Patient self-monitoring and accountability	Nurses perceived benefits of using telehealth for heart failure	n/a	Questionnaire and analysis	UTAUT model	N=31 nurses and N=4 patients in homecare setting	USA	Questionnaire based study		[109]

Sense of security/peace of mind	Patient perceived benefits of using telehealth for heart failure	n/a	Questionnaire and analysis	UTAUT model	N=31 nurses and N=4 patients in homecare setting	USA	Questionnaire based study		[109]
Convenience of home monitoring	Patient perceived benefits of using telehealth for heart failure	n/a	Questionnaire and analysis	UTAUT model	N=31 nurses and N=4 patients in homecare setting	USA	Questionnaire based study		[109]
Daily monitoring	Patient perceived benefits of using telehealth for heart failure	n/a	Questionnaire and analysis	UTAUT model	N=31 nurses and N=4 patients in homecare setting	USA	Questionnaire based study		[109]
Reassurance to family members	Patient perceived benefits of using telehealth for heart failure	n/a	Questionnaire and analysis	UTAUT model	N=31 nurses and N=4 patients in homecare setting	USA	Questionnaire based study		[109]
Increased workload	Nurses perceived disadvantages of using telehealth for heart failure	n/a	Questionnaire and analysis	UTAUT model	N=31 nurses and N=4 patients in homecare setting	USA	Questionnaire based study		[109]
disruptive to nurse and	Nurses perceived disadvantage	n/a	Questionnaire and analysis	UTAUT model	N=31 nurses and N=4 patients in homecare setting	USA	Questionnaire based study		[109]

patient routine	s of using telehealth for heart failure								
patient dependence on telehealth equipment	Nurses perceived disadvantage s of using telehealth for heart failure	n/a	Questionna ire and analysis	UTAUT model	N=31 nurses and N=4 patients in homecare setting	USA	Questionnaire based study		[109]
causative patient anxiety	Nurses perceived disadvantage s of using telehealth for heart failure	n/a	Questionna ire and analysis	UTAUT model	N=31 nurses and N=4 patients in homecare setting	USA	Questionnaire based study		[109]
compromise d patient safety	Nurses perceived disadvantage s of using telehealth for heart failure	n/a	Questionna ire and analysis	UTAUT model	N=31 nurses and N=4 patients in homecare setting	USA	Questionnaire based study		[109]
Intrusive	Patient perceived disadvantage s of using telehealth for heart failure	n/a	Questionna ire and analysis	UTAUT model	N=31 nurses and N=4 patients in homecare setting	USA	Questionnaire based study		[109]
Disruptive to routine	Patient perceived disadvantage s of using telehealth	n/a	Questionna ire and analysis	UTAUT model	N=31 nurses and N=4 patients in homecare setting	USA	Questionnaire based study		[109]

	for heart failure								
time-consuming	Patient perceived disadvantages of using telehealth for heart failure	n/a	Questionnaire and analysis	UTAUT model	N=31 nurses and N=4 patients in homecare setting	USA	Questionnaire based study		[109]
causative anxiety	Patient perceived disadvantages of using telehealth for heart failure	n/a	Questionnaire and analysis	UTAUT model	N=31 nurses and N=4 patients in homecare setting	USA	Questionnaire based study		[109]
compromised safety	Patient perceived disadvantages of using telehealth for heart failure	n/a	Questionnaire and analysis	UTAUT model	N=31 nurses and N=4 patients in homecare setting	USA	Questionnaire based study		[109]
Management mandate	Nurses: facilitators to telehealth	n/a	Questionnaire and analysis	UTAUT model	N=31 nurses and N=4 patients in homecare setting	USA	Questionnaire based study		[109]
Patient fear of hospitalisation	Nurses: facilitators to telehealth	n/a	Questionnaire and analysis	UTAUT model	N=31 nurses and N=4 patients in homecare setting	USA	Questionnaire based study		[109]
Patient characteristics- younger age, new diagnosis	Nurses: facilitators to telehealth	n/a	Questionnaire and analysis	UTAUT model	N=31 nurses and N=4 patients in homecare setting	USA	Questionnaire based study		[109]
Caregiver support	Nurses: facilitators to telehealth	n/a	Questionnaire and analysis	UTAUT model	N=31 nurses and N=4 patients in homecare setting	USA	Questionnaire based study		[109]

Physician buy-in	Nurses: facilitators to telehealth	n/a	Questionnaire and analysis	UTAUT model	N=31 nurses and N=4 patients in homecare setting	USA	Questionnaire based study		[109]
Equipment user-friendliness	Patients: facilitators to telehealth	n/a	Questionnaire and analysis	UTAUT model	N=31 nurses and N=4 patients in homecare setting	USA	Questionnaire based study		[109]
Fear of hospitalisation	Patients: facilitators to telehealth	n/a	Questionnaire and analysis	UTAUT model	N=31 nurses and N=4 patients in homecare setting	USA	Questionnaire based study		[109]
Unstable disease status	Patients: facilitators to telehealth	n/a	Questionnaire and analysis	UTAUT model	N=31 nurses and N=4 patients in homecare setting	USA	Questionnaire based study		[109]
caregiver support	Patients: facilitators to telehealth	n/a	Questionnaire and analysis	UTAUT model	N=31 nurses and N=4 patients in homecare setting	USA	Questionnaire based study		[109]
Physician buy-in	Patients: facilitators to telehealth	n/a	Questionnaire and analysis	UTAUT model	N=31 nurses and N=4 patients in homecare setting	USA	Questionnaire based study		[109]
Oldest age-group patients	Nurses: barriers to use telehealth	n/a	Questionnaire and analysis	UTAUT model	N=31 nurses and N=4 patients in homecare setting	USA	Questionnaire based study		[109]
lack of nurse orientation	Nurses: barriers to use telehealth	n/a	Questionnaire and analysis	UTAUT model	N=31 nurses and N=4 patients in homecare setting	USA	Questionnaire based study		[109]
lack of perceived usefulness	Nurses: barriers to use telehealth	n/a	Questionnaire and analysis	UTAUT model	N=31 nurses and N=4 patients in homecare setting	USA	Questionnaire based study		[109]
short episode of care	Nurses: barriers to use telehealth	n/a	Questionnaire and analysis	UTAUT model	N=31 nurses and N=4 patients in homecare setting	USA	Questionnaire based study		[109]
lack of trust in equipment	Nurses: barriers to use telehealth	n/a	Questionnaire and analysis	UTAUT model	N=31 nurses and N=4 patients in homecare setting	USA	Questionnaire based study		[109]

preference of in-person assessment	Nurses: barriers to use telehealth	n/a	Questionnaire and analysis	UTAUT model	N=31 nurses and N=4 patients in homecare setting	USA	Questionnaire based study		[109]
patient/caregiver with anxious personality	Nurses: barriers to use telehealth	n/a	Questionnaire and analysis	UTAUT model	N=31 nurses and N=4 patients in homecare setting	USA	Questionnaire based study		[109]
patients lacking English language ability	Nurses: barriers to use telehealth	n/a	Questionnaire and analysis	UTAUT model	N=31 nurses and N=4 patients in homecare setting	USA	Questionnaire based study		[109]
certain comorbidities	Nurses: barriers to use telehealth	n/a	Questionnaire and analysis	UTAUT model	N=31 nurses and N=4 patients in homecare setting	USA	Questionnaire based study		[109]
chronic-ness of disease	Nurses: barriers to use telehealth	n/a	Questionnaire and analysis	UTAUT model	N=31 nurses and N=4 patients in homecare setting	USA	Questionnaire based study		[109]
Lack of interagency collaboration	Nurses: barriers to use telehealth	n/a	Questionnaire and analysis	UTAUT model	N=31 nurses and N=4 patients in homecare setting	USA	Questionnaire based study		[109]
Equipment malfunction	Patients: barriers to use telehealth	n/a	Questionnaire and analysis	UTAUT model	N=31 nurses and N=4 patients in homecare setting	USA	Questionnaire based study		[109]
Caregiver opposition	Patients: barriers to use telehealth	n/a	Questionnaire and analysis	UTAUT model	N=31 nurses and N=4 patients in homecare setting	USA	Questionnaire based study		[109]
lack of perceived usefulness	Patients: barriers to use telehealth	n/a	Questionnaire and analysis	UTAUT model	N=31 nurses and N=4 patients in homecare setting	USA	Questionnaire based study		[109]

Inability to prevent hospitalisation	Patients: barriers to use telehealth	n/a	Questionnaire and analysis	UTAUT model	N=31 nurses and N=4 patients in homecare setting	USA	Questionnaire based study		[109]
nurse reluctance to use equipment	Patients: barriers to use telehealth	n/a	Questionnaire and analysis	UTAUT model	N=31 nurses and N=4 patients in homecare setting	USA	Questionnaire based study		[109]
preference of in-person assessment	Patients: barriers to use telehealth	n/a	Questionnaire and analysis	UTAUT model	N=31 nurses and N=4 patients in homecare setting	USA	Questionnaire based study		[109]
anxious personality	Patients: barriers to use telehealth	n/a	Questionnaire and analysis	UTAUT model	N=31 nurses and N=4 patients in homecare setting	USA	Questionnaire based study		[109]
Private personality	Patients: barriers to use telehealth	n/a	Questionnaire and analysis	UTAUT model	N=31 nurses and N=4 patients in homecare setting	USA	Questionnaire based study		[109]
certain comorbidities	Patients: barriers to use telehealth	n/a	Questionnaire and analysis	UTAUT model	N=31 nurses and N=4 patients in homecare setting	USA	Questionnaire based study		[109]
Physical fragility	Patients: barriers to use telehealth	n/a	Questionnaire and analysis	UTAUT model	N=31 nurses and N=4 patients in homecare setting	USA	Questionnaire based study		[109]
Stable disease	Patients: barriers to use telehealth	n/a	Questionnaire and analysis	UTAUT model	N=31 nurses and N=4 patients in homecare setting	USA	Questionnaire based study		[109]
Physician unfamiliarity with telehealth	Patients: barriers to use telehealth	n/a	Questionnaire and analysis	UTAUT model	N=31 nurses and N=4 patients in homecare setting	USA	Questionnaire based study		[109]
ICT	reduce workload	n/a	qualitative interview	n/a	Personnell in homecare	Sweden	Interview based study		[110]

	and lead times, increase efficiency in work routines								
ICT	improve communications	n/a	qualitative interview	n/a	Personnell in homecare	Sweden	Interview based study		[110]
ICT	increase quality of services	n/a	qualitative interview	n/a	Personnell in homecare	Sweden	Interview based study		[110]
ICT	support customer-adapted care processes	n/a	qualitative interview	n/a	Personnell in homecare	Sweden	Interview based study		[110]
lacking knowledge in computers and technology	barrier to implementing ICT	n/a	qualitative interview	n/a	Personnell in homecare	Sweden	Interview based study		[110]
involvement in development and implementation	facilitator to implementing ICT	n/a	qualitative interview	n/a	Personnell in homecare	Sweden	Interview based study		[110]
ICT	specialisation	n/a	qualitative interview	n/a	Personnell in homecare	Sweden	Interview based study		[110]

Ref: reference; n/a: not applicable.
