# Supplementary material for: Investigating the Connections Between Delivery of Care, Reablement, Workload, and Organizational Factors in Home Care Services: Mixed Methods Study
Source: JMIR Hum Factors. 2023 Jun 30;10:e42283. doi: 10.2196/42283 (PMC10365606; doi:10.2196/42283)
Supplement: Multimedia Appendix 3 [file humanfactors_v10i1e42283_app3.pdf]

Table S5. Causal loop diagram – elements, category and social network analysis metrics.

Label	Category	betweenness	closeness	degree	eigenvector	indegree	metrics::last	micmac exposure	micmac influence	outdegree	reach	reach-efficiency	size
physical status	homecare user	0.008	0.180	2	0.012	2	0.840	0.840	0.808	1	0.123	0.041	3
person-centred care	organisation	0.024	0.176	6	0.007	5	0.888	0.888	0.776	1	0.074	0.011	7
instrumental activities of daily living (iADL) needs	homecare user	0.015	0.201	3	0.036	3	0.751	0.751	0.810	2	0.139	0.035	4
perceived work overload	stress	0.037	0.171	3	0.003	2	0.810	0.810	0.831	1	0.066	0.016	4
sense homecare user suffering	homecare staff	0.009	0.145	3	0.005	1	0.891	0.891	0.844	2	0.041	0.010	4
likelihood of institutional care	homecare user	0.008	0.021	3	0.008	1	0.883	0.883	0.000	2	0.033	0.008	4
homecare staff-user adoption of technology	homecare user	0.073	0.190	6	0.007	4	0.855	0.855	0.788	4	0.082	0.012	7
time spent in care	homecare user	0.006	0.132	2	0.000	1	0.000	0.000	0.814	1	0.041	0.014	3
mastery of work and professional competence	homecare staff	0.018	0.232	6	0.000	2	0.819	0.819	0.766	4	0.172	0.025	7
homecare user influence	homecare user	0.015	0.171	3	0.005	1	0.891	0.891	0.843	2	0.049	0.012	4
parasympatic nerve system activation	homecare staff	0.010	0.133	3	0.000	2	0.718	0.718	0.764	1	0.041	0.010	4
other relatives, neighbours and friends	social support	0.000	0.149	1	0.000	0	0.000	0.000	0.811	1	0.041	0.020	2
quality of supervision	organisation	0.012	0.205	8	0.000	3	0.000	0.000	0.868	6	0.107	0.012	9
social needs	homecare user	0.002	0.195	2	0.034	2	0.850	0.850	0.870	1	0.131	0.044	3
workload	homecare staff	0.078	0.228	7	0.011	3	0.851	0.851	0.824	5	0.148	0.018	8
sense homecare user needs	homecare staff	0.009	0.145	3	0.005	1	0.891	0.891	0.844	2	0.041	0.010	4
recovery	homecare staff	0.010	0.122	3	0.000	1	0.892	0.892	0.799	2	0.033	0.008	4
empowerment	homecare user	0.002	0.168	2	0.001	1	0.861	0.861	0.711	1	0.041	0.014	3
burnout	homecare staff	0.029	0.183	4	0.000	2	0.830	0.830	0.900	2	0.057	0.011	5
provision of care and services	homecare staff	0.196	0.268	12	0.042	6	0.731	0.731	0.751	9	0.254	0.020	13
boredom or underload	stress	0.037	0.171	3	0.003	2	0.810	0.810	0.831	1	0.066	0.016	4
equipment problems	organisation	0.004	0.169	2	0.000	1	0.819	0.819	0.830	1	0.057	0.019	3

informational needs	homecare user	0.002	0.195	2	0.034	2	0.850	0.850	0.870	1	0.131	0.044	3
quality of life of informal caregiver	social support	0.037	0.147	6	0.005	5	0.802	0.802	0.811	1	0.041	0.006	7
control over decision making	homecare staff	0.037	0.206	6	0.001	4	0.815	0.815	0.805	4	0.107	0.015	7
acceptable living environment	homecare user	0.000	0.159	2	0.010	1	0.635	0.635	0.759	2	0.041	0.014	3
ability to cope	stress	0.065	0.125	9	0.023	8	0.928	0.928	0.742	1	0.041	0.004	10
coordination with other services	organisation	0.000	0.164	2	0.002	1	0.777	0.777	1.000	2	0.049	0.016	3
staff shortage	organisation	0.049	0.156	3	0.000	1	0.881	0.881	0.923	2	0.041	0.010	4
job involvement	homecare staff	0.162	0.237	11	0.018	9	0.984	0.984	0.870	4	0.180	0.015	12
commitment to organisation	homecare staff	0.028	0.185	6	0.001	4	0.805	0.805	0.876	2	0.074	0.011	7
job satisfaction	homecare staff	0.102	0.207	12	0.015	9	0.897	0.897	0.819	5	0.090	0.007	13
support provided by others	social support	0.000	0.126	1	0.000	0	0.000	0.000	0.924	1	0.025	0.012	2
ability to follow conscience	homecare staff	0.023	0.195	4	0.001	2	0.860	0.860	0.836	2	0.107	0.021	5
trust in homecare staff	homecare user	0.022	0.219	3	0.034	3	0.942	0.942	0.904	2	0.180	0.045	4
years of work experience	homecare staff	0.004	0.173	2	0.000	1	0.000	0.000	0.884	1	0.049	0.016	3
emphasis on cost-effectiveness	organisation	0.000	0.177	3	0.000	0	0.000	0.000	0.891	3	0.066	0.016	4
experience in care	social support	0.000	0.126	1	0.000	0	0.000	0.000	0.924	1	0.025	0.012	2
training and specialisation	homecare staff	0.024	0.204	4	0.002	2	0.777	0.777	0.679	3	0.098	0.020	5
social isolation	homecare user	0.018	0.200	3	0.005	2	0.918	0.918	0.710	2	0.139	0.035	4
county care capacity	societal level	0.000	0.144	1	0.000	0	0.000	0.000	0.821	1	0.033	0.016	2
confirming homecare staff - user/family communication and relationship	homecare staff	0.137	0.245	12	0.018	6	0.809	0.809	0.895	7	0.180	0.014	13
continuity of care	homecare staff	0.033	0.197	3	0.012	2	0.813	0.813	0.870	1	0.139	0.035	4
family-work conflict	homecare staff	0.042	0.154	6	0.000	4	0.780	0.780	0.895	2	0.049	0.007	7
positive challenges at work	homecare staff	0.031	0.202	7	0.001	3	0.910	0.910	0.912	4	0.098	0.012	8
mental status	homecare user	0.017	0.188	3	0.012	2	0.840	0.840	0.813	2	0.139	0.035	4
healthcare spending	societal level	0.000	0.000	1	0.002	1	0.819	0.819	0.000	0	0.008	0.004	2

spouse or partner	social support	0.000	0.149	1	0.000	0	0.000	0.000	0.811	1	0.041	0.020	2
stress of conscience	homecare staff	0.067	0.166	5	0.030	3	0.782	0.782	0.777	2	0.066	0.011	6
work pace	homecare staff	0.000	0.151	2	0.003	1	0.952	0.952	0.734	1	0.033	0.011	3
self-perceived health of informal caregiver	social support	0.013	0.124	2	0.008	1	0.883	0.883	0.924	1	0.025	0.008	3
suitability of care	homecare staff	0.000	0.197	2	0.011	1	0.896	0.896	0.870	1	0.139	0.046	3
organisational slack	organisation	0.001	0.140	2	0.000	1	0.000	0.000	0.879	1	0.025	0.008	3
stigmatisation towards profession	societal level	0.000	0.144	1	0.000	0	0.000	0.000	0.821	1	0.033	0.016	2
social support	homecare staff	0.000	0.128	1	0.000	0	0.000	0.000	0.811	1	0.033	0.016	2
learning demands	homecare staff	0.003	0.126	2	0.000	1	0.845	0.845	0.742	1	0.041	0.014	3
informal care of homecare user	social support	0.030	0.198	2	0.001	1	0.768	0.768	0.870	1	0.139	0.046	3
fair leadership	organisation	0.000	0.157	1	0.000	0	0.000	0.000	0.837	1	0.066	0.033	2
adverse events or progression of disease	homecare user	0.047	0.164	5	0.029	2	0.804	0.804	0.917	3	0.066	0.011	6
support from superior	organisation	0.000	0.174	2	0.000	0	0.000	0.000	0.772	2	0.082	0.027	3
support from coworkers	organisation	0.000	0.143	2	0.000	1	0.000	0.000	0.761	1	0.041	0.014	3
children	social support	0.000	0.149	1	0.000	0	0.000	0.000	0.811	1	0.041	0.020	2
shift work	organisation	0.000	0.128	1	0.000	0	0.000	0.000	0.811	1	0.033	0.016	2
social support network of homecare user	social support	0.072	0.188	9	0.003	7	0.759	0.759	0.806	3	0.057	0.006	10
complexity of work tasks	homecare staff	0.025	0.189	6	0.001	3	0.869	0.869	0.771	4	0.090	0.013	7
informal caregiver stress	social support	0.015	0.109	2	0.029	1	0.804	0.804	0.790	1	0.025	0.008	3
decision demands	homecare staff	0.037	0.172	4	0.004	3	0.854	0.854	0.804	2	0.074	0.015	5
homecare staff health and safety risk	homecare staff	0.011	0.109	3	0.016	2	0.793	0.793	0.703	1	0.025	0.006	4
sympatic nerve system activation	homecare staff	0.010	0.133	3	0.000	2	0.718	0.718	0.764	1	0.041	0.010	4
empowering leadership	organisation	0.008	0.234	4	0.000	1	0.000	0.000	0.915	4	0.164	0.033	5
female	social support	0.000	0.149	1	0.000	0	0.000	0.000	0.811	1	0.041	0.020	2
county per capita income	societal level	0.000	0.144	1	0.000	0	0.000	0.000	0.821	1	0.033	0.016	2
acceptability of care	homecare staff	0.000	0.197	2	0.011	1	0.896	0.896	0.870	1	0.139	0.046	3

activities of daily living (ADL) needs	homecare user	0.002	0.195	2	0.034	2	0.850	0.850	0.870	1	0.131	0.044	3
working with sophisticated technology	organisation	0.029	0.169	3	0.002	1	0.777	0.777	0.790	2	0.066	0.016	4
user engagement in development and implementation of technology	organisation	0.036	0.146	4	0.005	3	0.891	0.891	0.791	1	0.049	0.010	5
turnover intention	homecare staff	0.081	0.170	4	0.004	2	0.740	0.740	0.702	2	0.057	0.011	5
accessibility of care	homecare staff	0.000	0.197	2	0.011	1	0.896	0.896	0.870	1	0.139	0.046	3
emotional needs	homecare user	0.002	0.195	2	0.034	2	0.850	0.850	0.870	1	0.131	0.044	3
quality of care	homecare staff	0.019	0.208	4	0.002	2	0.912	0.912	0.944	2	0.148	0.030	5
quantitative demands	homecare staff	0.012	0.191	4	0.004	2	0.915	0.915	0.899	2	0.082	0.016	5
distance of relatives	social support	0.000	0.149	1	0.000	0	0.000	0.000	0.811	1	0.041	0.020	2
treatment-related needs	homecare user	0.002	0.195	2	0.034	2	0.850	0.850	0.870	1	0.131	0.044	3
ability to communicate	homecare user	0.039	0.191	5	0.007	4	0.949	0.949	0.845	2	0.098	0.016	6
job demand	stress	0.019	0.153	6	0.004	4	0.880	0.880	0.814	3	0.041	0.006	7
control	stress	0.064	0.153	4	0.007	2	0.868	0.868	0.814	3	0.041	0.008	5
functional ability and autonomy	homecare user	0.082	0.270	14	0.017	4	0.782	0.782	0.750	14	0.205	0.014	15
high or misaligned expectations	homecare user	0.000	0.012	1	0.000	0	0.000	0.000	0.000	1	0.025	0.012	2
self-efficacy of homecare staff	homecare staff	0.000	0.159	1	0.000	0	0.000	0.000	0.785	1	0.049	0.025	2
age of homecare user	homecare user	0.000	0.115	1	0.000	0	0.000	0.000	0.891	1	0.025	0.012	2
recognition	organisation	0.001	0.157	3	0.000	2	1.000	1.000	0.855	1	0.057	0.014	4
inadequate income	organisation	0.002	0.158	2	0.000	1	0.000	0.000	0.855	1	0.057	0.019	3
overtime and unscheduled work	organisation	0.026	0.127	2	0.000	1	0.919	0.919	0.811	1	0.033	0.011	3
county level unemployment	societal level	0.000	0.144	1	0.000	0	0.000	0.000	0.821	1	0.033	0.016	2
moral burden	homecare staff	0.006	0.135	4	0.003	3	0.892	0.892	0.903	1	0.033	0.007	5
ability to balance needs of homecare user and their families	homecare staff	0.012	0.152	4	0.003	3	0.882	0.882	0.810	1	0.033	0.007	5

personal life demands	homecare staff	0.000	0.128	1	0.000	0	0.000	0.000	0.811	1	0.033	0.016	2
long-term physiological health	homecare staff	0.025	0.164	6	0.000	3	0.884	0.884	0.734	3	0.049	0.007	7
mental health	homecare staff	0.016	0.175	6	0.000	2	0.897	0.897	0.718	4	0.066	0.009	7
work-life balance	homecare staff	0.012	0.106	2	0.000	1	0.735	0.735	0.737	1	0.033	0.011	3
satisfaction with care	homecare user	0.000	0.008	3	0.045	3	0.785	0.785	0.000	1	0.016	0.004	4
number of care recipients per carer	organisation	0.020	0.169	3	0.000	2	0.919	0.919	0.830	1	0.057	0.014	4
positive social climate	organisation	0.014	0.178	5	0.000	2	0.851	0.851	0.856	3	0.074	0.012	6
language fluency	homecare user	0.000	0.152	1	0.000	0	0.000	0.000	0.898	1	0.033	0.016	2
distress	stress	0.078	0.234	8	0.002	2	0.899	0.899	0.953	6	0.139	0.015	9
availability of care	homecare staff	0.000	0.197	2	0.011	1	0.896	0.896	0.870	1	0.139	0.046	3
role clarity	organisation	0.001	0.167	3	0.000	1	0.000	0.000	0.767	2	0.066	0.016	4
self-efficacy of homecare user	homecare user	0.000	0.169	1	0.000	0	0.000	0.000	0.711	1	0.041	0.020	2
sickness absence	homecare staff	0.000	0.000	3	0.000	3	0.784	0.784	0.000	0	0.008	0.002	4
job uncertainties	organisation	0.000	0.144	1	0.000	0	0.000	0.000	0.821	1	0.033	0.016	2
language proficiency	homecare staff	0.000	0.178	1	0.000	0	0.000	0.000	0.776	1	0.074	0.037	2
perceived usefulness of technology	organisation	0.000	0.125	1	0.000	0	0.000	0.000	0.815	1	0.025	0.012	2
deskilling	organisation	0.003	0.182	3	0.000	1	0.000	0.000	0.737	2	0.082	0.020	4
job turnover	organisation	0.086	0.177	8	0.001	6	0.799	0.799	0.815	2	0.049	0.005	9
age of homecare staff	homecare staff	0.000	0.176	3	0.000	0	0.000	0.000	0.000	3	0.074	0.018	4
self-perceived health	homecare user	0.049	0.224	5	0.034	4	0.910	0.910	0.701	3	0.180	0.030	6
quality of life	homecare user	0.001	0.008	6	0.060	6	0.787	0.787	0.000	1	0.016	0.002	7
role conflict	organisation	0.005	0.164	4	0.007	3	0.927	0.927	0.777	2	0.057	0.011	5
self-sufficiency needs	homecare user	0.002	0.195	2	0.034	2	0.850	0.850	0.870	1	0.131	0.044	3
uncertain outlook, continuity of financial burden	social support	0.000	0.126	1	0.000	0	0.000	0.000	0.924	1	0.025	0.012	2
needs met	homecare user	0.285	0.298	22	0.109	17	0.742	0.742	0.668	15	0.270	0.012	23
human resource primacy	organisation	0.000	0.149	2	0.000	1	0.000	0.000	0.858	1	0.033	0.011	3

Table S6. Causal loop diagram – connections between elements and their type.

From	To	Type	From	To	Type	From	To	Type	From	To	Type
organisational slack	number of care recipients per carer	-	instrumental activities of daily living (iADL) needs	acceptable living environment	--	functional ability and autonomy	activities of daily living (ADL) needs	-	functional ability and autonomy	self-perceived health	++
burnout	sickness absence	+	social needs	needs met	--	role conflict	job satisfaction	--	positive social climate	person-centred care	+
time spent in care	adverse events or progression of disease	+	adverse events or progression of disease	physical status	-	control	boredom or underload	+	control over decision making	ability to follow conscience	+
distress	person-centred care	-	language proficiency	confirming homecare staff - user/family communication and relationship	+	homecare staff health and safety risk	ability to cope	-	burnout	job involvement	-
county level unemployment	job turnover	-	complexity of work tasks	control over decision making	++	trust in homecare staff	needs met	++	job satisfaction	ability to cope	+
adverse events or progression of disease	mental status	-	children	social support network of homecare user	+	sense homecare user needs	ability to balance needs of homecare user and their families	+	confirming homecare staff - user/family communication and relationship	homecare user influence	+

social support network of homecare user	informal care of homecare user	+	distress	sympatic nerve system activation	+	job involvement	job satisfaction	++	functional ability and autonomy	instrumental activities of daily living (iADL) needs	-
human resource primacy	commitment to organisation	+	mental health	ability to cope	+	role clarity	role conflict	-	mastery of work and professional competence	provision of care and services	+
person-centred care	confirming homecare staff - user/family communication and relationship	+	commitment to organisation	job satisfaction	+	mental health	burnout	--	satisfaction with care	quality of life	++
confirming homecare staff - user/family communication and relationship	provision of care and services	+	functional ability and autonomy	social isolation	--	deskilling	complexity of work tasks	-	homecare user influence	quality of care	+
empowering leadership	quality of supervision	++	long-term physiological health	burnout	-	uncertain outlook, continuity of financial burden	quality of life of informal caregiver	-	job demand	boredom or underload	-

functional ability and autonomy	treatment-related needs	-	job demand	perceived work overload	+	mastery of work and professional competence	ability to balance needs of homecare user and their families	+	quality of supervision	support from coworkers	+
workload	decision demands	+	stress of conscience	ability to cope	-	control	job demand	--	job turnover	continuity of care	-
functional ability and autonomy	quality of life	+	provision of care and services	needs met	-+	working with sophisticated technology	complexity of work tasks	+	empowering leadership	control over decision making	+
workload	work pace	+	provision of care and services	acceptability of care	+	control over decision making	decision demands	++	needs met	satisfaction with care	+
other relatives, neighbours and friends	social support network of homecare user	+	mastery of work and professional competence	confirming homecare staff - user/family communication and relationship	+	perceived usefulness of technology	user engagement in development and implementation of technology	+	mental health	sickness absence	-
quality of life of informal caregiver	social support network of homecare user	+	age of homecare staff	mental health	-	informational needs	needs met	--	needs met	quality of life	+

provision of care and services	workload	-+	job satisfaction	person-centred care	+	self-efficacy of homecare staff	control over decision making	+	years of work experience	mastery of work and professional competence	+
positive challenges at work	positive social climate	+	deskilling	training and specialisation	-	recovery	sympatic nerve system activation	-	learning demands	job demand	+
self-perceived health of informal caregiver	quality of life of informal caregiver	+	positive challenges at work	commitment to organisation	+	age of homecare user	time spent in care	+	self-sufficiency needs	needs met	--
functional ability and autonomy	self-sufficiency needs	-	role clarity	positive challenges at work	+	provision of care and services	continuity of care	+	distress	mental health	-
decision demands	job demand	+	homecare staff-user adoption of technology	person-centred care	+	long-term physiological health	sickness absence	-	number of care recipients per carer	workload	+
emotional needs	needs met	--	emphasis on cost-effectiveness	deskilling	+	control	perceived work overload	-	sense homecare user suffering	ability to balance needs of homecare user and their families	+
age of homecare staff	long-term physiological health	-	provision of care and services	accessibility of care	+	long-term physiological health	ability to cope	+	experience in care	quality of life of informal caregiver	-
workload	quantitative demands	+	stress of conscience	job satisfaction	-	work pace	quantitative demands	+	quality of supervision	role clarity	+

homecare staff-user adoption of technology	coordination with other services	+	support from superior	user engagement in development and implementation of technology	+	perceived work overload	distress	+	social support network of homecare user	social isolation	-
quality of supervision	recognition	+	support provided by others	quality of life of informal caregiver	+	control over decision making	positive challenges at work	+	coordination with other services	person-centred care	+
informal care of homecare user	needs met	+	training and specialisation	mastery of work and professional competence	+	confirming homecare staff - user/family communication and relationship	user engagement in development and implementation of technology	+	recovery	parasympatic nerve system activation	+
functional ability and autonomy	informational needs	-	mastery of work and professional competence	ability to cope	+	needs met	informal caregiver stress	-	inadequate income	job satisfaction	-
language fluency	ability to communicate	+	job uncertainties	job turnover	+	boredom or underload	distress	+	family-work conflict	turnover intention	+

positive challenges at work	job involvement	+	confirming homecare staff - user/family communication and relationship	trust in homecare staff	++	likelihood of institutional care	quality of life	-	functional ability and autonomy	social needs	-
functional ability and autonomy	homecare staff-user adoption of technology	+	functional ability and autonomy	emotional needs	-	age of homecare staff	years of work experience	+	continuity of care	needs met	+
mental health	stress of conscience	-	county per capita income	job turnover	-	acceptable living environment	quality of life	+	staff shortage	number of care recipients per carer	+
staff shortage	overtime and unscheduled work	+	stigmatisation towards profession	job turnover	+	turnover intention	job turnover	+	recognition	job satisfaction	+
personal life demands	family-work conflict	+	quality of care	trust in homecare staff	+	spouse or partner	social support network of homecare user	+	homecare user influence	empowerment	+
distress	quality of care	-	parasympatic nerve system activation	long-term physiological health	+	instrumental activities of daily living (iADL) needs	needs met	--	quality of supervision	human resource primacy	+

quality of supervision	commitment to organisation	+	quality of care	needs met	+	empowering leadership	job involvement	+	accessibility of care	needs met	+
turnover intention	commitment to organisation	-	distance of relatives	social support network of homecare user	-	sympatic nerve system activation	long-term physiological health	-	self-perceived health	needs met	++
ability to cope	control	+	adverse events or progression of disease	likelihood of institutional care	+	confirming homecare staff - user/family communication and relationship	sense homecare user suffering	+	county care capacity	job turnover	+
self-perceived health	quality of life	+	distress	parasympatic nerve system activation	-	complexity of work tasks	positive challenges at work	+	provision of care and services	suitability of care	+
availability of care	needs met	+	family-work conflict	work-life balance	-	mental status	quality of life of informal caregiver	+	likelihood of institutional care	healthcare spending	+
sense homecare user needs	moral burden	+	emphasis on cost-effectiveness	organisational slack	-	overtime and unscheduled work	family-work conflict	+	distress	job involvement	-

emphasis on cost-effectiveness	inadequate income	+	ability to communicate	confirming homecare staff - user/family communication and relationship	+	informal caregiver stress	self-perceived health of informal caregiver	-	positive social climate	job satisfaction	+
quantitative demands	job involvement	-	needs met	adverse events or progression of disease	-	fair leadership	quality of supervision	+	empowerment	self-perceived health	+
workload	role conflict	+	complexity of work tasks	learning demands	+	provision of care and services	availability of care	+	working with sophisticated technology	equipment problems	+
self-efficacy of homecare user	self-perceived health	+	complexity of work tasks	decision demands	+	training and specialisation	job involvement	+	needs met	stress of conscience	-
physical status	functional ability and autonomy	++	provision of care and services	homecare staff health and safety risk	+	shift work	family-work conflict	+	moral burden	stress of conscience	+
job involvement	provision of care and services	-+	job turnover	staff shortage	+	positive social climate	recognition	+	equipment problems	workload	+

confirming homecare staff - user/family communication and relationship	sense homecare user needs	+	high or misaligned expectations	satisfaction with care	-	empowering leadership	job satisfaction	+	work-life balance	recovery	+
female	social support network of homecare user	+	ability to balance needs of homecare user and their families	ability to follow conscience	+	suitability of care	needs met	+	job satisfaction	turnover intention	-
treatment-related needs	needs met	--	social isolation	ability to communicate	-	positive challenges at work	job satisfaction	+	homecare staff-user adoption of technology	working with sophisticated technology	+
support from superior	quality of supervision	+	role conflict	ability to cope	-	social support	family-work conflict	-	quantitative demands	job demand	+
job involvement	confirming homecare staff - user/family communication and relationship	+	homecare staff-user adoption of technology	training and specialisation	++	mental status	functional ability and autonomy	++	functional ability and autonomy	ability to communicate	+

user engagement in development and implementation of technology	homecare staff-user adoption of technology	+	ability to follow conscience	provision of care and services	+	acceptability of care	needs met	+	sense homecare user suffering	moral burden	+
ability to follow conscience	moral burden	-	support from coworkers	positive social climate	+	job involvement	ability to cope	+	social support network of homecare user	ability to communicate	++
confirming homecare staff - user/family communication and relationship	homecare staff health and safety risk	-	commitment to organisation	job involvement	+	activities of daily living (ADL) needs	needs met	--			

+: positive relationship; ++: mutually positive relationship; - negative relationship; -- mutually negative relationship.
