# Supplementary material for: Investigating the Connections Between Delivery of Care, Reablement, Workload, and Organizational Factors in Home Care Services: Mixed Methods Study
Source: JMIR Hum Factors. 2023 Jun 30;10:e42283. doi: 10.2196/42283 (PMC10365606; doi:10.2196/42283)
Supplement: Multimedia Appendix 4 [file humanfactors_v10i1e42283_app4.pdf]

```
clear;
%% simulation algorithm for causal loop diagram, exported to *.xlsx from
%% kumu.
%% CLD_simulation_run.m

%% input conditions
% import CLD model structure information from Kumu xlsx export file
DataElements = readtable('CLD Model Output.xlsx','Sheet','Elements');
DataElements = sortrows(DataElements,"Type");
DataConnections = readtable('CLD Model Output.xlsx','Sheet','Connections');

% create output labels
ElementLabels=DataElements.Label;
ElementTypes=DataElements.Type;


% input parameters
XYdef = 0;          % element: baseline value, all
XYact = 1;          % element: activation value (specified)
XYbet = 0.7;        % connections: coefficient of relationships, all
SimIter = 10;       % simulation: number of iterations in simulation run

ActElem = 'workload';  % simulation: activated element

% key elements to tested in simulation study:
% The reablement approach: person-centred care
% workload: workload
% workload: distress
% technology: homecare staff-user adoption of technology
% e.g.: ActElem = 'person-centred care';


%% setup simulation model from Kumu data
% read elements
Nelements = length(DataElements.Label);

DimMatrixX = floor(sqrt(Nelements));
DimMatrixY = ceil(sqrt(Nelements));
Nmat = DimMatrixX*DimMatrixY;

Ntemp = Nmat - Nelements;
Vtemp = cell(Ntemp,1);

ElementsMat = zeros(DimMatrixX,DimMatrixY);
ElementsVecName = [DataElements.Label; Vtemp];
ElementsMatName = reshape(ElementsVecName,DimMatrixX,DimMatrixY);

% Effector -> effect arrays
EffectorVecName = DataConnections.From;
EffectVec = DataConnections.To;
EffectType = DataConnections.Type;
% read connections
Nconnections = length(DataConnections.Type);

% Simulation matrix
SimMat = zeros(DimMatrixX,DimMatrixY,Nelements);

for i=1:Nconnections
    iEffector = EffectorVecName{i};
    iEffect = EffectVec{i};
    iType = EffectType{i};
    xEffector = strcmp(ElementsVecName,iEffector);
```

```
    DimXeffector = find(xEffector,1);

    xEffect = strcmp(ElementsVecName,iEffect);
    DimXeffect = find(xEffect,1);

    if EffectType{i} == '+'
        xEff = strcmp(ElementsMatName,iEffect);
        SimMat(:,:,DimXeffector) = SimMat(:,:,DimXeffector) + xEff;

    elseif EffectType{i} == '-'
        xEff = strcmp(ElementsMatName,iEffect);
        SimMat(:,:,DimXeffector) = SimMat(:,:,DimXeffector) - xEff;

    elseif EffectType{i} == '++'
        xEff = strcmp(ElementsMatName,iEffect);
        SimMat(:,:,DimXeffector) = SimMat(:,:,DimXeffector) + xEff;

    elseif EffectType{i} == '--'
        xEff = strcmp(ElementsMatName,iEffect);
        SimMat(DimXeffector,:,:) = SimMat(:,:,DimXeffector) - xEff;

        % mutual case
        xRev = strcmp(ElementsMatName,iEffector);
        SimMat(:,:,DimXeffect) = SimMat(:,:,DimXeffect) - xRev;

    elseif EffectType{i} == '+-'
        xEff = strcmp(ElementsMatName,iEffect);
        SimMat(:,:,DimXeffector) = SimMat(:,:,DimXeffector) - xEff;

        % mutual case
        xRev = strcmp(ElementsMatName,iEffector);
        SimMat(:,:,DimXeffect) = SimMat(:,:,DimXeffect) + xRev;

    elseif EffectType{i} == '-+'
        xEff = strcmp(ElementsMatName,iEffect);
        SimMat(:,:,DimXeffector) = SimMat(:,:,DimXeffector) + xEff;

        % mutual case
        xRev = strcmp(ElementsMatName,iEffector);
        SimMat(:,:,DimXeffect) = SimMat(:,:,DimXeffect) - xRev;

    end
end

%% run simulation
ElementsMatSim = ...
    zeros(length(ElementsMat(:,1)),length(ElementsMat(1,:)),SimIter);

% initiate
xAct = strcmp(ElementsMatName,ActElem);
[DimXact, DimYact] = find(xAct,1);
ElementsMatSim(DimXact,DimYact,1) = XYact;

% iterate
for j=2:SimIter

    % find J activated element(s)
    ji=j-1;
    [rowj,colj] = find(ElementsMatSim(:,:,ji));
    NactIter=length(rowj);

    % circle through one element at a time and activate next connection
```

```
    % activate
    for k=1:NactIter
        IdxK = (colj(k) - 1) * DimMatrixX + rowj(k);
        ElementsMatSim(:,:,j) = ...
            ElementsMatSim(:,:,j) + ElementsMatSim(:,:,j-1) + ...
            SimMat(:,:,IdxK).*XYbet.*ElementsMatSim(rowj(k),colj(k),ji);
    end
end

% sum across iterations
SimOutput = sum(ElementsMatSim,3);
%% output results
% plot 2-D matrix
figure();
heatmap(SimOutput);

% plot 2-D matrix with labels
SimOutRound = round(SimOutput,3);
SimOutC=arrayfun(@num2str,SimOutRound,'un',0);
data = SimOutput;

%figure()
%heatmap(data)
%title('heatmap')

fig = figure();
ax = axes(fig);
h = imagesc(ax, data);

set(ax,'XTick',1:DimMatrixX,'YTick',1:DimMatrixY)
title(ActElem)
ax.TickLength(1) = 0;

% Create heatmap's colormap
n=256;
cmap = [linspace(.9,0,n)', linspace(.9447,.447,n)', ...
    linspace(.9741,.741,n)'];
%colormap(ax, cmap);
colorbar(ax)
hold on
% Set grid lines
HMGridX=DimMatrixX+0.5;
HMGridY=DimMatrixY+0.5;

arrayfun(@(x)xline(ax,x,'k-','Alpha',1),0.5:1:HMGridX)
arrayfun(@(y)yline(ax,y,'k-','Alpha',1),0.5:1:HMGridY)

asc = [65:90, 97:122];
nLabels = DimMatrixX*DimMatrixY;

labels_temp1 = cellfun(@(x)[x, '                    '], ElementLabels,...
    'UniformOutput', false);
labels_temp2 = cellfun(@(s)s(1:20),labels_temp1,'UniformOutput',false);
Nextra = DimMatrixX*DimMatrixY - length(labels_temp2);
labels = [labels_temp2; cell(Nextra,1)];
[xTxt, yTxt] = ndgrid(1:DimMatrixX, 1:DimMatrixY);
th = text(yTxt(:), xTxt(:), labels(:), ...
    'VerticalAlignment', 'baseline','HorizontalAlignment','Center');
th2 = text(yTxt(:), xTxt(:)-0.2, SimOutC(:), ...
    'VerticalAlignment', 'baseline','HorizontalAlignment','Center');
```

```
% plot iterations
plotLabels=labels_temp2;
DelIdx=[];
profileTemp=[];
profiles=[];

l=1;

for m=1:DimMatrixY
    for k=1:DimMatrixX
        profileTemp=squeeze(ElementsMatSim(k,m,:));
        profiles(1:length(profileTemp),l)=profileTemp;
        l=l+1;
    end
end

PlotLegend=[];
colmax=max(abs(profiles));
figure();
for k=1:10
    [xmax, ymax]=max(colmax);

    PlotProfile=profiles(:,ymax);
    PlotLegend=[PlotLegend,ElementLabels(ymax)];

    if k > 5
        plot(1:SimIter,PlotProfile,"LineWidth",2,"LineStyle",":");
    else
        plot(1:SimIter,PlotProfile,"LineWidth",2);
    end

    hold on;
    colmax(ymax)=0;
end

title(ActElem);
xlabel('iteration (n)','FontWeight','bold');
ylabel('activation','FontWeight','bold');
legend(PlotLegend,"Location","southwest");
hold off;
```
