# Supplementary material for: Investigating the Connections Between Delivery of Care, Reablement, Workload, and Organizational Factors in Home Care Services: Mixed Methods Study
Source: JMIR Hum Factors. 2023 Jun 30;10:e42283. doi: 10.2196/42283 (PMC10365606; doi:10.2196/42283)
Supplement: Multimedia Appendix 5 [file humanfactors_v10i1e42283_app5.pdf]

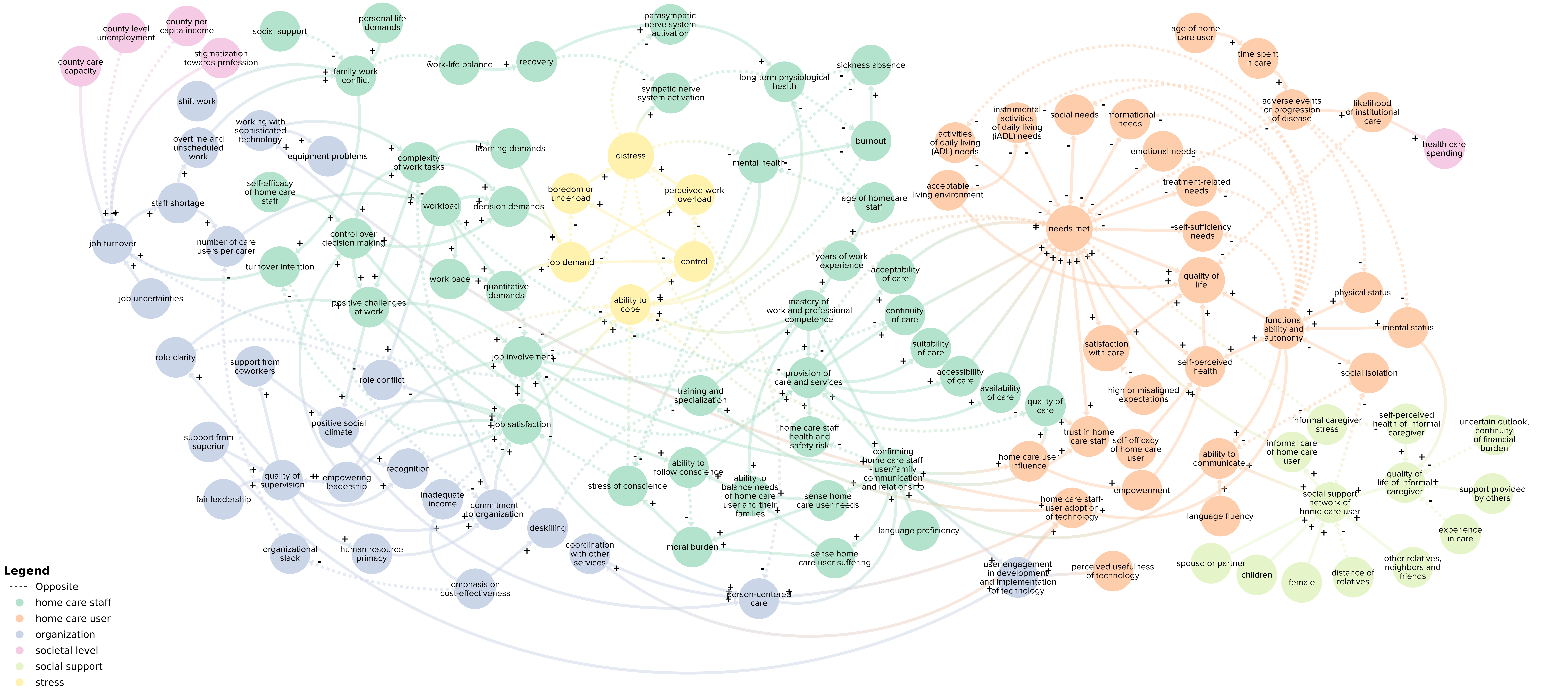
county level unemployment
county per capita income
county care capacity
stigmatization towards profession
social support
personal life demands
family-work conflict
work-life balance
recovery
parasympatic nerve system activation
sympatic nerve system activation
long-term physiological health
sickness absence
shift work
working with sophisticated technology
equipment problems
overtime and unscheduled work
complexity of work tasks
learning demands
distress
mental health
burnout
self-efficacy of home care staff
staff shortage
workload
decision demands
boredom or underload
perceived work overload
age of homecare staff
job turnover
number of care users per carer
control over decision making
job demand
control
years of work experience
turnover intention
work pace
quantitative demands
ability to cope
job uncertainties
positive challenges at work
mastery of work and professional competence
acceptability of care
continuity of care
suitability of care
accessibility of care
availability of care
quality of care
role clarity
support from coworkers
role conflict
job involvement
provision of care and services
training and specialization
positive social climate
job satisfaction
home care staff health and safety risk
confirming home care staff - user/family communication and relationship
support from superior
quality of supervision
empowering leadership
recognition
inadequate income
commitment to organization
fair leadership
ability to follow conscience
stress of conscience
ability to balance needs of home care user and their families
sense home care user needs
moral burden
sense home care user suffering
language proficiency
deskilling
coordination with other services
organizational slack
human resource primacy
emphasis on cost-effectiveness
person-centered care
age of home care user
time spent in care
adverse events or progression of disease
likelihood of institutional care
health care spending
activities of daily living (ADL) needs
instrumental activities of daily living (iADL) needs
social needs
informational needs
emotional needs
treatment-related needs
acceptable living environment
needs met
self-sufficiency needs
quality of life
physical status
functional ability and autonomy
mental status
satisfaction with care
self-perceived health
social isolation
high or misaligned expectations
trust in home care staff
self-efficacy of home care user
home care user influence
empowerment
ability to communicate
language fluency
home care staff-user adoption of technology
user engagement in development and implementation of technology
perceived usefulness of technology
informal caregiver stress
self-perceived health of informal caregiver
uncertain outlook, continuity of financial burden
informal care of home care user
quality of life of informal caregiver
social support network of home care user
support provided by others
experience in care
spouse or partner
children
female
distance of relatives
other relatives, neighbors and friends
Legend
---- Opposite
home care staff
home care user
organization
societal level
social support
stress
